# Assessing dietary intakes from household budget surveys: A national analysis in Bangladesh

**DOI:** 10.1371/journal.pone.0202831

**Published:** 2018-08-27

**Authors:** Dimitra Karageorgou, Fumiaki Imamura, Jianyi Zhang, Peilin Shi, Dariush Mozaffarian, Renata Micha

**Affiliations:** 1 Friedman School of Nutrition Science and Policy, Tufts University, Boston, MA, United States of America; 2 MRC Epidemiology Unit, University of Cambridge, Cambridge, United Kingdom; Tulane University, UNITED STATES

## Abstract

**Background:**

Accurate national information on dietary intakes, including heterogeneity among individuals, is critical to inform health implications and policy priorities. In low- and middle-income countries, household expenditure surveys constitute the major source of food data, but with uncertain validity for individual-level intakes.

**Objective:**

To investigate how individualized dietary consumption estimated from household survey data compared with individual-level 24-hr dietary recalls (24hR); and to assess potential heterogeneity by method for individualizing household intakes, dietary indicator, and individual characteristics (age, sex, education, religion, household income).

**Methods:**

We evaluated data from the 2011–2012 Bangladesh Household Integrated Survey (BIHS), which included household-level consumption data (5,503 households) and individual-level dietary data based on 24hR from these households (22,173 participants). Household and 24hR estimates were standardized and harmonized for 33 dietary indicators, including 9 food groups, total energy, 8 macronutrients, and 15 micronutrients. Individual consumption was estimated from household data using two approaches, the Adult Male Equivalent (AME) and per capita (PC) approach. For each dietary indicator, differences in household vs. individual mean estimates were evaluated overall and by strata of individual characteristics, using Spearman’s correlations and univariate and multivariate linear regression models.

**Results:**

Individualized household estimates overestimated individual intakes from 24hR for all dietary factors using either estimation method (P<0.001 for each), except for starchy vegetables (AME: P = 0.15; PC: P = 0.85). For foods, overestimation ranged from 4% for seafood to about 240% for fruits, and for nutrients from 11% for carbohydrates and poly-unsaturated fats to 55% for vitamin C, with similar overestimation for the AME and the PC method. By strata, overestimation was modestly higher in men vs. women, in children (0-10y) vs. adolescents (11-19y) and adults (20-44y, ≥45y), among adults of higher (≥6y) vs. lower (<6y) education, in Muslims vs. other religions (Christians, Hindus), and for the lowest vs. all other income groups. This overestimation was notably higher in young children (0-5y) vs. all other age groups and in the lowest vs. all other income groups. Underestimation was rarely observed, for example for milk intake (-56%) in young children (0-5y). The PC approach did not capture heterogeneity in validity of estimation of different dietary factors by age, mainly in children (0-5y, 6-10y). Spearman's correlations between individualized household estimates and 24hR data were higher for the AME (0.30–0.70) than PC (0.20–0.50) approach. Findings were similar with and without multivariate regression, with proportions of variance (R^2^) in 24hR intakes explained by the AME being generally greater than PC estimates, yet still low to modest.

**Conclusions:**

In this national survey, established methods for estimating individual level intakes from household surveys produce overestimation of intakes of nearly all dietary indicators, with significant variation depending on the dietary factor and modest variation depending on individual characteristics. These findings suggest a need for new methods to estimate individual-level consumption from household survey estimates.

## Introduction

The major global health and economic impacts of food insecurity and undernutrition have been recognized, now joined by tremendous diet-induced burdens of non-communicable diseases (NCDs) [1–5]. In nearly every region of the world, suboptimal diet is a leading modifiable risk factor for mortality and morbidity, exceeding the burdens attributable to most other global health challenges [6–8]. Even modest dietary changes are associated with improvements in maternal and child undernutrition and micronutrient deficiencies [9–13], as well as meaningful reductions in NCDs [14–17]. Based on the crucial role of nutrition in health, a better understanding of patterns and distributions of dietary habits globally is critical to inform and establish dietary priorities and improvement goals [6, 18].

For most countries around the world [18, 19], particularly low- and middle-income countries (LMIC), limited survey data are available on individual-level dietary intakes. Because of this, methods have been developed to utilize all available individual-level dietary data worldwide, together with estimates of national food-supply availability (food balance sheets) from the UN Food and Agriculture Organization (FAO) [20–22], to estimate individual-level dietary intakes in every country globally [6, 19, 23–27]. Yet, the utility of another major potential source of dietary information, household-level consumption and expenditure surveys (HCES), is not well established. Household surveys have the advantage of being done regularly (typically every 3–5 years in most countries) and including large samples, providing potentially relevant data to augment existing estimates of individual-level dietary intakes. However, such surveys are designed mainly to evaluate financial and living conditions of households, rather than specifically for nutrition or food habits; and also collect data on overall household food consumption, not individual intakes [28–30]. Other potential limitations of household surveys include short recall reference periods, a limited number of foods assessed, absence of data on within-household distributions of intakes, and insufficient accounting for food waste, for foods acquired for purposes other than household consumption (e.g., for storage, guests, livestock), for foods consumed away from home, and for cooking effects on food weight and nutrient content [30–33]. As such, their potential validity for estimating individual-level dietary intake distributions, as well as potential variability in this validity according to individual characteristics such as age, sex, education, or income, is not established.

Two main methods have been proposed for estimating individualized dietary intakes from household data, including the adult male equivalent (AME) and the per capita (PC) approach [20, 34–42]. The PC approach assumes that each person within the household has equal access to and intake of food, while the AME assumes that each person’s intake is proportional to their age and sex-specific caloric requirements. However, the validity of such approaches in predicting individual dietary intakes, including for total caloric/energy intake, major foods, macro- and micronutrients and by various population subgroups, remains uncertain. Few prior studies have used and tested the PC [39] or AME approach [34, 35, 40, 41]. These have focused on total energy and a limited number of macro- or micro-nutrients (e.g., protein, fat, fiber, iron) [34, 40, 41] or specific foods associated with malnutrition [35, 39]; have included only specific population groups, such as women of reproductive age and young children (up to 5y) [34, 35] rather than the general population [39–41]; and have used heterogeneous sources of household-level dietary data, including acquisition [35, 39], consumption [34], or computed from individual intakes [40, 41]. Multiple other foods, macronutrients and micronutrients linked to the double burden of malnutrition, as well as potential differences by individual characteristics or method of estimation (PC, AME) have not been evaluated. To address these gaps in knowledge, we compared estimates of individualized dietary intakes from household data to 24-hr recall (24hR) dietary estimates among the same individuals in the nationally representative Bangladesh Integrated Household Survey (BIHS). We evaluated validity overall as well as according to method of estimation (AME, PC), dietary factor, and key individual characteristics.

## Methods

### Dietary survey

We utilized the BIHS 2011–2012 [43], a comprehensive nationally representative survey from a LMIC that includes both household survey and individual-level 24hR dietary estimates from the same individuals. BIHS data are publicly available [43], and household-level dietary data further meet the International Household Survey Network (IHSN) reliability and relevance assessment criteria (**Table A in S1 File**) [30]. BIHS used a two-stage stratified sampling design and covered a total of 6,503 households including 27,285 individuals (47.6% men, 0–120 years, mean age 26.6 (SD: 19.9) years) [43, 44]. Of those, 5,503 households were representative of the rural Bangladesh and 2,040 of southwest Bangladesh as part of the Feed the Future (FTF) global hunger and food security initiative; 1,040 households contributed to the representativeness of both national and FTF zone samples [44]. For the present analysis, and in line with local experts, we used the BIHS national sample, which included 5,503 households and 23,135 individuals. Of these, we excluded 962 individuals with missing 24hR data that were not home at the time of the interview, did not report any consumption of foods or beverages for unknown reasons, or were exclusively breastfed babies. Excluded individuals did not differ in key sociodemographics compared to the overall sample, yet as expected, they were younger (17.0±19.6 years), since 26% were exclusively breastfed babies. The final analytical sample consisted of 5,503 households and 22,173 individuals (**Table 1**).

**Table 1:** Characteristics of the 2011–2012 Bangladesh Integrated Household Survey (BIHS)^1^.

	BIHS sample for analysis
Representativeness	National of rural Bangladesh
Sample size	5,503 households
	22,173 individuals
Sex (n, %)	
Men	10,502 (47.4%)
Women	11,671 (52.6%)
Age (years)	
Range	0–120
Mean (SD)	26.8±19.8
Age groups (n, %)	
0–5	2,807 (12.7%)
6–10	3,078 (13.9%)
11–19	3,885 (17.5%)
20–44	7,728 (34.8%)
≥45	4,675 (21.1%)
Education (n, %)^2^	
<6 years	8,934 (72.0%)
≥6 years	3,469 (28.0%)
Religion (n, %)^3^	
Muslims	19,735 (89%)
Other	2,438 (11%)
Household income (n, %)	
1^st^ quintile	4,441 (20.0%); 71% of low education
2^nd^ quintile	4,450 (20.1%); 79% of low education
3^rd^ quintile	4,429 (20.0%); 78% of low education
4^th^ quintile	4,420 (19.9%); 72% of low education
5^th^ quintile	4,433 (20.0%); 61% of low education
Sampling design	Two-stage stratification; selection of PSUs and selection of households within each PSU (2001 population census–adjusted for 2011 population census)
Seasonality	December 15 –March 15, 2012 (national sample) “Monga” periods are not covered [82] ^4^
Training of the researchers	Bangladeshi consulting firm with expertise in complex surveys and data analysis. IFPRI researchers and the consulting firm experts trained experienced enumerators, researchers, and editors to edit the completed questionnaires during the survey.
Food groups included in the food list^5^	Cereal (17), Pulses (9), Edible oil (7), Vegetables (43), Leafy vegetables (31), Meat/Eggs and milk (16), Fruits (31), Fish (65), Spices (20), Drinks and beverages (5), Other foods (6), Other foods prepared outside home (37)

^1^ The reported descriptive statistics are unweighted.

^2^ Estimated only for the adult population (≥20 years old).

^3^ Religion was reported only for household head. We assumed that other household members were of the same religion. Other religions refer to Christians and Hindus.

^4^ “Monga” is most commonly defined as a famine-like state that occurs twice per year; a severe period from mid-September to mid-November, and a less severe from mid-March to mid-May. It affects mainly northwestern Bangladesh.

^5^ The household consumption questionnaire included a list of selected food items, organized in food groups.

IFPRI, International Food Policy Research Institute; PSU, Primary sampling units

24hR data were collected in-person by trained interviewers using an open-ended recall. The household member responsible for preparing the meals (women 98.8%, 36.2±12.3 years) reported the foods (single-ingredient, mixed dishes) consumed during the previous day in the household from any source, including own cooking, purchased foods, and gifts. Information was collected on the total “as consumed” weight of each food item, the disaggregated ingredients and corresponding raw weights in mixed dishes, and on how much of these food items was consumed by each household member, stored as leftovers, thrown away, or given to guests, others, and animals/livestock.

Household-level consumption was assessed by a 7-day 287-food item questionnaire, including 37 food items for food consumed away from home. Foods consumed and their quantity for the household (raw weight for single-ingredient foods; cooked weight for mixed dishes) were reported by the same household member as for the 24hR. Dietary data collection was performed from December 2011 to March 2012. Details on the BIHS administration and questionnaires can be found elsewhere [43].

### Dietary dataset harmonization

Dietary data were harmonized within and between the 24hR and household datasets. This process involved 7 key steps (**Appendix A in S1 File**): *1*. *dataset retrieval*, involving identification and retrieval of relevant dietary and sociodemographic BIHS datasets and variables; *2*. *unique food item identification and description*, identifying the unique food items (single-ingredient or disaggregated ingredient) across the diet assessment methods by matching their available food description, further accounting for food consumed away from home; *3*. *food matching*, matching food items to available food composition data [45, 46] for nutrient profiling (if nutrient composition was available for the overall recipe/mixed dish then that was preferred) further accounting for alterations in nutrient content during cooking (use of retention factors [45, 47, 48]) [49, 50]; *4*. *unit standardization*, accounting for non-edible portions and cooking alterations (use of yield factors [45, 47, 51, 52]), converting and reporting in standardized “as consumed” metrics, i.e., g/day for foods and macronutrients (other than cholesterol), and mg/day or μg/day for micronutrients; *5*. *food classification*, classifying unique food items (including disaggregated ingredients) to food groups (e.g., fruits, vegetables) using previously established methods [19, 53, 54]; *6*. *individualization of household consumption*, where household food and nutrient consumption was individualized by the AME [20] and PC [55] approach (**Appendix B in S1 File**); and, *7*. *final dataset preparation*, merging and creating a complete dataset including individual-level dietary and sociodemographic information. Local experts provided advice on each of those steps, particularly for steps 2, 3, and 5.

The AME method [20] was our primary approach for individualizing household consumption [34, 35, 37]. This method assumes that the intra-household food distribution is proportional to the individual’s share of total household energy requirements, and as such household members do not receive an equal share of the food available for consumption. The energy requirements of household members of different age, sex, and status (pregnant/ lactating women) were expressed in proportion to an adult male’s energy requirements (**Appendix B in S1 File**). In secondary analysis, we used the PC approach [56] to estimate the per capita consumption, assuming that the available food in the household is equally distributed among household members.

### Selection of key dietary targets

We included dietary indicators that captured an individual’s intake from all sources. Among the array of dietary factors that could be assessed, we identified 48 potential dietary indicators (18 food groups, 11 macronutrients, 18 micronutrients, and total energy) based on evidence for etiological effects on a) major chronic diseases (e.g., type 2 diabetes, stroke, heart disease) and related risk factors (e.g., blood lipids, blood pressure, obesity) [7, 8, 57–62], or b) deficiency-related health conditions and mortality (e.g., anemia, blindness, maternal mortality) [1, 10, 12, 13]. Among these 48 factors, the final selection of dietary indicators was based on observed intake levels (foods) and available food composition data (nutrients) in this survey. For example, if intake for a selected food group was low, it was combined into a broader category (e.g., whole grains and refined grains were combined into total grains due to low whole grain intake), or omitted if very rarely consumed (e.g., sugar-sweetened beverages). Nutrients were not analyzed if food composition data were not available (iodine), missing for >70% of foods (trans fats, omega-6 fats, vitamin B_12_, selenium) or missing for major dietary sources (omega-3 contents for seafood).

In sum, 33 dietary indicators were included in the final analysis (**Table B in S1 File**), including 9 food groups (fruits, non-starchy vegetables, starchy vegetables, legumes, total grains, meat/eggs, seafood, whole-fat milk, fats/oils), total energy, 8 macronutrients (protein, carbohydrates, total fats, saturated fatty acids (SFA), monounsaturated fatty acids (MUFA), polyunsaturated fatty acids (PUFA), cholesterol, fiber), and 15 micronutrients (vitamins A, B_1_ (thiamine), B_2_ (riboflavin), B_3_ (niacin), B_6_, B_9_ (folate), C, D, and E, sodium, potassium, magnesium, calcium, iron, zinc).

### Statistical analysis

Average dietary consumption was estimated and compared for the individual 24hR intakes and individualized household estimates overall and by population strata, including by age (≤5, 6–10, 11–19, 20–44, and ≥45 years), sex (men, women), education (<6 years of education, ≥6 years of education), religion (Muslims, other religions), and monthly household income (quintiles). To assess how well individualized household estimates ranked participants in comparison to 24hR estimates, Spearman’s correlations were used.

To assess differences in dietary means and also the proportion of variance explained, the relation between 24hR and AME estimates was assessed by using univariate and multivariable random-intercept linear regression analysis which accounted for household clusters [19].

$${24hR\;estimate}_{ij}=\beta_{0}+\beta \times {AME\;estimate}_{ij}\;(univariate\;model)$$

$${24hR\;estimates}_{ij}=\beta_{0}+\beta \times {AME\;estimate}_{ij}+\beta^{\prime }\times {covariates}_{ij}\;(multivariable\;model)$$

where 24hR estimate_ij_ represents intake estimates for individual *i* and household *j*; β_0_ represents the intercept; β (slope) represents the difference in the 24hR mean for a 1-unit difference in the AME mean; AME estimate_ij_ represents individualized intake estimates for individual *i* and household *j*; covariates_ij_ were covariates specific to individual *i* and household *j*; β’ represents a set of regression coefficients for differences in the 24hR mean for a 1-unit difference in covariates; and random effects were modeled for β_0_ and fixed effects were modeled for βs. Analyses were repeated for the PC approach. For the multivariable models, we selected covariates which would be available in household surveys, including basic demographics, such as age and sex, in the minimally adjusted model, and additionally education, religion, household income, respondent’s characteristics (age, sex, education), and household characteristics (household size, number of children, and wastage percentage) in the fully adjusted model.

For the aim to assess potential heterogeneity, regression analyses (univariate, multivariate) were performed by sex, age, sex and age, education, religion, and household income. There was no correlation between education and income, as assessed by Cohen's kappa coefficient (κ = -0.02), which justified stratified analysis by each variable separately. Stratification by all demographic factors was not performed because of low sample size and unstable estimates in some strata. Missing covariate values for education (n = 17, 0.0008%) were imputed with a single regression imputation as the missing values were very few, assuming education was missing at random [63], and using age, sex, and household size as predictors; predictors had no missing values.

Analyses were performed using STATA 14 (College Station, TX: StataCorp LP). Results from statistical tests were considered significant with two-sided alpha = 0.05.

## Results

### Findings for overall population

Participants were 47.4% men, less educated (72% with <6 years of education), mainly Muslims (89.0%), and had mean (SD) monthly household income of 6,701 (9,339) BDT (79.4 (110.6) USD/month) **(Table 1)**. Their diet was characterized by staples consisting primarily of total grains (e.g., rice, flour), starchy vegetables (mainly potatoes), non-starchy vegetables, and seafood (mainly fish) (**Table 2**).

**Table 2:** Comparison of individualized household consumption and individual dietary estimates by dietary factor in the 2011–2012 BIHS.

Dietary Factor, unit^1^	Observations (n)^2^	Consumption (mean, SD)^3^			Correlation (rho coefficient)^4^		Difference between 24hR and AME (mean, SD)^5^		Difference between 24hR and PC (mean, SD)^5^	
		AME ^3^	PC ^3^	24hR ^3^	24hR-AME	24hR-PC	Absolute^6^	Percent	Absolute^7^	Percent
**Food groups**										
Fruits, g/d	22,146	30.8 (53.1)	30.5 (50.5)	9.0 (35.7)	0.29	0.30	21.8 (56.3)	242	21.5 (54.0)	239
Non-starchy vegetables, g/d	22,173	260.3 (166.8)	258.4 (150.6)	167.3 (147.2)	0.46	0.32	92.9 (174.1)	56	91.0 (176.6)	54
Starchy vegetables, g/d	21,919	105.6 (69.4)	104.8 (62.6)	104.7 (97.8)	0.41	0.31	0.9 (95.4)	1	0.1 (99.0)	0
Legumes, g/d	19,885	22.5 (32.3)	22.3 (30.9)	20.1 (50.2)	0.39	0.39	2.4 (48.9)	12	2.2 (49.1)	11
Total grains, g/d	13,310	1473.7 (536.7)	1463.4 (399.9)	1367.2 (563.9)	0.69	0.37	106.5 (430.3)	8	96.1 (561.1)	7
Meat/Eggs, g/d	15,019	17.2 (20.5)	17.1 (19.5)	11.8 (26.3)	0.36	0.37	5.4 (26.9)	46	5.2 (26.7)	44
Seafood, g/d	22,173	25.7 (24.7)	25.4 (23.1)	24.6 (29.9)	0.50	0.44	1.1 (28.6)	4	0.9 (29.2)	4
Milk, whole fat, g/d	21,201	26.3 (54.1)	26.2 (52.0)	17.7 (60.7)	0.51	0.52	8.6 (58.2)	49	8.4 (54.8)	47
Fats/Oils, g/d	22,162	20.2 (13.2)	20.1 (12.0)	17.8 (16.0)	0.56	0.43	2.4 (15.2)	13	2.3 (15.7)	13
**Energy & Macronutrients**										
Energy, kcal/d	22,173	2322.4 (871.1)	2305.4 (654.5)	2064.6 (818.7)	0.69	0.36	257.8 (679.5)	12	240.7 (847.0)	12
Protein, g/d	22,173	56.5 (23.6)	56.1 (18.9)	50.0 (22.3)	0.67	0.39	6.5 (19.8)	13	6.1 (23.2)	12
Carbohydrates, g/d	22,173	444.1 (164.2)	440.8 (121.9)	397.7 (158.5)	0.68	0.35	46.3 (129.4)	12	43.1 (163.8)	11
Total fat, g/d	22,173	30.0 (18.6)	29.8 (16.7)	25.6 (19.7)	0.61	0.50	4.4 (18.7)	17	4.1 (19.3)	16
SFA, g/d	22,173	6.5 (5.4)	6.5 (5.0)	5.3 (4.8)	0.62	0.52	1.2 (5.5)	23	1.2 (5.4)	23
MUFA, g/d	22,173	9.1 (5.6)	9.0 (5.0)	7.8 (6.0)	0.61	0.49	1.3 (5.5)	17	1.3 (5.7)	17
PUFA, g/d	22,173	13.7 (9.0)	13.6 (8.1)	12.2 (10.3)	0.67	0.55	1.5 (9.3)	12	1.4 (9.7)	11
Cholesterol, mg/d	22,172	43.4 (46.6)	43.0 (44.0)	33.4 (58.8)	0.47	0.46	9.9 (59.7)	30	9.6 (58.9)	29
Fiber, g/d	22,173	31.4 (12.7)	31.1 (9.9)	26.0 (11.4)	0.65	0.34	5.4 (10.6)	21	5.1 (12.5)	20
**Vitamins**										
Vitamin A, μg RAE/d	22,173	322.8 (356.2)	320.5 (336.9)	214.8 (404.8)	0.41	0.38	107.9 (439.4)	50	105.6 (438.7)	49
Vitamin D, μg/d	22,173	1.3 (1.9)	1.3 (1.8)	1.1 (2.7)	0.47	0.46	0.2 (2.5)	18	0.2 (2.6)	18
Vitamin E, mg/d	22,173	5.4 (3.2)	5.3 (2.8)	4.5 (3.4)	0.67	0.55	0.9 (3.1)	20	0.8 (3.2)	18
Thiamine, mg/d	22,173	0.9 (0.4)	0.9 (0.3)	0.8 (0.4)	0.63	0.36	0.2 (0.3)	25	0.2 (0.4)	25
Riboflavin, mg/d	22,173	0.6 (0.3)	0.6 (0.2)	0.5 (0.2)	0.60	0.41	0.1 (0.3)	20	0.1 (0.3)	20
Niacin, mg/d	22,173	16.0 (6.4)	15.9 (5.0)	14.1 (6.2)	0.73	0.45	1.9 (4.8)	13	1.8 (6.0)	13
Vitamin B6, mg/d	22,173	1.4 (0.7)	1.4 (0.6)	1.2 (0.7)	0.69	0.46	0.2 (0.5)	17	0.2 (0.6)	17
Folate, μg/d	22,173	157.3 (82.1)	156.1 (71.0)	120.5 (77.2)	0.53	0.33	36.8 (84.0)	31	35.6 (87.1)	30
Vitamin C, mg/d	22,173	65.4 (45.3)	65.0 (41.3)	42.2 (40.7)	0.41	0.27	23.3 (49.7)	55	22.8 (49.7)	54
**Minerals**										
Calcium, mg/d	22,173	343.4 (207.8)	340.8 (185.1)	274.2 (222.7)	0.58	0.43	69.2 (222.3)	25	66.6 (225.4)	24
Iron, mg/d	22,173	12.0 (5.7)	11.9 (4.8)	9.9 (5.3)	0.63	0.38	2.1 (5.1)	21	2.0 (5.6)	20
Sodium, mg/d	22,173	5855.2 (3145.8)	5812.9 (2739.4)	4225.0 (3073.7)	0.39	0.19	1630.2 (3737.0)	39	1587.9 (3790.8)	38
Potassium, mg/d	22,173	1745.6 (784.3)	1732.6 (647.3)	1394.6 (638.9)	0.63	0.36	351.0 (670.1)	25	338.0 (735.4)	24
Magnesium, mg/d	22,173	376.9 (146.7)	374.1 (112.8)	320.6 (133.4)	0.67	0.34	56.3 (119.0)	18	53.5 (143.3)	17
Zinc, mg/d	22,173	9.8 (3.8)	9.7 (2.9)	8.6 (3.5)	0.68	0.36	1.2 (3.0)	14	1.1 (3.7)	13

^1^ Dietary factors presented had adequate data/information for the present analysis (see Selection of dietary targets).

^2^ Sample sizes differ because we performed paired analysis for each dietary factor, i.e., for each analysis we used only the individuals with available intake data for both diet assessments.

^3^ Bangladesh Integrated Household Survey (BIHS) 2011–2012 provided household-level dietary data from a 7-day household consumption questionnaire and individual-level data from 24-hour recalls (24hR). Household consumption was individualized by applying a) the Adult Male Equivalent (AME) method [20] as proposed by FAO [56], assuming moderate physical activity, and b) the per capita (PC) approach assuming equal distribution among household members (**Appendix B in S1 File**). Individual intake was estimated from 24hR.

^4^ Spearman correlation coefficients (rho). All correlations were significant (P<0.001).

^5^ Absolute differences correspond to AME-24hR and PC-24hR respectively, and percentage differences correspond to absolute difference/24hR*100.

^6^ Differences between means were significant for all dietary factors (paired t-test, P<0.001), with the exception of starchy vegetables (p = 0.15).

^7^ Differences between means were significant for all dietary factors (paired t-test, P<0.001), with the exception of starchy vegetables (p = 0.85).

MUFA, Monounsaturated fats; PUFA, Polyunsaturated fats; SFA, Saturated fat

Considering relative rankings (Spearman's correlations) of individuals, correlations between individualized household estimates and 24hR data were generally higher for AME compared with PC estimates (**Table 2**). For AME estimates, correlations were generally modest (ranging from about 0.30 to 0.70) while for PC estimates correlations were lower (ranging from about 0.20 to 0.50).

**Fig 1:**
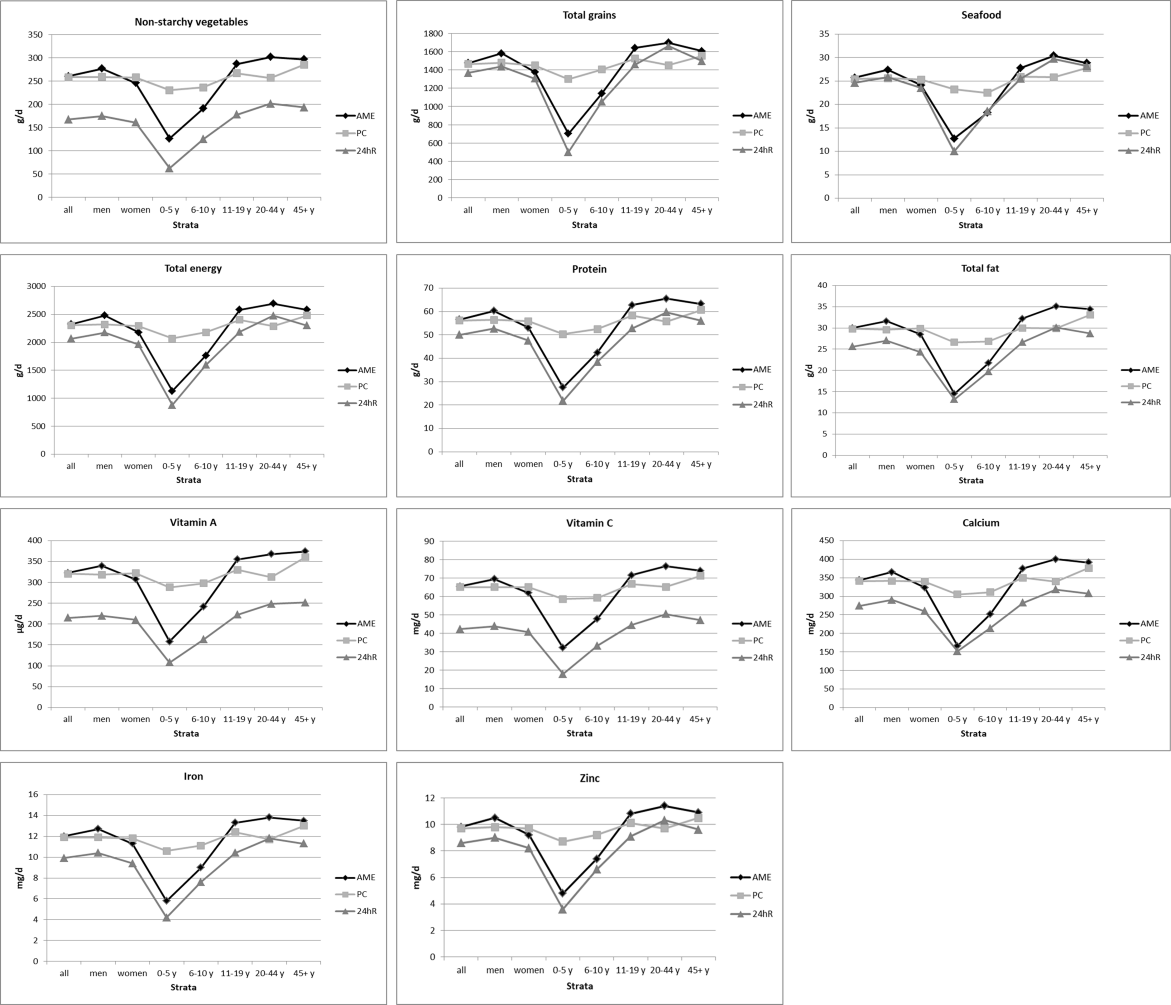
National mean individualized household estimates compared with 24-hour recall intakes as the reference measure of individual-level consumption, overall and by sex and age for selected dietary factors in the 2011–2012 BIHS. Mean individualized dietary consumption estimated from household survey data by the Adult Male Equivalent (AME) and per capita (PC) approach (Appendix B in S1 File) and individual-level 24-hr dietary recall (24hR) intakes are presented for the overall population (all), by sex (men, women), and by age (0-5y, 6-10y, 11-19y, 20-44y, 45+y). Intakes are presented in g/d for foods, and in mg/d (except for vitamin A, μg/d) for nutrients.

For all dietary indicators except starchy vegetables, mean individualized household estimates significantly exceeded individual intakes (P<0.001 each) (**Table 2**). The degree of overestimation was generally very similar between the AME and PC approaches (**Fig 1**). Among different foods, the overestimation was smallest for seafood (AME: 4%, PC: 4%) and total grains (8%, 7%), and greatest for non-starchy vegetables (56%, 54%) and fruits (242%, 239%). Total energy was overestimated by about 12%. Among macro- and micronutrients, the degree of overestimation ranged from about 11–55% and was highest for vitamins A (50%, 49%) and C (55%, 54%) and lowest for carbohydrates (12%, 11%), and protein (13%, 12%). The shape of the dietary factor distribution, including narrowness, was generally similar between the AME and 24hR estimates (**Figure A in S1 File**). The magnitude of the narrowness, as assessed by the standard deviations, was also quite similar between the two types of estimates (**Table 2**). The AME estimates were generally characterized by less variable distributions.

In unadjusted linear regression analyses, proportions of the variance (R^2^) in 24hR intakes explained by individualized household estimates (AME) were generally modest to low (**Table 3, Table C in S1 File**). For foods, for example, modest values were seen for total grains (R^2^ = 0.48), milk (0.24) and fats/oils (0.23), and lower values for fruits (0.06), legumes (0.13) and meat/eggs (0.13). For total energy and nutrients, variation explained was highest for niacin (0.50), vitamin B_6_ (0.49), energy (0.46) and carbohydrates (0.46), and lowest for sodium (0.08) and vitamin C (0.11). Proportions explained by the PC estimates were generally smaller. Adjustment for sex and age improved the variation explained for all dietary factors, mainly for energy, protein, carbohydrates, fiber, potassium and magnesium. Additional adjustments did not appreciably change observed relations.

**Table 3:** Relation between individualized household intake estimates as predictors of individual dietary intakes in the 2011–2012 BIHS^1^.

	AME			PC		
Dietary Factor, unit	Intercept (SE)	*β* (SE)	R^2^	Intercept (SE)	*β* (SE)	R^2^
**Food groups**						
Fruits, g/d						
Unadjusted	5.4 (0.5)	0.1 (0.02)	0.06	3.7 (0.5)	0.2 (0.02)	0.06
Sex and age	7.0 (0.7)	0.1 (0.02)	0.06	3.9 (0.7)	0.2 (0.02)	0.06
Multivariate	10.7 (7.0)	0.1 (0.02)	0.07	7.2 (7.0)	0.2 (0.02)	0.06
Non-starchy vegetables, g/d						
Unadjusted	29.4 (2.5)	0.5 (0.01)	0.15	96.6 (3.7)	0.3 (0.01)	0.09
Sex and age	23.8 (2.8)	0.3 (0.01)	0.19	8.0 (3.8)	0.2 (0.01)	0.18
Multivariate	14.4 (16.6)	0.3 (0.01)	0.20	-9.8 (17.0)	0.3 (0.01)	0.19
Legumes, g/d						
Unadjusted	6.1 (0.7)	0.6 (0.04)	0.13	7.8 (0.8)	0.5 (0.04)	0.12
Sex and age	2.4 (0.8)	0.5 (0.04)	0.13	-2.3 (0.9)	0.5 (0.04)	0.13
Multivariate	9.7 (11.9)	0.5 (0.04)	0.13	3.9 (11.8)	0.5 (0.04)	0.13
Total grains, g/d						
Unadjusted	86.9 (13.1)	0.9 (0.01)	0.48	626.3 (17.2)	0.5 (0.01)	0.12
Sex and age	137.1 (14.1)	0.5 (0.01)	0.57	77.2 (16.8)	0.4 (0.01)	0.53
Multivariate	207.4 (51.0)	0.6 (0.01)	0.60	42.2 (57.8)	0.4 (0.01)	0.55
Seafood, g/d						
Unadjusted	6.8 (0.5)	0.7 (0.02)	0.22	11.0 (0.7)	0.5 (0.03)	0.17
Sex and age	3.8 (0.5)	0.5 (0.02)	0.23	-1.8 (0.7)	0.5 (0.03)	0.22
Multivariate	3.2 (4.0)	0.5 (0.02)	0.23	-3.9 (4.0)	0.5 (0.03)	0.22
**Energy & Macronutrients**						
Energy, kcal/d						
Unadjusted	269.5 (15.8)	0.8 (0.01)	0.46	1052.8 (26.7)	0.4 (0.01)	0.13
Sex and age	413.2 (16.5)	0.4 (0.01)	0.55	308.4 (25.0)	0.3 (0.01)	0.51
Multivariate	498.6 (70.5)	0.5 (0.01)	0.57	237.0 (75.7)	0.4 (0.01)	0.53
Protein, g/d						
Unadjusted	7.0 (0.4)	0.8 (0.01)	0.39	25.8 (0.7)	0.4 (0.01)	0.14
Sex and age	10.0 (0.4)	0.4 (0.01)	0.47	5.9 (0.7)	0.3 (0.01)	0.43
Multivariate	11.8 (2.4)	0.5 (0.01)	0.49	4.0 (2.5)	0.4 (0.01)	0.45
Carbohydrates, g/d						
Unadjusted	44.8 (3.0)	0.8 (0.01)	0.46	204.1 (4.9)	0.4 (0.01)	0.12
Sex and age	71.4 (3.2)	0.4 (0.01)	0.55	56.9 (4.7)	0.3 (0.01)	0.51
Multivariate	80.3 (12.7)	0.5 (0.01)	0.58	35.3 (13.8)	0.4 (0.01)	0.54
MUFA, g/d						
Unadjusted	2.0 (0.1)	0.6 (0.01)	0.30	2.9 (0.2)	0.5 (0.03)	0.23
Sex and age	2.1 (0.1)	0.5 (0.02)	0.30	0.2 (0.2)	0.5 (0.03)	0.29
Multivariate	3.6 (0.7)	0.5 (0.02)	0.31	1.2 (0.7)	0.5 (0.03)	0.30
PUFA, g/d						
Unadjusted	2.3 (0.2)	0.7 (0.01)	0.29	4.7 (0.3)	0.6 (0.02)	0.21
Sex and age	2.1 (0.2)	0.6 (0.02)	0.30	-0.6 (0.3)	0.5 (0.02)	0.28
Multivariate	5.5 (1.4)	0.6 (0.02)	0.31	2.3 (1.4)	0.5 (0.02)	0.30
Fiber, g/d						
Unadjusted	2.9 (0.2)	0.7 (0.01)	0.38	14.7 (0.4)	0.4 (0.01)	0.11
Sex and age	4.6 (0.2)	0.4 (0.01)	0.48	3.9 (0.4)	0.3 (0.01)	0.45
Multivariate	5.0 (1.2)	0.4 (0.01)	0.50	2.5 (1.3)	0.3 (0.01)	0.47
**Vitamins**						
Vitamin A, μg RAE/d						
Unadjusted	64.3 (7.1)	0.5 (0.03)	0.11	98.4 (7.9)	0.4 (0.03)	0.10
Sex and age	37.0 (7.2)	0.4 (0.03)	0.12	-5.3 (8.6)	0.4 (0.03)	0.11
Multivariate	17.1 (61.0)	0.4 (0.03)	0.12	-36.3 (60.5)	0.4 (0.03)	0.11
Thiamine, mg/d						
Unadjusted	0.1 (0.01)	0.7 (0.01)	0.34	0.4 (0.01)	0.4 (0.01)	0.12
Sex and age	0.2 (0.01)	0.4 (0.01)	0.42	0.1 (0.01)	0.3 (0.01)	0.39
Multivariate	0.2 (0.04)	0.4 (0.01)	0.43	0.1 (0.04)	0.3 (0.01)	0.40
Riboflavin, mg/d						
Unadjusted	0.2 (0.01)	0.5 (0.01)	0.29	0.3 (0.01)	0.4 (0.01)	0.16
Sex and age	0.2 (0.01)	0.3 (0.01)	0.33	0.1 (0.01)	0.3 (0.01)	0.31
Multivariate	0.2 (0.02)	0.3 (0.01)	0.34	0.1 (0.02)	0.4 (0.02)	0.33
Niacin, mg/d						
Unadjusted	1.1 (0.1)	0.8 (0.01)	0.50	5.8 (0.2)	0.5 (0.01)	0.19
Sex and age	1.5 (0.1)	0.5 (0.01)	0.56	0.1 (0.2)	0.4 (0.01)	0.51
Multivariate	1.8 (0.5)	0.6 (0.01)	0.58	-0.8 (0.5)	0.5 (0.01)	0.53
**Minerals**						
Calcium, mg/d						
Unadjusted	82.6 (4.7)	0.5 (0.02)	0.22	118.4 (7.6)	0.5 (0.02)	0.16
Sex and age	88.9 (5.3)	0.4 (0.02)	0.23	23.3 (7.9)	0.4 (0.02)	0.22
Multivariate	84.4 (30.8)	0.4 (0.02)	0.23	-0.5 (31.0)	0.4 (0.03)	0.22
Iron, mg/d						
Unadjusted	1.5 (0.1)	0.7 (0.01)	0.32	5.1 (0.2)	0.4 (0.02)	0.15
Sex and age	1.9 (0.1)	0.4 (0.01)	0.38	0.9 (0.2)	0.3 (0.01)	0.35
Multivariate	1.8 (0.6)	0.4 (0.01)	0.39	0.1 (0.6)	0.4 (0.02)	0.37
Sodium, mg/d						
Unadjusted	1472.9 (53.7)	0.5 (0.01)	0.08	3284.4 (90.3)	0.2 (0.02)	0.02
Sex and age	1530.3 (55.6)	0.2 (0.01)	0.14	1361.0 (91.0)	0.1 (0.02)	0.13
Multivariate	2794.9 (570.6)	0.2 (0.01)	0.14	2574.0 (579.3)	0.1 (0.02)	0.14
Zinc, mg/d						
Unadjusted	1.1 (0.1)	0.8 (0.01)	0.43	4.6 (0.1)	0.4 (0.01)	0.12
Sex and age	1.7 (0.1)	0.4 (0.01)	0.52	1.3 (0.1)	0.3 (0.01)	0.49
Multivariate	1.9 (0.3)	0.5 (0.01)	0.54	0.9 (0.4)	0.3 (0.01)	0.51

All p-values are <0.001.

^1^ Results are presented for selected dietary factors and for overall population in the Bangladesh Integrated Household Survey (BIHS) 2011–2012. Results for all 33 dietary factors are presented in the Supplement (**Table C in S1 File**).

On the basis of linear regression models with 24-hour diet recall (24hR) intakes as the dependent variable and individualized Adult Male Equivalent (AME) or per capita (PC) consumption estimates from household questionnaire as the independent variable. The sex and age model was categorized as follows: age, ≤5, 6–10, 11–19, 20–44, and ≥45 years; sex, men and women. The multivariate model was adjusted for the following covariates: age (≤5, 6–10, 11–19, 20–44, and ≥45 years), sex (men, women), education (<6 years of education, ≥6 years of education), religion (Muslims, other), household income (quintiles), respondent’s age (continuous), sex (men, women) and education (<6 years of education, ≥6 years of education), household size, number of children within household, and food wastage percentage (using 24hR data, we calculated for each household, the percent of food wastage -sum of food waste, and food given to guests, others and animals- to total consumed food (mean: 11.6%, SD: 13.6)). *Bs* represent the change in the individual intake (24hR) for every unit increase in the respective mean of household estimates. SEs for the intercept and *βs* are presented. R^2^ represents the coefficient of determination for the overall model.

MUFA, Monounsaturated fats; PUFA, Polyunsaturated fats; SFA, Saturated fat

### Findings by sex

Overall dietary consumption patterns were broadly similar by sex, although men often had higher mean intakes across foods, especially for milk, meat/eggs, legumes, and total grains, and certain nutrients, mainly B vitamins, cholesterol, fatty acids and calcium (**Tables D and E in S1 File**). The overestimation of AME household estimates was modestly higher in men than women for most factors (**Fig 1**) except for meats/eggs, milk, and dietary cholesterol. In contrast, the PC estimates often produced higher overestimation in women than men, highest for milk (men: 32%, women: 66%), fruits (226%, 255%), meats/eggs (31%, 58%), and cholesterol (19%, 39%). In men vs. women, the variance explained for each dietary factor by individualized household estimates was similar to that seen for the overall population (**Table T in S1 File**).

### Findings by age

Overall dietary consumption patterns were similar by age groups (**Tables F-J in S1 File**), except that younger adults (20–44 years) generally had higher consumption levels compared to other ages; legume, vitamin A, and vitamin D consumption was higher at older ages, and milk and fruit consumption were lower at older ages. Variability was evident in the relationship between the household estimates and individual intakes by age, especially among children (≤10 years) (**Fig 1, Tables F-J in S1 File**). For 0–5 year-olds, underestimation was seen for milk (-56%) with AME estimates, while milk was overestimated in all other ages (e.g., 114% in 20–44 year-olds); intakes of most other foods were overestimated to a greater extent in 0–5 year-olds compared with older children and adults. Notably, overestimation in children was substantially higher with the PC method that did not capture heterogeneity in validity of estimation of dietary factors by age (**Fig 1**). Variance explained by household estimates for each dietary factor by age was generally lower than that observed for the overall population; across age groups it was higher among younger children (0-5y) vs. all other ages (**Table U in S1 File**).

### Findings by education, religion and household income

Stratification by education revealed similar overall dietary consumption patterns, although adults with higher education (≥6 years) had generally higher intakes, especially for milk, cholesterol, meat/eggs, and fruits (**Tables K and L in S1 File**). The overestimation of AME estimates was modestly higher in individuals of higher vs. lower education (<6 years). PC estimates were quite similar by education, but diverged considerably from those of the overall population; notably for starchy vegetables (individuals with higher vs. lower education: -12%, -12%), total grains (-8%, -6%), and seafood (-7%, -9%) that were underestimated. Variance explained with the AME method tended to be higher among adults of lower vs. higher education (**Table V in S1 File**). The opposite was observed with the PC approach, but this was reversed with sex and age adjustment.

Intakes were only modestly higher among other religions (Christians, Hindus) vs. Muslims (**Tables M and N in S1 File**). The overestimation by both individualized household estimates was similar and generally higher in Muslims vs. other religions, except for seafood intakes (6%, 53%, respectively) (**Table W in S1 File**). Higher variance was explained in other religions vs. Muslims.

Consumption patterns were similar by household income, with higher intakes generally seen in the highest income group (**Tables O-S in S1 File**). Variability was unremarkable between household estimates and overestimation was higher for the lowest vs. all other income groups. Proportions of variance explained in 24hR intakes were generally modestly higher in the middle (2^nd^, 3^rd^ quintile) income groups (**Table X in S1 File**).

## Discussion

This investigation of household-level data and 24-hour dietary recall information from the same households in rural Bangladesh showed that individualized household estimates significantly exceeded individual intakes for nearly all dietary factors assessed, including by 12% for total energy, 0–242% for major food groups, 11%-30% for macronutrients and 13%-55% for micronutrients. The degree of overestimation varied by both sex and estimation method, with larger overestimation by AME in men and by PC in women; and also for young children (0–5 years), where milk intake was underestimated and intakes of other dietary factors were greatly overestimated. For all dietary factors, low to modest variation in intakes was explained by individualized household estimates, higher for the AME than the PC approach. These novel findings suggest that current methods to utilize household-level survey data are problematic for estimating individual dietary intakes.

Smallest to modest overestimation (<10–15%) was observed for key staple foods, including starchy vegetables, seafood, total grains, legumes and fats/oils; total energy; macronutrients; and specific vitamins and minerals, including niacin and zinc. This small to modest overestimation was similar between the AME and PC approach, and across all strata, except for 0–5 year-old children and individuals of low income in whom all dietary indicators were greatly overestimated. Interestingly, overestimation was higher among adults of higher vs. lower education, but given this population is mostly less educated, these results should be interpreted within that context. These findings suggest that established methods for estimating individual intakes from household surveys could be used to approximate specific dietary indicators, such as most frequently consumed foods, energy and macronutrients. Yet, intakes were still overestimated with further differences noted by individual characteristics and estimation method, and thus future applications of these methods should acknowledge and potentially try to account for this likely limitation.

In contrast, largest overestimation (≥20%) was seen for foods with high seasonal variation and increased variability between individuals, such as fruits and non-starchy vegetables; and less commonly consumed foods with fewer questions assessing their consumption in the household questionnaire, such as milk and meats/eggs. Almost all vitamins and minerals assessed were substantially overestimated, particularly those found in the above food sources, such as vitamin A, vitamin C, folate, and calcium. Overestimation was also larger for sodium consistent with increased variability in salt use -irrespectively of total energy intake levels- between individuals [64]. Similar findings were generally observed by individual characteristics (other than younger children) and estimation method. Our findings suggest that household estimates may not reasonably estimate dietary intakes of foods that may be under-represented in the household questionnaire, as well as foods and most vitamins and minerals that are generally characterized by high individual variability. These findings are consistent with methods used to generate such data, which were developed to capture household consumption rather than actual individual intake, and do not account for food wastage, food preparation alterations in weight and nutrient content, or food eaten away from home [65].

Notably, in really young (0–5 years) children all dietary factors were substantially overestimated, except for milk that was greatly underestimated. Household-level data are challenging for accurately estimating dietary intakes in infants and toddlers [34, 35, 40]. It is quite usual for children to leave a substantial proportion of leftover food [66], and this potential misconception between what is offered vs. what is consumed could contribute to an overall overestimation of household consumption. Furthermore, food consumption by different household members is not necessarily proportional to their energy requirements, particularly for young children and/or for specific foods. Our results showed that 24hR milk intake was highest in very young children compared to all other ages. Yet with the AME method, 0–5 year-olds were assigned the smallest proportion of the household milk consumption (relative to their energy requirements), while with the PC method they were treated as any other individual (assuming equal consumption). These findings confirm that household estimates are not appropriate for estimating dietary intakes of young children, whose dietary patterns vary from the rest of the population.

Both AME and PC approaches appear to be quite problematic for estimating individual intakes from household-level data, with key differences noted by individual characteristics. The AME improved estimation in women, children (0–5 and 6–10 years) and the PC in men, adolescents and adults, and individuals of low or high education, with no substantial differences in the overall population estimates or by religion or income. Women and children (≤10 years) in particular are two of the top interest population groups for several international organizations and priority guidelines [67–70]. These vulnerable populations are more likely to develop nutrient deficiencies, especially in LMICs [71], while there is increasing evidence [72–75] of obesity and NCD originating in early development stages and as a potential consequence of maternal and childhood malnutrition. Our results recognize the usefulness of the AME over the PC method for these populations, a finding consistent with the method used to derive PC estimates. The PC method is by design crude and assumes equal consumption within household members, thus limiting its ability to capture variations in individual intake. For all dietary factors variation explained by the AME estimates was consistently higher than the PC estimates, though it was still low to moderate. Potential improvements in the AME estimations -further dependent on data availability- could include the use of caloric equations that account for each individual’s anthropometrics and actual physical activity levels, to enable more accurate redistribution of household consumption.

Household surveys suggest several appropriate uses for its household consumption estimates, including constructing food balance sheets, providing food security indicators and poverty measurements, and informing food nutrition interventions [30]; yet, their use for assessing dietary quality or examining diet-disease burden relations [42, 76–78] can be problematic, further supported by the present findings. Given that several LMICs rely solely on household surveys for their food consumption estimates [38], these results highlight the need to further adapt existing individualization methods or develop new ones for better approximating individual-level intakes from household-level data. They also highlight the need to investigate the reasons behind observed overestimations, particularly for dietary factors and population groups with largest discrepancies.

The present findings support and greatly expand on prior reports which compared individualized household estimates to individual dietary data for energy and selected macro- and micronutrients such as protein, vitamin C, and iron, but not foods, or did not evaluate differences by individualization approach, and key population subgroups, such as sex, age, education, and income [34–36, 39–41, 79]. In Uganda, AME underestimated the energy adjusted intakes of key nutrients (e.g., vitamin C, folate, calcium) related to deficiencies in women (15-49y) and young children (2-5y) compared to 24hR estimates [34]. In Cameroon, AME estimates overestimated 24hR intakes for key foods assessed (vegetable oil, sugar, bouillon cube) in women (15-49y), and either over- (vegetable oil, bouillon cube) or under-(wheat flour, sugar) estimated intakes in children (1-5y) [35]. These studies used different surveys as sources for individual- and household-level data, not always comparable (i.e., both nationally representative); the sample was limited to households with only women and/or children of certain age; AME nutrient estimates were compared with energy-adjusted individual intakes; or only a few nutrients or single food items (for purposes of fortification) were assessed.

In prior analysis using the same BIHS survey, individual intakes from the 24hR were summed back to the household level rather than using actual household-level data [40]; subsequent application of AME approach efficiently redistributed energy, iron, zinc, vitamin A, and calcium among household members aged 4 years and above. In similar analyses -using computed household dietary data- in Ethiopia and Bangladesh, AME estimates compared well with individual intakes of energy, iron, and protein in adults and children, but not in women of reproductive age and infants (<2y) where substantial overestimation was seen [41]. These analyses do not test the validity of the AME approach in individualizing actual household data, which is particularly important when household questionnaires are the only source of dietary data.

Our investigation has several strengths. We systematically quantified differences between estimated individual dietary intake from household-level data and individual 24-hr recalls for multiple dietary indicators and different estimation methods, evaluating both rankings within the population, differences in means, and variation explained. We further assessed heterogeneity in this validity according to several key individual characteristics, and for a range of population subgroups, including children, women, and men. We included a wide range of nutrients related to both chronic diseases, and deficiencies, undernourishment, and child-maternal outcomes [10, 12, 13]. We adjusted our analyses for food wastage, as reported in the 24hR, and accounted for food consumed away in all estimates. To maximize comparability between diet assessment methods, we followed a series of standardized steps to harmonize description, classification, and quantification of food and nutrient intakes, including application of nutrient retention factors and yield factors, highlighting key crucial preparatory methodological steps in individualizing household consumption.

Despite comprehensive approaches to harmonize dietary data, there are inherent limitations in the dietary collection methods. We could not assess certain foods or nutrients, such as sugar-sweetened beverages, iodine, omega-3 and omega-6 fats. In this survey, as in other LMIC settings [38, 40, 41, 80], the main person responsible for cooking reported 24hR intakes for all other household members; though this is standard for children, it is generally not recommended for other adults, as it could lead to systematic reporting biases [81]. Only one 24hR was administered, which may have affected the accuracy of the estimated individual intakes. Yet, single 24hR can provide valid estimates of the absolute mean “usual” intake of a population subgroup, as assessed in the present analysis. Energy requirements for the AME approach were based on standard FAO equations that may be less sensitive in capturing individual variation. Seasonality (monga period in rural Bangladesh) was not covered [82], and our findings may differ for certain foods with substantial seasonal variation. Our results are based on a rural low-income population and their generalizability to urban or middle-income populations may be limited. Conversely, rural low-income populations globally are more likely to be lacking individual-level surveys and, thus, most relevant to assess in the present analysis. Future studies are needed to replicate the validity of and extend our approaches in different settings and populations over time.

In conclusion, household estimates substantially overestimated individual intakes in a national survey in rural Bangladesh with significant heterogeneity according to sex, age, education, and income. Methodology constructed in the present analysis showed that current methods for estimating individual intakes from household-level data are problematic, yet it confirmed usefulness of the AME vs. the PC approach in better approximating dietary intakes for key populations, mainly children and women. These findings will facilitate future use of household consumption estimates by scientists and policy makers to more accurately estimate dietary intakes, when household questionnaires are the only source of dietary data. Leveraging national household surveys already in place to routinely collect individual-level dietary data, even in a reasonably powered subset of the population, would be of great value to LMIC settings [83]. Relative to its importance as a global risk factor for health, disparities, and sustainability, national investment in the routine collection of individual-level dietary data should be prioritized for accurate diet assessment, burden analyses and policy implementation.

## Supporting information

S1 File Supplementary material. **Appendix A.** Dietary dataset preparation.
**Appendix B.** Methods for individualizing consumption data from household surveys.
**Table A.** Reliability and relevance assessment of the 2011–2012 BIHS by the International Household Survey Network criteria.
**Table B.** Definitions and units of dietary factors used in the 2011–2012 BIHS.
**Table C.** Relation between individualized household intake estimates as predictors of individual dietary intakes in the 2011–2012 BIHS.
**Table D.** Comparison of individualized household consumption and individual dietary intake estimates by dietary factor in men in the 2011–2012 BIHS.
**Table E.** Comparison of individualized household consumption and individual dietary intake estimates by dietary factor in women in the 2011–2012 BIHS.
**Table F.** Comparison of individualized household consumption and individual dietary intake estimates by dietary factor in children 5 years and under in the 2011–2012 BIHS.
**Table G.** Comparison of individualized household consumption and individual dietary intake estimates by dietary factor in children 6–10 years old in the 2011–2012 BIHS.
**Table H.** Comparison of individualized household consumption and individual dietary intake estimates by dietary factor in adolescents 11–19 years old in the 2011–2012 BIHS.
**Table I.** Comparison of individualized household consumption and individual dietary intake estimates by dietary factor in adults 20–44 years old in the 2011–2012 BIHS.
**Table J.** Comparison of individualized household consumption and individual dietary intake estimates by dietary factor in adults over 45 years of age in the 2011–2012 BIHS.
**Table K.** Comparison of individualized household consumption and individual dietary intake estimates by dietary factor in adults (≥20 years old) of low educational level (<6 years) in the 2011–2012 BIHS.
**Table L.** Comparison of individualized household consumption and individual dietary intake estimates by dietary factor in adults (≥20 years old) of medium and high educational level (≥6 years) in the 2011–2012 BIHS.
**Table M.** Comparison of individualized household consumption and individual dietary intake estimates by dietary factor in Muslims^1^ in the 2011–2012 BIHS.
**Table N.** Comparison of individualized household consumption and individual dietary intake estimates by dietary factor among other religions^1^ in the 2011–2012 BIHS.
**Table O.** Comparison of individualized household consumption and individual dietary intake estimates by dietary factor among individuals in the first quintile of household income in the 2011–2012 BIHS.
**Table P.** Comparison of individualized household consumption and individual dietary intake estimates by dietary factor among individuals in the second quintile of household income in the 2011–2012 BIHS.
**Table Q.** Comparison of individualized household consumption and individual dietary intake estimates by dietary factor among individuals in the third quintile of household income in the 2011–2012 BIHS.
**Table R.** Comparison of individualized household consumption and individual dietary intake estimates by dietary factor among individuals in the fourth quintile of household income in the 2011–2012 BIHS.
**Table S.** Comparison of individualized household consumption and individual dietary intake estimates by dietary factor among individuals in the fifth quintile of household income in the 2011–2012 BIHS.
**Table T.** Relation between individualized household intake estimates as predictors of individual dietary by sex in the 2011–2012 BIHS.
**Table U.** Relation between individualized household intake estimates as predictors of individual dietary intakes by age in the 2011–2012 BIHS.
**Table V.** Relation between individualized household intake estimates as predictors of individual dietary intakes by education in the 2011–2012 BIHS.
**Table W.** Relation between individualized household intake estimates as predictors of individual dietary intakes by religion in the 2011–2012 BIHS.
**Table X.** Relation between individualized household intake estimates as predictors of individual dietary intakes by household income in the 2011–2012 BIHS.
**Figure A.** Distribution of individualized household estimates and 24-hour recall intakes for selected dietary factors in the overall population in the 2011–2012 BIHS.
(DOCX)
