# Supplementary material for: Assessing dietary intakes from household budget surveys: A national analysis in Bangladesh
Source: PLoS One. 2018 Aug 27;13(8):e0202831. doi: 10.1371/journal.pone.0202831 (PMC6110494; doi:10.1371/journal.pone.0202831)
Supplement: S1 File — Appendix A. Dietary dataset preparation. Appendix B. Methods for individualizing consumption data from household surveys. Table A. Reliability and relevance assessment of the 2011–2012 BIHS by the International Household Survey Network criteria. Table B. Definitions and units of dietary factors used in the 2011–2012 BIHS. Table C. Relation between individualized household intake estimates as predictors of individual dietary intakes in the 2011–2012 BIHS. Table D. Comparison of individualized household consumption and individual dietary intake estimates by dietary factor in men in the 2011–2012 BIHS. Table E. Comparison of individualized household consumption and individual dietary intake estimates by dietary factor in women in the 2011–2012 BIHS. Table F. Comparison of individualized household consumption and individual dietary intake estimates by dietary factor in children 5 years and under in the 2011–2012 BIHS. Table G. Comparison of individualized household consumption and individual dietary intake estimates by dietary factor in children 6–10 years old in the 2011–2012 BIHS. Table H. Comparison of individualized household consumption and individual dietary intake estimates by dietary factor in adolescents 11–19 years old in the 2011–2012 BIHS. Table I. Comparison of individualized household consumption and individual dietary intake estimates by dietary factor in adults 20–44 years old in the 2011–2012 BIHS. Table J. Comparison of individualized household consumption and individual dietary intake estimates by dietary factor in adults over 45 years of age in the 2011–2012 BIHS. Table K. Comparison of individualized household consumption and individual dietary intake estimates by dietary factor in adults (≥20 years old) of low educational level (<6 years) in the 2011–2012 BIHS. Table L. Comparison of individualized household consumption and individual dietary intake estimates by dietary factor in adults (≥20 years old) of medium and high educational level (≥6 years [file pone.0202831.s001.docx]

**SUPPLEMENTARY MATERIAL
Assessing dietary intakes from household budget surveys: a national analysis in Bangladesh**

[**Appendix A.** Dietary dataset preparation. 3](#_Toc521925320)

[**Appendix B.** Methods for individualizing consumption data from household surveys. 6](#_Toc521925321)

[**Table A.** Reliability and relevance assessment of the 2011-2012 BIHS by the International Household Survey Network criteria. 9](#_Toc521925322)

[**Table B.** Definitions and units of dietary factors used in the 2011-2012 BIHS. 10](#_Toc521925323)

[**Table C.** Relation between individualized household intake estimates as predictors of individual dietary intakes in the 2011-2012 BIHS. 12](#_Toc521925324)

[**Table D.** Comparison of individualized household consumption and individual dietary intake estimates by dietary factor in men in the 2011-2012 BIHS. 15](#_Toc521925325)

[**Table E.** Comparison of individualized household consumption and individual dietary intake estimates by dietary factor in women in the 2011-2012 BIHS. 17](#_Toc521925326)

[**Table F.** Comparison of individualized household consumption and individual dietary intake estimates by dietary factor in children 5 years and under in the 2011-2012 BIHS. 19](#_Toc521925327)

[**Table G.** Comparison of individualized household consumption and individual dietary intake estimates by dietary factor in children 6-10 years old in the 2011-2012 BIHS. 21](#_Toc521925328)

[**Table H.** Comparison of individualized household consumption and individual dietary intake estimates by dietary factor in adolescents 11-19 years old in the 2011-2012 BIHS. 23](#_Toc521925329)

[**Table I.** Comparison of individualized household consumption and individual dietary intake estimates by dietary factor in adults 20-44 years old in the 2011-2012 BIHS. 25](#_Toc521925330)

[**Table J.** Comparison of individualized household consumption and individual dietary intake estimates by dietary factor in adults over 45 years of age in the 2011-2012 BIHS. 27](#_Toc521925331)

[**Table K.** Comparison of individualized household consumption and individual dietary intake estimates by dietary factor in adults (≥20 years old) of low educational level (<6 years) in the 2011-2012 BIHS. 29](#_Toc521925332)

[**Table L.** Comparison of individualized household consumption and individual dietary intake estimates by dietary factor in adults (≥20 years old) of medium and high educational level (≥6 years) in the 2011-2012 BIHS. 31](#_Toc521925333)

[**Table M.** Comparison of individualized household consumption and individual dietary intake estimates by dietary factor in Muslims^1^ in the 2011-2012 BIHS. 33](#_Toc521925334)

[**Table N.** Comparison of individualized household consumption and individual dietary intake estimates by dietary factor among other religions^1^ in the 2011-2012 BIHS. 35](#_Toc521925335)

[**Table O.** Comparison of individualized household consumption and individual dietary intake estimates by dietary factor among individuals in the first quintile of household income in the 2011-2012 BIHS. 37](#_Toc521925336)

[**Table P.** Comparison of individualized household consumption and individual dietary intake estimates by dietary factor among individuals in the second quintile of household income in the 2011-2012 BIHS. 39](#_Toc521925337)

[**Table Q.** Comparison of individualized household consumption and individual dietary intake estimates by dietary factor among individuals in the third quintile of household income in the 2011-2012 BIHS. 41](#_Toc521925338)

[**Table R.** Comparison of individualized household consumption and individual dietary intake estimates by dietary factor among individuals in the fourth quintile of household income in the 2011-2012 BIHS. 43](#_Toc521925339)

[**Table S.** Comparison of individualized household consumption and individual dietary intake estimates by dietary factor among individuals in the fifth quintile of household income in the 2011-2012 BIHS. 45](#_Toc521925340)

[**Table T.** Relation between individualized household intake estimates as predictors of individual dietary intakes by sex in the 2011-2012 BIHS. 47](#_Toc521925341)

[**Table U.** Relation between individualized household intake estimates as predictors of individual dietary intakes by age in the 2011-2012 BIHS. 50](#_Toc521925342)

[**Table V.** Relation between individualized household intake estimates as predictors of individual dietary intakes by education in the 2011-2012 BIHS. 54](#_Toc521925343)

[**Table W.** Relation between individualized household intake estimates as predictors of individual dietary intakes by religion in the 2011-2012 BIHS. 57](#_Toc521925344)

[**Table X.** Relation between individualized household intake estimates as predictors of individual dietary intakes by household income in the 2011-2012 BIHS. 60](#_Toc521925345)

[**Figure A.** Distribution of individualized household estimates and 24-hour recall intakes for selected dietary factors in the overall population in the 2011-2012 BIHS. 64](#_Toc521925346)

[**References** 66](#_Toc521925347)

# Appendix A. Dietary dataset preparation.

Dietary data were harmonized within and between the 24hR and household datasets. This process involved 7 key steps: (1) dataset retrieval, (2) unique food item identification and description, (3) food matching, (4)unit standardization, (5) food classification, (6) individualization of household consumption, and (7) final dataset preparation. Local experts provided advice on each of those steps, particularly for steps 2, 3 and 5.

**Step 1 – Dataset Retrieval**

The first step involved the identification of the BIHS datasets we would need. The relevant datasets included:

- Two 24hR datasets:
- One including the foods (single-ingredient, mixed dishes) consumed the previous day in the household and their total “as consumed” weight, along with the mixed dishes’ disaggregated ingredients and corresponding raw weights, and
- One including how much of these foods each household member consumed, stored as leftovers, were thrown away, given to guests, others, or livestock.
- One household consumption dataset. Foods consumed and their quantity for the household (raw weight for single-ingredient foods; cooked weight for mixed dishes).
- Five datasets containing the sociodemographic characteristics of interest.

**Step 2 - Unique food item identification and description**

We used the first dataset of the 24hR and the household consumption dataset to identify all unique food items (single-ingredient or disaggregated ingredient) reported in both of them (n=294). Seventy-seven food items were replaced by other reported ones, as suggested by local experts. Food description included the description of the unique food items (more than half had local names).

**Step 3 – Food matching**

We matched the unique food items to available food composition data based on the way they are commonly consumed (raw, boiled, fried, etc.).^1-3^ Food matching was realized on the food level; if nutrient composition was available for the overall recipe/mixed dish then that was preferred. The Bangladesh Food Composition Table^2^ and local Bangladeshi recipes were used as the primary sources (71.4% of foods) to find a “true” or similar match, and the USDA food composition database as a secondary one.^3^ If food composition data for the cooked state of a food was not available, we applied retention factors ^1,2,4-6^ to account for alterations in nutrient content during cooking.

**Step 4 – Unit standardization**

We applied correction factors to account for non-edible portions and cooking alterations in weight (yield factors).^2,4,7,8^ The process differed between the diet assessment methods. In 24hR there were three potential pathways; (i) single-ingredient foods consumed cooked – no action, use cooked weight as reported, (ii) single-ingredient foods consumed raw – correction factor for non-edible parts, if necessary, and (iii) mixed dish consumed cooked. In the latter case, we already had the cooked weight of the mixed dish overall, but only raw weight was available for the ingredients. Thus, we applied, where necessary, correction factors for non-edible portions and yield factors on the ingredients’ weights. In household consumption we followed the same logic for the first two pathways. For the third case, since mixed dishes were not disaggregated, we used standard recipes, provided by local experts, to estimate the contribution of each ingredient to the overall weight of the mixed dish. All quantities were converted and reported in standardized “as consumed” metrics, i.e., g/day for foods and macronutrients (other than cholesterol), and mg/day or μg/day for micronutrients.

**Step 5 – Food classification**

We classified unique food items (including disaggregated ingredients) to food groups (e.g., fruits, vegetables) using previously established methods;^9-11^ In the 24hR mixed dishes were already disaggregated, hence single-ingredient foods were classified into one food group, and mixed dishes were classified into at least one food group. In household consumption, simple foods were treated as in 24hR. Mixed dishes were not disaggregated; we used the standard recipes as in step 4. Foods were summed to correspond to the relevant food groups (e.g., apples, oranges, mangos etc. were summed to correspond to fruits).

**Step 6 – Estimation of individual dietary intakes based on household consumption**

Household food and nutrient consumption was individualized by the adult male equivalent (AME)^12^ and the per capita (PC)^13^ approach. See Appendix B for details on the estimation process.

**Step 7 – Final dataset preparation**

In final dataset preparation, we merged the 24hR datasets, by assigning and summing for each household member foods and respective nutrient content. Leftovers, and food given to guests, animals and others (recorded in the second 24hR dataset) were summed up by household to estimate the food wastage percentage. The 24hR and household consumption datasets were merged and matched by individual, excluding the individuals that had no available 24hR data. Sociodemographic data of interest were finally added, creating a complete dataset which included individual-level dietary and sociodemographic information.

# Appendix B. Methods for individualizing consumption data from household surveys.

**The Adult Male Equivalent approach**

The AME method^12^ assumes that household members do not receive an equal share of the food available for consumption, but rather the household food distribution is proportional to the individual’s share of the total household energy requirements. Essentially, with the AME, the energy requirements of household members of different age, sex, and condition (pregnant/ lactating women) are expressed as a multiple of an adult male’s energy requirements.

Two steps were involved in the calculation of AME as proposed by FAO;^12^ (1)estimation of energy requirements by age, sex, and condition (pregnant/lactating women), and (2) calculation of AME. Energy requirements were estimated using FAO’s Tables of daily energy requirements.^14^ Moderate physical activity was assumed, and for adults we assumed, in addition, a weight of 65 kg for men and a weight of 55 kg for women. The calculated AMEs for the BIHS analysis are presented in the Table B1 below. The process for individualizing household consumption based on the AME methodology is presented through the example that follows.

**Example.** A household of four individuals reports that the daily household consumption of bread is 1,650gr. Using the AME methodology, we will estimate the individualized consumption of bread.

**A)** AME calculation for each household member using the estimated energy requirements.

The AME is calculated as the ratio of the energy requirement of the household member to the energy requirement of an adult male, 18-30 years of age with moderate physical activity

**B)** Household AME calculation by summing the AMEs of the household members.

**C)** Individual AME calculation by dividing the AME by the household AME.

**D)** Individualized consumption of bread estimation by multiplying the individual AME with the household consumption of bread (1,650g).

| **Sex** | **Age (years)** | **Energy requirements (kcal/d)** | **AME** | **Household**  **AME** | **Individual AME**  **(AME ÷ Household AME)** | **Individualized consumption (g/d)** |
| --- | --- | --- | --- | --- | --- | --- |
| Woman | 48 | 2,375 | 0.778688525 | 3.508196721 | 0.221962617 | 366.2 |
| Man | 50 | 3,000 | 0.983606557 | 3.508196721 | 0.280373832 | 462.6 |
| Man | 19 | 3,050 | 1 | 3.508196721 | 0.285046729 | 470.3 |
| Woman | 12 | 2,275 | 0.745901639 | 3.508196721 | 0.212616822 | 350.8 |

**Table B1.** Calculated AMEs by sex, age, and pregnant/lactating status for the analysis of the 2011-2012 BIHS.

|  | **AME^1^** | | | |
| --- | --- | --- | --- | --- |
| **Age groups** | **Men** | **Women** | | |
|  |  | **Non-pregnant/ Non-lactating** | **Pregnant** | **Lactating** |
| 0-1 | 0.212923497 | 0.196857923 |  |  |
| 1-2 | 0.31147541 | 0.278688525 |  |  |
| 2-3 | 0.368852459 | 0.344262295 |  |  |
| 3-4 | 0.409836066 | 0.37704918 |  |  |
| 4-5 | 0.442622951 | 0.409836066 |  |  |
| 5-6 | 0.483606557 | 0.43442623 |  |  |
| 6-7 | 0.516393443 | 0.467213115 |  |  |
| 7-8 | 0.557377049 | 0.508196721 |  |  |
| 8-9 | 0.598360656 | 0.557377049 |  |  |
| 9-10 | 0.647540984 | 0.606557377 |  |  |
| 10-11 | 0.704918033 | 0.655737705 |  |  |
| 11-12 | 0.770491803 | 0.704918033 |  |  |
| 12-13 | 0.836065574 | 0.745901639 | 0.839344262 | 0.931967213 |
| 13-14 | 0.909836066 | 0.778688525 | 0.872131148 | 0.964754098 |
| 14-15 | 0.983606557 | 0.803278689 | 0.896721311 | 0.989344262 |
| 15-16 | 1.040983607 | 0.819672131 | 0.913114754 | 1.005737705 |
| 16-17 | 1.090163934 | 0.819672131 | 0.913114754 | 1.005737705 |
| 17-18 | 1.114754098 | 0.819672131 | 0.913114754 | 1.005737705 |
| 18-30 | 1 | 0.778688525 | 0.872131148 | 0.964754098 |
| 30-60 | 0.983606557 | 0.778688525 | 0.872131148 | 0.964754098 |
| 60-150 | 0.81147541 | 0.696721311 |  |  |

^1^ The AMEs were calculated based on FAO guidelines,^12^ Daily energy requirements were calculated based on FAO Tables for energy requirements, assuming moderate physical activity for individuals.^14^ For children under 1 year of age we used the average energy requirements of the 12 months (Table3.2), as the BIHS did not report the age of infants in months. For children and adolescents we used Tables 4.5 and 4.6. For adults we used Tables 5.4-5.9 using the midpoint of the physical activity level value ranges (Table 5.3) and the second interpolation method given on page 40, and we assumed a weight of 65 kilograms for men and a weight of 55 kilograms for women. In order to add the extra energy required (a) for pregnant women we assumed that they were in the second trimester of their pregnancy, and (b) for lactating women we used the average of extra energy required during first 6 month of exclusive breastfeeding and the next 6 months of partial breastfeeding.

**The per capita approach**

The per capita (PC) approach was the second method for estimating individual dietary intakes from household consumption; this method assumes that the available food in the household is equally distributed among household members. The PC household estimates were calculated by dividing the household consumption by the household size. Using the example for the AME calculation above, the individualized consumption based on the PC approach would be as presented in the Table.

| **Sex** | **Age (years)** | **Individualized consumption (g/d), AME** | **Individualized consumption (g/d), PC** |
| --- | --- | --- | --- |
| Woman | 48 | 366.2 | 412.5 |
| Man | 50 | 462.6 | 412.5 |
| Man | 19 | 470.3 | 412.5 |
| Woman | 12 | 350.8 | 412.5 |

# Table A. Reliability and relevance assessment of the 2011-2012 BIHS by the International Household Survey Network criteria.

| **Reliability Assessment - Areas of investigation^1^** | | | | | | | | | |
| --- | --- | --- | --- | --- | --- | --- | --- | --- | --- |
|  | **Recall period for at-home food data collection** | **Modes of food acquisition included** | **Completeness of enumeration** | **Comprehensiveness of the at-home food list.** | | **Specificity of the at-home food list.** | | **Quality of data collected on food consumed away from home** | **Accounting for seasonality.** |
| **IHSN**  **criteria** | Maximum 2 weeks | a) Market purchases  b) Food consumed from households’ own production  c) Food received in-kind (wages in kind, social transfers in kind, gifts) | Full accounting of all acquired food intended for consumption or all consumed food over the recall period. | a) All 14 Basic Food Groups (BFG) included**^2^**  b) At least 40% of food processed food items  c) The food list must include only foods and no other commodities | | a) A required minimum number of food items should be met for at least 10 of the 14 food groups  b) <5% of the food items (apart from prepared dishes) span more than one BFG | | a) Data are explicitly and deliberately  collected  b) less than or equal to two weeks  c) collect data for in-kind receipts | Survey conducted 2-4 a year, either for the same households or a new sample  OR  data collection is distributed  throughout a year |
| **BIHS characteristics** | 1 week | Modes of acquisition for consumption   - Purchases - Own production - Other sources | Consumption data are collected.  Error: Rule-out leading question on consumption | - 13/14 Basic Food Groups included (no alcoholic beverages) - Criterion met - Tobacco is included | | - It is met for 10/13 food groups - Criterion met | | - Criterion met - 1 week - Criterion met | - National sample collection period: December 15, 2011- March 15, 2012 (monga periods not covered) |
| **Relevance Assessment – Food data uses^3^** | | | | | | | | | |
| IHSN indicators | Quantities consumed of individual foods (direct use) | Quantities consumed of individual foods (direct use)  Calorie consumption and undernourishment  Calories consumed from individual foods/food groups  Protein & micronutrient consumption and insufficiencies  Dietary diversity  Percent of expenditures on food | | | Quantities consumed of individual foods  Calorie consumption and undernourishment | | Quantities consumed of individual foods (direct use)  Protein & micronutrient consumption and insufficiencies  Percent of households consuming individual foods  Percent of households purchasing individual foods | | |
| BIHS provides indicator | - Yes | - Yes | | | - Yes | | - Yes | | |

**^1^** “Reliability” is defined as the degree to which a survey collects data on the actual or “true” food consumption and/or expenditures of households in a country’s population. Assessing the reliability consists of assessing how the information is collected (i.e. whether the survey design and method complies with good practice), and it is based on seven areas of investigation.

**^2^** The 14 food groups include cereals, roots/tubers/plantains, pulses/nuts/seeds, vegetables, fruits, meat/poultry/offal, seafood, milk/milk products, eggs, oils/fats, sugar/jam/honey/chocolate/sweets, condiments/spices/baking agents, non-alcoholic beverages, alcoholic beverages

**^3^** The relevance of surveys for particular uses is assessed by investigating the following five methodological issues: a) measuring quantities of food consumed, b) calculation of calorie consumption, c) calculation of edible portions and the nutrient content of foods, d) calculation of per-capita indicators and nutrient insufficiencies and the importance of collecting data on the number of food partakers, and e) use of acquisition data to measure consumption. Twelve indicators are proposed depending on the category of use.

IHSN, International Household Survey Network; BIHS. Bangladesh Integrated Household Survey

# Table B. Definitions and units of dietary factors used in the 2011-2012 BIHS.

| **Dietary factor** | **Definition** | **Unit** | **Matched BIHS food items (n)** |
| --- | --- | --- | --- |
| Fruit | Total fruit intake, including fresh, frozen, cooked, canned, or dried fruit. This definition excludes fruit juices and salted or pickled fruits. | g/day | 33 |
| Non-starchy vegetables | Total vegetable intake, including fresh, frozen, cooked, canned, or dried vegetables. This definition excludes salted or pickled vegetables, vegetable juices, starchy vegetables (e.g., potatoes, corn, peas), and legumes. | g/day | 48 |
| Starchy vegetables^1^ | Total intake of white potatoes, including cooked (e.g. boiled, baked, mashed, fried), frozen, canned, dehydrated potatoes, french fries, chips, and crisps. Total intake of non-potato starchy vegetables (e.g. green peas, corn, yam, sweet potatoes), including fresh, frozen, cooked, canned, or dehydrated starchy vegetables. | g/day | 7 |
| Legumes | Total intake of beans and legumes (beans, lentils), including fresh, frozen, cooked, canned, or dried beans/legumes. This definition excludes peanuts and peanut butter. This definition includes soybeans but excludes soy milk and soy protein. | g/day | 7 |
| Total grains^2^ | Total intake of refined and whole grains. This definition excludes corn products including corn flour, corn meal, and popcorn. | g/day | 13 |
| Meat/ Eggs^3^ | Total intake of unprocessed and processed red meat and poultry, offal, and eggs produced by poultry/birds, including chicken, goose, or duck eggs. This definition excludes fish eggs. | g/day | 10 |
| Seafood | Total intake of fish and shellfish. Examples include salmon, tuna, trout, shrimp, crab, and oysters. | g/day | 38 |
| Milk, whole fat^4^ | Total whole-fat dairy milk intake. This definition excludes yogurt, fermented milk, and soy or other plant-derived milk (e.g. coconut milk, almond milk). | g/day | 3 |
| Fats/ Oils | Total intake of edible fats and oils, including butter, palm oil, olive oil, and soybean oil. | g/day | 4 |
| Energy | Total energy intake | kcal/day | NA |
| Protein | Total protein intake from all sources. | g/day | NA |
| Carbohydrates | Total carbohydrate intake | g/day | NA |
| Total fat | Total intake of total fat from all sources. | g/day | NA |
| Saturated fat | Total saturated fat intake from all. | g/day | NA |
| MUFA | Total monounsaturated fat intake from all sources. | g/day | NA |
| PUFA | Total poly-unsaturated fat intake from all sources. | g/day | NA |
| Cholesterol | Total dietary cholesterol from all sources. | mg/day | NA |
| Dietary fiber | Total dietary fiber intake from all sources (fruits, vegetables, grains, legumes, pulses). | g/day | NA |
| Vitamin A | Total intake of Vitamin A and provitamin A cartenoids from all sources, excluding dietary supplements. | μg RAE/day | NA |
| Vitamin D | Total intake of vitamin D from dietary sources only, including vitamin D2, vitamin D3, and Vitamin D provitamins and previtamins, excluding dietary supplements. | μg/day | NA |
| Vitamin E | Total intake of Vitamin E tocopherols and tocotrienols from all sources, excluding dietary supplements. | mg/day | NA |
| Thiamine | Total intake of thiamin from all sources, excluding dietary supplements. | mg/day | NA |
| Riboflavin | Total intake of vitamin B2 from all sources, excluding dietary supplements. | mg/day | NA |
| Niacin | Total intake of niacin from all sources, excluding dietary supplements. | mg/day | NA |
| Vitamin B6 | Total intake of vitamin B6 (including 2-methyl, 3-hydroxy, 5-hydroxymetrhyl pyridine derivatives that exhibit the nutritional activity of pyridoxine) from all sources, excluding dietary supplements. | mg/day | NA |
| Folate | Total intake of niacin from all sources, excluding dietary supplements. | μg/day | NA |
| Vitamin C | Total intake of vitamin C from all sources, excluding dietary supplements | mg/day | NA |
| Calcium | Total intake of calcium from all sources, excluding dietary supplements. | mg/day | NA |
| Iron | Total intake of heme and non-heme iron from all sources, excluding dietary supplements. | mg/day | NA |
| Sodium | Total intake of sodium from all sources. | mg/day | NA |
| Potassium | Total intake of potassium from all sources, excluding dietary supplements. | mg/day | NA |
| Magnesium | Total intake of magnesium from all sources, excluding dietary supplements. | mg/day | NA |
| Zinc | Total intake of zinc from all sources, excluding dietary supplements. | mg/day | NA |

^1^ Sum of the refined groups potatoes and other starchy vegetables due to very low other starchy vegetables intake.

^2^ Sum of the refined groups whole grains and refined grains due to very low whole grains intake.

^3^ Sum of the refined groups unprocessed red meats, processed meats, poultry, offal, and eggs due to very low intakes for all.

^4^ The BIHS household food consumption list included only whole-fat milk items.

BIHS, Bangladesh Integrated Household Survey

# Table C. Relation between individualized household intake estimates as predictors of individual dietary intakes in the 2011-2012 BIHS.

|  | **AME** | | | **PC** | | |
| --- | --- | --- | --- | --- | --- | --- |
| **Dietary Factor, unit** | **Intercept (SE)** | ***β* (SE)** | **R** | **Intercept (SE)** | ***β* (SE)** | **R** |
| **Food groups** |  |  |  |  |  |  |
| Fruits, g/d |  |  |  |  |  |  |
| Unadjusted | 5.4 (0.5) | 0.1 (0.02) | 0.06 | 3.7 (0.5) | 0.2 (0.02) | 0.06 |
| Sex and age | 7.0 (0.7) | 0.1 (0.02) | 0.06 | 3.9 (0.7) | 0.2 (0.02) | 0.06 |
| Multivariate | 10.7 (7.0) | 0.1 (0.02) | 0.07 | 7.2 (7.0) | 0.2 (0.02) | 0.06 |
| Non-starchy vegetables, g/d |  |  |  |  |  |  |
| Unadjusted | 29.4 (2.5) | 0.5 (0.01) | 0.15 | 96.6 (3.7) | 0.3 (0.01) | 0.09 |
| Sex and age | 23.8 (2.8) | 0.3 (0.01) | 0.19 | 8.0 (3.8) | 0.2 (0.01) | 0.18 |
| Multivariate | 14.4 (16.6) | 0.3 (0.01) | 0.20 | -9.8 (17.0) | 0.3 (0.01) | 0.19 |
| Starchy vegetables, g/d |  |  |  |  |  |  |
| Unadjusted | 17.1 (1.8) | 0.8 (0.02) | 0.15 | 56.3 (2.8) | 0.5 (0.03) | 0.09 |
| Sex and age | 13.5 (2.0) | 0.5 (0.03) | 0.18 | 1.2 (2.8) | 0.4 (0.03) | 0.16 |
| Multivariate | 15.5 (14.2) | 0.5 (0.03) | 0.18 | -1.9 (14.3) | 0.5 (0.03) | 0.17 |
| Legumes, g/d |  |  |  |  |  |  |
| Unadjusted | 6.1 (0.7) | 0.6 (0.04) | 0.13 | 7.8 (0.8) | 0.5 (0.04) | 0.12 |
| Sex and age | 2.4 (0.8) | 0.5 (0.04) | 0.13 | -2.3 (0.9) | 0.5 (0.04) | 0.13 |
| Multivariate | 9.7 (11.9) | 0.5 (0.04) | 0.13 | 3.9 (11.8) | 0.5 (0.04) | 0.13 |
| Total grains, g/d |  |  |  |  |  |  |
| Unadjusted | 86.9 (13.1) | 0.9 (0.01) | 0.48 | 626.3 (17.2) | 0.5 (0.01) | 0.12 |
| Sex and age | 137.1 (14.1) | 0.5 (0.01) | 0.57 | 77.2 (16.8) | 0.4 (0.01) | 0.53 |
| Multivariate | 207.4 (51.0) | 0.6 (0.01) | 0.60 | 42.2 (57.8) | 0.4 (0.01) | 0.55 |
| Meat/Eggs, g/d |  |  |  |  |  |  |
| Unadjusted | 4.7 (0.5) | 0.4 (0.03) | 0.13 | 3.9 (0.6) | 0.4 (0.04) | 0.12 |
| Sex and age | 5.7 (0.6) | 0.4 (0.03) | 0.13 | 1.7 (0.7) | 0.4 (0.04) | 0.12 |
| Multivariate | 9.3 (5.4) | 0.4 (0.03) | 0.15 | 4.9 (5.4) | 0.4 (0.04) | 0.14 |
| Seafood, g/d |  |  |  |  |  |  |
| Unadjusted | 6.8 (0.5) | 0.7 (0.02) | 0.22 | 11.0 (0.7) | 0.5 (0.03) | 0.17 |
| Sex and age | 3.8 (0.5) | 0.5 (0.02) | 0.23 | -1.8 (0.7) | 0.5 (0.03) | 0.22 |
| Multivariate | 3.2 (4.0) | 0.5 (0.02) | 0.23 | -3.9 (4.0) | 0.5 (0.03) | 0.22 |
| Milk, whole fat, g/d |  |  |  |  |  |  |
| Unadjusted | 6.4 (0.6) | 0.4 (0.02) | 0.24 | 1.4 (0.5) | 0.6 (0.02) | 0.29 |
| Sex and age | 24.9 (1.8) | 0.5 (0.02) | 0.26 | 15.9 (1.5) | 0.6 (0.02) | 0.30 |
| Multivariate | 21.5 (5.7) | 0.4 (0.02) | 0.26 | 14.1 (5.8) | 0.6 (0.02) | 0.30 |
| Fats/Oils, g/d |  |  |  |  |  |  |
| Unadjusted | 3.5 (0.3) | 0.7 (0.01) | 0.23 | 7.8 (0.5) | 0.5 (0.02) | 0.16 |
| Sex and age | 3.3 (0.3) | 0.5 (0.02) | 0.24 | -0.2 (0.5) | 0.5 (0.02) | 0.23 |
| Multivariate | 9.0 (2.2) | 0.5 (0.02) | 0.25 | 4.9 (2.2) | 0.5 (0.03) | 0.24 |
|  |  |  |  |  |  |  |
| **Energy & Macronutrients** |  |  |  |  |  |  |
| Energy, kcal/d |  |  |  |  |  |  |
| Unadjusted | 269.5 (15.8) | 0.8 (0.01) | 0.46 | 1052.8 (26.7) | 0.4 (0.01) | 0.13 |
| Sex and age | 413.2 (16.5) | 0.4 (0.01) | 0.55 | 308.4 (25.0) | 0.3 (0.01) | 0.51 |
| Multivariate | 498.6 (70.5) | 0.5 (0.01) | 0.57 | 237.0 (75.7) | 0.4 (0.01) | 0.53 |
| Protein, g/d |  |  |  |  |  |  |
| Unadjusted | 7.0 (0.4) | 0.8 (0.01) | 0.39 | 25.8 (0.7) | 0.4 (0.01) | 0.14 |
| Sex and age | 10.0 (0.4) | 0.4 (0.01) | 0.47 | 5.9 (0.7) | 0.3 (0.01) | 0.43 |
| Multivariate | 11.8 (2.4) | 0.5 (0.01) | 0.49 | 4.0 (2.5) | 0.4 (0.01) | 0.45 |
| Carbohydrates, g/d |  |  |  |  |  |  |
| Unadjusted | 44.8 (3.0) | 0.8 (0.01) | 0.46 | 204.1 (4.9) | 0.4 (0.01) | 0.12 |
| Sex and age | 71.4 (3.2) | 0.4 (0.01) | 0.55 | 56.9 (4.7) | 0.3 (0.01) | 0.51 |
| Multivariate | 80.3 (12.7) | 0.5 (0.01) | 0.58 | 35.3 (13.8) | 0.4 (0.01) | 0.54 |
| Total fat, g/d |  |  |  |  |  |  |
| Unadjusted | 7.0 (0.4) | 0.6 (0.01) | 0.27 | 10.9 (0.6) | 0.5 (0.02) | 0.20 |
| Sex and age | 7.3 (0.4) | 0.5 (0.02) | 0.29 | 1.5 (0.6) | 0.5 (0.02) | 0.27 |
| Multivariate | 13.0 (2.5) | 0.5 (0.02) | 0.29 | 6.1 (2.6) | 0.5 (0.02) | 0.28 |
| SFA, g/d |  |  |  |  |  |  |
| Unadjusted | 2.7 (0.2) | 0.4 (0.04) | 0.18 | 3.1 (0.2) | 0.4 (0.03) | 0.15 |
| Sex and age | 2.5 (0.2) | 0.3 (0.04) | 0.20 | 1.4 (0.2) | 0.3 (0.03) | 0.19 |
| Multivariate | 3.1 (0.5) | 0.3 (0.04) | 0.20 | 1.9 (0.5) | 0.3 (0.03) | 0.20 |
| MUFA, g/d |  |  |  |  |  |  |
| Unadjusted | 2.0 (0.1) | 0.6 (0.01) | 0.30 | 2.9 (0.2) | 0.5 (0.03) | 0.23 |
| Sex and age | 2.1 (0.1) | 0.5 (0.02) | 0.30 | 0.2 (0.2) | 0.5 (0.03) | 0.29 |
| Multivariate | 3.6 (0.7) | 0.5 (0.02) | 0.31 | 1.2 (0.7) | 0.5 (0.03) | 0.30 |
| PUFA, g/d |  |  |  |  |  |  |
| Unadjusted | 2.3 (0.2) | 0.7 (0.01) | 0.29 | 4.7 (0.3) | 0.6 (0.02) | 0.21 |
| Sex and age | 2.1 (0.2) | 0.6 (0.02) | 0.30 | -0.6 (0.3) | 0.5 (0.02) | 0.28 |
| Multivariate | 5.5 (1.4) | 0.6 (0.02) | 0.31 | 2.3 (1.4) | 0.5 (0.02) | 0.30 |
| Cholesterol, mg/d |  |  |  |  |  |  |
| Unadjusted | 17.0 (1.0) | 0.4 (0.03) | 0.14 | 13.1 (1.1) | 0.5 (0.03) | 0.14 |
| Sex and age | 19.9 (1.2) | 0.4 (0.03) | 0.14 | 8.5 (1.4) | 0.5 (0.03) | 0.14 |
| Multivariate | 21.3 (8.4) | 0.3 (0.03) | 0.15 | 8.9 (8.5) | 0.5 (0.03) | 0.15 |
| Fiber, g/d |  |  |  |  |  |  |
| Unadjusted | 2.9 (0.2) | 0.7 (0.01) | 0.38 | 14.7 (0.4) | 0.4 (0.01) | 0.11 |
| Sex and age | 4.6 (0.2) | 0.4 (0.01) | 0.48 | 3.9 (0.4) | 0.3 (0.01) | 0.45 |
| Multivariate | 5.0 (1.2) | 0.4 (0.01) | 0.50 | 2.5 (1.3) | 0.3 (0.01) | 0.47 |
|  |  |  |  |  |  |  |
| **Vitamins** |  |  |  |  |  |  |
| Vitamin A, μg RAE/d |  |  |  |  |  |  |
| Unadjusted | 64.3 (7.1) | 0.5 (0.03) | 0.11 | 98.4 (7.9) | 0.4 (0.03) | 0.10 |
| Sex and age | 37.0 (7.2) | 0.4 (0.03) | 0.12 | -5.3 (8.6) | 0.4 (0.03) | 0.11 |
| Multivariate | 17.1 (61.0) | 0.4 (0.03) | 0.12 | -36.3 (60.5) | 0.4 (0.03) | 0.11 |
| Vitamin D, μg/d |  |  |  |  |  |  |
| Unadjusted | 0.3 (0.01) | 0.6 (0.03) | 0.18 | 0.4 (0.03) | 0.6 (0.03) | 0.17 |
| Sex and age | 0.2 (0.04) | 0.5 (0.04) | 0.18 | -0.1 (0.04) | 0.6 (0.03) | 0.18 |
| Multivariate | 0.03 (0.3) | 0.5 (0.04) | 0.19 | -0.4 (0.3) | 0.6 (0.03) | 0.18 |
| Vitamin E, mg/d |  |  |  |  |  |  |
| Unadjusted | 0.8 (0.1) | 0.7 (0.01) | 0.32 | 1.7 (0.1) | 0.5 (0.02) | 0.23 |
| Sex and age | 0.7 (0.1) | 0.5 (0.01) | 0.33 | -0.3 (0.1) | 0.5 (0.02) | 0.31 |
| Multivariate | 1.5 (0.5) | 0.5 (0.02) | 0.34 | 0.3 (0.5) | 0.5 (0.02) | 0.32 |
| Thiamine, mg/d |  |  |  |  |  |  |
| Unadjusted | 0.1 (0.01) | 0.7 (0.01) | 0.34 | 0.4 (0.01) | 0.4 (0.01) | 0.12 |
| Sex and age | 0.2 (0.01) | 0.4 (0.01) | 0.42 | 0.1 (0.01) | 0.3 (0.01) | 0.39 |
| Multivariate | 0.2 (0.04) | 0.4 (0.01) | 0.43 | 0.1 (0.04) | 0.3 (0.01) | 0.40 |
| Riboflavin, mg/d |  |  |  |  |  |  |
| Unadjusted | 0.2 (0.01) | 0.5 (0.01) | 0.29 | 0.3 (0.01) | 0.4 (0.01) | 0.16 |
| Sex and age | 0.2 (0.01) | 0.3 (0.01) | 0.33 | 0.1 (0.01) | 0.3 (0.01) | 0.31 |
| Multivariate | 0.2 (0.02) | 0.3 (0.01) | 0.34 | 0.1 (0.02) | 0.4 (0.02) | 0.33 |
| Niacin, mg/d |  |  |  |  |  |  |
| Unadjusted | 1.1 (0.1) | 0.8 (0.01) | 0.50 | 5.8 (0.2) | 0.5 (0.01) | 0.19 |
| Sex and age | 1.5 (0.1) | 0.5 (0.01) | 0.56 | 0.1 (0.2) | 0.4 (0.01) | 0.51 |
| Multivariate | 1.8 (0.5) | 0.6 (0.01) | 0.58 | -0.8 (0.5) | 0.5 (0.01) | 0.53 |
| Vitamin B6, mg/d |  |  |  |  |  |  |
| Unadjusted | 0.1 (0.01) | 0.8 (0.01) | 0.49 | 0.4 (0.02) | 0.6 (0.01) | 0.28 |
| Sex and age | 0.1 (0.01) | 0.6 (0.01) | 0.52 | -0.1 (0.02) | 0.5 (0.01) | 0.46 |
| Multivariate | 0.1 (0.1) | 0.6 (0.01) | 0.55 | -0.2 (0.1) | 0.6 (0.01) | 0.50 |
| Folate, μg/d |  |  |  |  |  |  |
| Unadjusted | 26.9 (1.5) | 0.6 (0.01) | 0.20 | 70.5 (2.4) | 0.3 (0.02) | 0.10 |
| Sex and age | 30.4 (1.7) | 0.3 (0.01) | 0.24 | 18.8 (2.5) | 0.3 (0.02) | 0.23 |
| Multivariate | 32.8 (8.4) | 0.4 (0.02) | 0.25 | 14.2 (8.8) | 0.3 (0.02) | 0.24 |
| Vitamin C, mg/d |  |  |  |  |  |  |
| Unadjusted | 13.5 (0.9) | 0.4 (0.02) | 0.11 | 26 (1.1) | 0.2 (0.02) | 0.07 |
| Sex and age | 10.7 (0.9) | 0.3 (0.02) | 0.14 | 5.3 (1.2) | 0.2 (0.02) | 0.13 |
| Multivariate | 19.1 (5.4) | 0.3 (0.02) | 0.14 | 11.6 (5.5) | 0.2 (0.02) | 0.14 |
|  |  |  |  |  |  |  |
| **Minerals** |  |  |  |  |  |  |
| Calcium, mg/d |  |  |  |  |  |  |
| Unadjusted | 82.6 (4.7) | 0.5 (0.02) | 0.22 | 118.4 (7.6) | 0.5 (0.02) | 0.16 |
| Sex and age | 88.9 (5.3) | 0.4 (0.02) | 0.23 | 23.3 (7.9) | 0.4 (0.02) | 0.22 |
| Multivariate | 84.4 (30.8) | 0.4 (0.02) | 0.23 | -0.5 (31.0) | 0.4 (0.03) | 0.22 |
| Iron, mg/d |  |  |  |  |  |  |
| Unadjusted | 1.5 (0.1) | 0.7 (0.01) | 0.32 | 5.1 (0.2) | 0.4 (0.02) | 0.15 |
| Sex and age | 1.9 (0.1) | 0.4 (0.01) | 0.38 | 0.9 (0.2) | 0.3 (0.01) | 0.35 |
| Multivariate | 1.8 (0.6) | 0.4 (0.01) | 0.39 | 0.1 (0.6) | 0.4 (0.02) | 0.37 |
| Sodium, mg/d |  |  |  |  |  |  |
| Unadjusted | 1472.9 (53.7) | 0.5 (0.01) | 0.08 | 3284.4 (90.3) | 0.2 (0.02) | 0.02 |
| Sex and age | 1530.3 (55.6) | 0.2 (0.01) | 0.14 | 1361.0 (91.0) | 0.1 (0.02) | 0.13 |
| Multivariate | 2794.9 (570.6) | 0.2 (0.01) | 0.14 | 2574.0 (579.3) | 0.1 (0.02) | 0.14 |
| Potassium, mg/d |  |  |  |  |  |  |
| Unadjusted | 284.6 (13.0) | 0.6 (0.01) | 0.33 | 833.1 (20.0) | 0.3 (0.01) | 0.12 |
| Sex and age | 359.7 (12.9) | 0.3 (0.01) | 0.42 | 267.8 (19.5) | 0.3 (0.01) | 0.39 |
| Multivariate | 362.5 (66.2) | 0.4 (0.01) | 0.43 | 199.4 (68.3) | 0.3 (0.01) | 0.41 |
| Magnesium, mg/d |  |  |  |  |  |  |
| Unadjusted | 37.4 (2.7) | 0.7 (0.01) | 0.41 | 177.3 (4.4) | 0.4 (0.01) | 0.11 |
| Sex and age | 59.8 (2.8) | 0.4 (0.01) | 0.52 | 50.5 (4.2) | 0.3 (0.01) | 0.48 |
| Multivariate | 62.9 (12.1) | 0.4 (0.01) | 0.54 | 31.6 (12.8) | 0.3 (0.01) | 0.50 |
| Zinc, mg/d |  |  |  |  |  |  |
| Unadjusted | 1.1 (0.1) | 0.8 (0.01) | 0.43 | 4.6 (0.1) | 0.4 (0.01) | 0.12 |
| Sex and age | 1.7 (0.1) | 0.4 (0.01) | 0.52 | 1.3 (0.1) | 0.3 (0.01) | 0.49 |
| Multivariate | 1.9 (0.3) | 0.5 (0.01) | 0.54 | 0.9 (0.4) | 0.3 (0.01) | 0.51 |

All p-values are <0.001.

On the basis of linear regression models with 24-hour diet recall (24hR) intakes as the dependent variable and individualized Adult Male Equivalent (AME) or per capita (PC) consumption estimates from household questionnaire as the independent variable. The sex and age model was categorized as follows: age, ≤5, 6-10, 11-19, 20-44, and ≥45 years; sex, men and women. The multivariate model was adjusted for the following covariates: age (≤5, 6-10, 11-19, 20-44, and ≥45 years), sex (men, women), education (<6 years of education, ≥6 years of education), religion (Muslims, other), household income (quintiles), respondent’s age (continuous), sex (men, women) and education (<6 years of education, ≥6 years of education), household size, number of children within household, and food wastage percentage (using 24hR data, we calculated for each household, the percent of food wastage -sum of food waste, and food given to guests, others and animals- to total consumed food (mean: 11.6%, SD: 13.6)). *Β* (HH AME) represents the change in the individual intake (24hR) for every unit increase in the HH AME mean. SEs for the intercept and *β* (ΗΗ ΑΜΕ) are presented. R^2^ represents the coefficient of determination for the overall model.

BIHS, Bangladesh Integrated Household Survey; MUFA, Monounsaturated fats; PUFA, Polyunsaturated fats; SFA, Saturated fat

# Table D. Comparison of individualized household consumption and individual dietary intake estimates by dietary factor in men in the 2011-2012 BIHS.

| **Dietary Factor, unit^1^** | **Observations (n)^2^** | **Consumption**  **(mean, SD)^3^** | | | **Difference between 24hR and AME (mean, SD)^4^** | | **Difference between 24hR and PC (mean, SD)^4^** | |
| --- | --- | --- | --- | --- | --- | --- | --- | --- |
|  |  | **AME^3^** | **PC^3^** | **24hR^3^** | **Absolute^5^** | **Percent** | **Absolute^6^** | **Percent** |
| **Food groups** |  |  |  |  |  |  |  |  |
| Fruits, g/d | 10,488 | 32.0 (54.6) | 30.1 (49.2) | 9.2 (36.8) | 22.7 (58.2) | 248 | 20.8 (53.7) | 226 |
| Non-starchy vegetables, g/d | 10,502 | 276.7 (177.1) | 258.9 (150.5) | 174.9 (157.2) | 101.8 (183.8) | 58 | 84.0 (182.1) | 48 |
| Starchy vegetables, g/d | 10,382 | 112.2 (73.1) | 105.1 (62.1) | 109.7 (103.4) | 2.6 (99.1) | 2 | -4.6 (102.5) | -4 |
| Legumes, g/d | 9,367 | 23.9 (33.7) | 22.5 (30.5) | 21.3 (53.1) | 2.6 (51.8) | 12 | 1.1 (51.5) | 5 |
| Total grains, g/d | 6,289 | 1580.9 (578.5) | 1478.2 (399.3) | 1436.1 (615.4) | 144.8 (451.2) | 10 | 42.1 (593.8) | 3 |
| Meat/Eggs, g/d | 7.070 | 18.1 (21.3) | 16.9 (19.1) | 12.9 (27.7) | 5.2 (28.1) | 40 | 4.0 (27.5) | 31 |
| Seafood, g/d | 10,502 | 27.4 (26.2) | 25.6 (23.0) | 25.8 (31.3) | 1.6 (29.8) | 6 | -0.2 (30.1) | -1 |
| Milk, whole fat, g/d | 10,013 | 28.2 (57.8) | 26.5 (52.1) | 20.1 (62.5) | 8.2 (58.7) | 41 | 6.4 (53.9) | 32 |
| Fats/Oils, g/d | 10,499 | 21.3 (13.8) | 19.9 (11.8) | 18.6 (16.5) | 2.7 (15.5) | 15 | 1.3 (15.9) | 7 |
|  |  |  |  |  |  |  |  |  |
| **Energy & Macronutrients** |  |  |  |  |  |  |  |  |
| Energy, kcal/d | 10,502 | 2480.5 (927.3) | 2319.5 (646.1) | 2175.3 (888.9) | 305.1 (705.0) | 14 | 144.1 (888.0) | 7 |
| Protein, g/d | 10,502 | 60.3 (25.0) | 56.4 (18.6) | 52.7 (24.0) | 7.6 (20.6) | 14 | 3.7 (24.1) | 7 |
| Carbohydrates, g/d | 10,502 | 475.3 (175.8) | 444.4 (121.0) | 418.9 (173.0) | 56.5 (135.1) | 13 | 25.5 (172.6) | 6 |
| Total fat, g/d | 10,502 | 31.6 (19.3) | 29.6 (16.3) | 27.0 (20.6) | 4.5 (19.1) | 17 | 2.5 (19.5) | 9 |
| SFA, g/d | 10,502 | 6.9 (5.5) | 6.5 (4.7) | 5.7 (5.1) | 1.2 (5.6) | 21 | 0.8 (5.3) | 14 |
| MUFA, g/d | 10,502 | 9.6 (5.9) | 9.0 (5.0) | 8.2 (6.4) | 1.4 (5.8) | 17 | 0.8 (5.9) | 10 |
| PUFA, g/d | 10,502 | 14.4 (9.3) | 13.5 (8.0) | 12.7 (10.6) | 1.7 (9.5) | 13 | 0.7 (9.9) | 6 |
| Cholesterol, mg/d | 10,502 | 45.7 (48.5) | 42.7 (43.4) | 36.1 (62.2) | 9.6 (62.1) | 27 | 6.7 (61.1) | 19 |
| Fiber, g/d | 10,502 | 33.5 (13.4) | 31.3 (9.8) | 27.3 (12.4) | 6.1 (11.0) | 22 | 4.0 (12.9) | 15 |
|  |  |  |  |  |  |  |  |  |
| **Vitamins** |  |  |  |  |  |  |  |  |
| Vitamin A, μg RAE/d | 10,502 | 339.9 (371.2) | 318.6 (332.0) | 219.8 (415.8) | 120.1 (450.7) | 55 | 98.9 (442.5) | 45 |
| Vitamin D, μg/d | 10,502 | 1.4 (2.0) | 1.3 (1.8) | 1.2 (2.8) | 0.2 (2.6) | 17 | 0.1 (2.6) | 8 |
| Vitamin E, mg/d | 10,502 | 5.6 (3.3) | 5.3 (2.8) | 4.7 (3.5) | 1.0 (3.1) | 21 | 0.6 (3.3) | 13 |
| Thiamine, mg/d | 10,502 | 1.0 (0.4) | 0.9 (0.3) | 0.8 (0.4) | 0.2 (0.4) | 25 | 0.1 (0.4) | 13 |
| Riboflavin, mg/d | 10,502 | 0.6 (0.3) | 0.6 (0.2) | 0.5 (0.3) | 0.1 (0.3) | 20 | 0.1 (0.3) | 20 |
| Niacin, mg/d | 10,502 | 17.1 (6.8) | 16.0 (5.0) | 14.8 (6.7) | 2.3 (5.0) | 16 | 1.2 (6.3) | 8 |
| Vitamin B6, mg/d | 10,502 | 1.5 (0.7) | 1.4 (0.6) | 1.2 (0.7) | 0.2 (0.5) | 17 | 0.1 (0.6) | 8 |
| Folate, μg/d | 10,502 | 167.2 (86.6) | 156.4 (70.2) | 126.3 (80.9) | 40.9 (86.7) | 32 | 30.1 (88.6) | 24 |
| Vitamin C, mg/d | 10,502 | 69.4 (47.9) | 65.0 (41.0) | 43.8 (42.8) | 25.5 (52.3) | 58 | 21.1 (50.8) | 48 |
|  |  |  |  |  |  |  |  |  |
| **Minerals** |  |  |  |  |  |  |  |  |
| Calcium, mg/d | 10,502 | 365.5 (219.5) | 341.8 (183.6) | 290.1 (238.2) | 75.4 (231.5) | 26 | 51.7 (232.5) | 18 |
| Iron, mg/d | 10,502 | 12.7 (6.0) | 11.9 (4.7) | 10.4 (5.7) | 2.3 (5.4) | 22 | 1.5 (5.8) | 14 |
| Sodium, mg/d | 10,502 | 6207.5 (3283.2) | 5814.6 (2693.1) | 4368.5 (2960.4) | 1839.0 (3660.8) | 42 | 1446.1 (3660.5) | 33 |
| Potassium, mg/d | 10,502 | 1856.2 (822.2) | 1737.0 (634.4) | 1467.2 (689.4) | 389.1 (695.5) | 27 | 269.9 (757.0) | 18 |
| Magnesium, mg/d | 10,502 | 402.7 (156.1) | 376.6 (111.5) | 336.9 (144.4) | 65.9 (123.3) | 20 | 39.8 (149.2) | 12 |
| Zinc, mg/d | 10,502 | 10.5 (4.0) | 9.8 (2.8) | 9.0 (3.8) | 1.4 (3.2) | 16 | 0.7 (3.9) | 8 |

^1^ Dietary factors presented had adequate data/information for the present analysis.

^2^ Sample sizes differ because we performed paired analysis for each dietary factor, i.e., for each analysis we used only the individuals with available intake data for both diet assessments.

^3^ Bangladesh Integrated Household Survey (BIHS) 2011-2012 provided household-level dietary data from a 7-day household consumption questionnaire and individual-level data from 24-hour recalls (24hR). Household consumption was individualized by applying a) the Adult Male Equivalent (AME) method,^12^ as proposed by FAO^14^, assuming moderate physical activity, and b) the per capita (PC) approach assuming equal distribution among household members (Appendix B). Individual intake was estimated from 24hR.

^4^ Absolute differences correspond to AME-24hR and PC-24hR respectively, and percentage differences correspond to absolute difference/24hR*100.

^5^ Differences between means were significant for all dietary factors (paired t-test, P<0.01).

^6^ Differences between means were significant for all dietary factors (paired t-test, P<0.05), with the exception of seafood (p=0.53).

MUFA, Monounsaturated fats; PUFA, Polyunsaturated fats; SFA, Saturated fat

# Table E. Comparison of individualized household consumption and individual dietary intake estimates by dietary factor in women in the 2011-2012 BIHS.

| **Dietary Factor, unit^1^** | **Observations (n)^2^** | **Consumption**  **(mean, SD)^3^** | | | **Difference between 24hR and AME (mean, SD)^4^** | | **Difference between 24hR and PC (mean, SD)^4^** | |
| --- | --- | --- | --- | --- | --- | --- | --- | --- |
|  |  | **AME^3^** | **PC^3^** | **24hR^3^** | **Absolute^5^** | **Percent** | **Absolute^6^** | **Percent** |
| **Food groups** |  |  |  |  |  |  |  |  |
| Fruits, g/d | 11,658 | 29.7 (51.7) | 31.0 (51.6) | 8.7 (34.7) | 21.0 (54.4) | 241 | 22.2 (54.3) | 255 |
| Non-starchy vegetables, g/d | 11,671 | 245.5 (155.4) | 257.9 (150.6) | 160.6 (137.2) | 84.9 (164.5) | 53 | 97.3 (171.3) | 61 |
| Starchy vegetables, g/d | 11,537 | 99.6 (65.3) | 104.5 (63.1) | 100.2 (92.3) | 0.6 (91.8) | 1 | 4.4 (95.4) | 4 |
| Legumes, g/d | 10,518 | 21.1 (31.0) | 22.1 (31.2) | 19.0 (47.4) | 2.1 (46.2) | 11 | 3.1 (46.8) | 16 |
| Total grains, g/d | 7,021 | 1377.7 (476.3) | 1450.1 (399.9) | 1305.2 (505.2) | 72.2 (407.7) | 6 | 144.6 (525.5) | 11 |
| Meat/Eggs, g/d | 7,949 | 16.4 (19.8) | 17.2 (19.8) | 10.9 (25.0) | 5.5 (25.9) | 50 | 6.3 (26.0) | 58 |
| Seafood, g/d | 11,671 | 24.1 (23.2) | 25.3 (23.2) | 23.5 (28.6) | 0.6 (27.4) | 3 | 1.8 (28.3) | 8 |
| Milk, whole fat, g/d | 11,188 | 24.7 (50.5) | 25.9 (51.8) | 15.7 (58.9) | 9.0 (57.7) | 57 | 10.3 (55.5) | 66 |
| Fats/Oils, g/d | 11,663 | 19.3 (12.6) | 20.2 (12.1) | 17.1 (15.6) | 2.2 (14.9) | 13 | 3.1 (15.4) | 18 |
|  |  |  |  |  |  |  |  |  |
| **Energy & Macronutrients** |  |  |  |  |  |  |  |  |
| Energy, kcal/d | 11,671 | 2180.2 (790.8) | 2292.7 (661.6) | 1965.0 (736.0) | 215.2 (652.8) | 11 | 327.7 (798.5) | 17 |
| Protein, g/d | 11,671 | 53.1 (21.8) | 55.8 (19.1) | 47.5 (20.3) | 5.6 (19.1) | 12 | 8.3 (22.1) | 17 |
| Carbohydrates, g/d | 11,671 | 415.9 (147.5) | 437.6 (122.7) | 378.7 (141.5) | 37.2 (123.3) | 10 | 58.9 (153.8) | 16 |
| Total fat, g/d | 11,671 | 28.5 (17.9) | 29.9 (17.1) | 24.3 (18.8) | 4.2 (18.3) | 17 | 5.6 (18.9) | 23 |
| SFA, g/d | 11,671 | 6.2 (5.4) | 6.5 (5.3) | 5.0 (4.5) | 1.2 (5.4) | 24 | 1.5 (5.5) | 30 |
| MUFA, g/d | 11,671 | 8.6 (5.2) | 9.0 (5.0) | 7.3 (5.6) | 1.3 (5.3) | 18 | 1.7 (5.5) | 23 |
| PUFA, g/d | 11,671 | 13.1 (8.6) | 13.8 (8.2) | 11.7 (10.0) | 1.7 (9.5) | 15 | 0.7 (9.9) | 6 |
| Cholesterol, mg/d | 11,670 | 41.3 (44.8) | 43.3 (44.5) | 31.0 (55.4) | 10.2 (57.4) | 33 | 12.2 (56.8) | 39 |
| Fiber, g/d | 11,671 | 29.5 (11.6) | 31.0 (10.1) | 24.8 (10.3) | 4.7 (10.2) | 19 | 6.2 (11.9) | 25 |
|  |  |  |  |  |  |  |  |  |
| **Vitamins** |  |  |  |  |  |  |  |  |
| Vitamin A, μg RAE/d | 11,671 | 307.4 (341.5) | 322.1 (341.3) | 210.4 (394.6) | 97.0 (428.6) | 46 | 111.7 (435.1) | 53 |
| Vitamin D, μg/d | 11,671 | 1.2 (1.8) | 1.3 (1.8) | 1.1 (2.6) | 0.2 (2.5) | 18 | 0.2 (2.5) | 18 |
| Vitamin E, mg/d | 11,671 | 5.1 (3.1) | 5.4 (2.9) | 4.3 (3.3) | 0.8 (3.0) | 19 | 1.0 (3.2) | 23 |
| Thiamine, mg/d | 11,671 | 0.9 (0.4) | 0.9 (0.3) | 0.7 (0.3) | 0.1 (0.3) | 14 | 0.2 (0.4) | 29 |
| Riboflavin, mg/d | 11,671 | 0.5 (0.3) | 0.6 (0.2) | 0.4 (0.2) | 0.1 (0.3) | 25 | 0.1 (0.3) | 25 |
| Niacin, mg/d | 11,671 | 15.0 (5.8) | 15.7 (5.1) | 13.4 (5.6) | 1.5 (4.6) | 11 | 2.3 (5.7) | 17 |
| Vitamin B6, mg/d | 11,671 | 1.3 (0.6) | 1.4 (0.6) | 1.1 (0.6) | 0.2 (0.5) | 18 | 0.2 (0.6) | 18 |
| Folate, μg/d | 11,671 | 148.3 (76.7) | 155.8 (71.6) | 115.2 (73.3) | 33.1 (81.3) | 29 | 40.6 (85.5) | 35 |
| Vitamin C, mg/d | 11,671 | 61.9 (42.6) | 65.0 (41.5) | 40.6 (38.6) | 21.3 (47.2) | 52 | 24.4 (48.6) | 60 |
|  |  |  |  |  |  |  |  |  |
| **Minerals** |  |  |  |  |  |  |  |  |
| Calcium, mg/d | 11,671 | 323.6 (194.5) | 340.0 (186.5) | 259.9 (206.7) | 63.7 (213.4) | 25 | 80.1 (218.0) | 31 |
| Iron, mg/d | 11,671 | 11.3 (5.3) | 11.8 (4.8) | 9.4 (4.8) | 1.9 (4.8) | 20 | 2.5 (5.3) | 27 |
| Sodium, mg/d | 11,671 | 5538.1 (2981.6) | 5811.4 (2780.4) | 4095.8 (3166.7) | 1442.4 (3794.6) | 35 | 1715.6 (3900.1) | 42 |
| Potassium, mg/d | 11,671 | 1646.0 (734.5) | 1728.5 (658.8) | 1329.3 (582.0) | 316.7 (644.4) | 24 | 399.3 (709.7) | 30 |
| Magnesium, mg/d | 11,671 | 353.7 (133.6) | 371.9 (113.9) | 306.0 (120.7) | 47.7 (114.2) | 16 | 65.9 (136.6) | 22 |
| Zinc, mg/d | 11,671 | 9.2 (3.4) | 9.7 (2.9) | 8.2 (3.2) | 1.0 (2.9) | 12 | 1.5 (3.5) | 18 |

^1^ Dietary factors presented had adequate data/information for the present analysis.

^2^ Sample sizes differ because we performed paired analysis for each dietary factor, i.e., for each analysis we used only the individuals with available intake data for both diet assessments.

^3^ Bangladesh Integrated Household Survey (BIHS) 2011-2012 provided household-level dietary data from a 7-day household consumption questionnaire and individual-level data from 24-hour recalls (24hR). Household consumption was individualized by applying a) the Adult Male Equivalent (AME) method,^12^ as proposed by FAO^14^, assuming moderate physical activity, and b) the per capita (PC) approach assuming equal distribution among household members (Appendix B). Individual intake was estimated from 24hR.

^4^ Absolute differences correspond to AME-24hR and PC-24hR respectively, and percentage differences correspond to absolute difference/24hR*100.

^5^ Differences between means were significant for all dietary factors (paired t-test, P<0.05), with the exception of starchy vegetables (P=0.51).

^6^ Differences between means were significant for all dietary factors (paired t-test, P<0.0001).

MUFA, Monounsaturated fats; PUFA, Polyunsaturated fats; SFA, Saturated fat

# Table F. Comparison of individualized household consumption and individual dietary intake estimates by dietary factor in children 5 years and under in the 2011-2012 BIHS.

| **Dietary Factor, unit^1^** | **Observations (n)^2^** | **Consumption**  **(mean, SD)^3^** | | | **Difference between 24hR and AME (mean, SD)^4^** | | **Difference between 24hR and PC (mean, SD)^4^** | |
| --- | --- | --- | --- | --- | --- | --- | --- | --- |
|  |  | **AME ^3^** | **PC ^3^** | **24hR ^3^** | **Absolute^5^** | **Percent** | **Absolute^6^** | **Percent** |
| **Food groups** |  |  |  |  |  |  |  |  |
| Fruits, g/d | 2,806 | 16.1 (28.6) | 29.2 (49.4) | 8.7 (31.0) | 7.3 (35.5) | 84 | 20.5 (50.4) | 236 |
| Non-starchy vegetables, g/d | 2,807 | 126.1 (81.9) | 230.2 (130.1) | 62.3 (76.8) | 63.8 (92.6) | 102 | 167.9 (136.5) | 270 |
| Starchy vegetables, g/d | 2,770 | 52.2 (33.1) | 95.1 (53.7) | 42.1 (50.5) | 10.1 (50.7) | 24 | 53.0 (65.6) | 126 |
| Legumes, g/d | 2,511 | 10.4 (15.2) | 19.0 (26.3) | 8.8 (27.0) | 1.6 (25.7) | 18 | 10.2 (30.9) | 116 |
| Total grains, g/d | 1,587 | 701.0 (246.7) | 1300.3 (348.9) | 499.3 (333.9) | 201.6 (265.8) | 40 | 801.0 (431.5) | 160 |
| Meat/Eggs, g/d | 1,764 | 9.0 (10.3) | 16.5 (17.4) | 9.2 (20.1) | -0.2 (19.0) | -2 | 7.3 (21.8) | 79 |
| Seafood, g/d | 2,807 | 12.7 (11.9) | 23.2 (20.2) | 10.0 (15.7) | 2.7 (15.6) | 27 | 13.2 (21.4) | 132 |
| Milk, whole fat, g/d | 2,705 | 12.6 (24.8) | 23.8 (45.6) | 28.8 (85.7) | -16.2 (75.5) | -56 | -5.1 (69.2) | -18 |
| Fats/Oils, g/d | 2,807 | 9.8 (6.4) | 17.9 (10.4) | 7.9 (8.7) | 1.9 (8.1) | 24 | 10.1 (11.1) | 128 |
|  |  |  |  |  |  |  |  |  |
| **Energy & Macronutrients** |  |  |  |  |  |  |  |  |
| Energy, kcal/d | 2,807 | 1129.6 (403.3) | 2068.1 (557.2) | 880.0 (507.0) | 249.6 (426.4) | 28 | 1188.1 (657.9) | 135 |
| Protein, g/d | 2,807 | 27.5 (11.0) | 50.3 (15.9) | 21.8 (13.8) | 5.7 (12.1) | 26 | 28.5 (18.0) | 131 |
| Carbohydrates, g/d | 2,807 | 216.0 (76.0) | 395.6 (105.6) | 164.2 (97.4) | 51.8 (82.0) | 32 | 231.4 (127.3) | 141 |
| Total fat, g/d | 2,807 | 14.5 (9.2) | 26.6 (14.9) | 13.2 (12.0) | 1.3 (10.9) | 10 | 13.4 (14.9) | 102 |
| SFA, g/d | 2,807 | 3.2 (3.0) | 5.8 (5.0) | 3.1 (3.5) | 0.1 (3.6) | 3 | 2.7 (5.0) | 87 |
| MUFA, g/d | 2,807 | 4.4 (2.5) | 8.1 (4.1) | 4.2 (4.1) | 0.3 (3.7) | 7 | 3.9 (4.6) | 93 |
| PUFA, g/d | 2,807 | 6.7 (4.4) | 12.2 (7.2) | 5.5 (5.4) | 1.2 (4.9) | 22 | 6.7 (7.0) | 122 |
| Cholesterol, mg/d | 2,807 | 21.4 (22.7) | 39.3 (38.9) | 25.2 (54.8) | -3.8 (51.6) | -15 | 14.1 (54.8) | 56 |
| Fiber, g/d | 2,807 | 15.2 (5.9) | 27.9 (8.4) | 10.4 (6.9) | 4.8 (6.3) | 46 | 17.9 (9.6) | 172 |
|  |  |  |  |  |  |  |  |  |
| **Vitamins** |  |  |  |  |  |  |  |  |
| Vitamin A, μg RAE/d | 2,807 | 158.3 (181.7) | 288.1 (302.8) | 107.7 (219.1) | 50.6 (247.3) | 47 | 180.4 (329.4) | 168 |
| Vitamin D, μg/d | 2,807 | 0.6 (1.0) | 1.2 (1.7) | 0.5 (1.3) | 0.2 (1.3) | 40 | 0.7 (1.7) | 140 |
| Vitamin E, mg/d | 2,807 | 2.6 (1.6) | 4.8 (2.5) | 2.0 (1.9) | 0.6 (1.8) | 30 | 2.7 (2.5) | 135 |
| Thiamine, mg/d | 2,807 | 0.4 (0.2) | 0.8 (03) | 0.3 (0.2) | 0.1 (0.2) | 33 | 0.5 (0.3) | 167 |
| Riboflavin, mg/d | 2,807 | 0.3 (0.1) | 0.5 (0.2) | 0.3 (0.2) | 0.03 (0.2) | 10 | 0.3 (0.3) | 100 |
| Niacin, mg/d | 2,807 | 7.8 (3.0) | 14.2 (4.3) | 5.7 (3.7) | 2.1 (3.0) | 37 | 8.5 (4.8) | 149 |
| Vitamin B6, mg/d | 2,807 | 0.7 (0.3) | 1.2 (0.5) | 0.5 (0.4) | 0.2 (0.3) | 40 | 0.7 (0.5) | 140 |
| Folate, μg/d | 2,807 | 76.3 (39.5) | 139.3 (60.4) | 54.5 (46.2) | 21.8 (47.2) | 40 | 84.8 (66.4) | 156 |
| Vitamin C, mg/d | 2,807 | 32.1 (22.8) | 58.6 (37.0) | 17.8 (23.7) | 14.2 (27.5) | 80 | 40.8 (39.3) | 229 |
|  |  |  |  |  |  |  |  |  |
| **Minerals** |  |  |  |  |  |  |  |  |
| Calcium, mg/d | 2,807 | 166.2 (95.1) | 305.0 (154.4) | 151.4 (181.4) | 14.8 (175.8) | 10 | 153.6 (199.4) | 101 |
| Iron, mg/d | 2,807 | 5.8 (2.7) | 10.6 (4.0) | 4.2 (3.2) | 1.6 (3.0) | 38 | 6.4 (4.4) | 152 |
| Sodium, mg/d | 2,807 | 2886.6 (1485.1) | 5278.5 (2346.3) | 1960.0 (1766.3) | 926.6 (2002.2) | 47 | 3318.4 (2812.6) | 169 |
| Potassium, mg/d | 2,807 | 850.4 (378.0) | 1553.6 (545.3) | 620.5 (400.2) | 229.9 (405.3) | 37 | 933.0 (588.2) | 150 |
| Magnesium, mg/d | 2,807 | 183.5 (68.7) | 335.7 (95.2) | 131.5 (81.2) | 52.0 (72.1) | 40 | 204.2 (110.2) | 155 |
| Zinc, mg/d | 2,807 | 4.8 (1.7) | 8.7 (2.4) | 3.6 (2.2) | 1.1 (1.9) | 31 | 5.1 (2.8) | 142 |

^1^ Dietary factors presented had adequate data/information for the present analysis.

^2^ Sample sizes differ because we performed paired analysis for each dietary factor, i.e., for each analysis we used only the individuals with available intake data for both diet assessments.

^3^ Bangladesh Integrated Household Survey (BIHS) 2011-2012 provided household-level dietary data from a 7-day household consumption questionnaire and individual-level data from 24-hour recalls (24hR). Household consumption was individualized by applying a) the Adult Male Equivalent (AME) method,^12^ as proposed by FAO^14^, assuming moderate physical activity, and b) the per capita (PC) approach assuming equal distribution among household members (Appendix B). Individual intake was estimated from 24hR.

^4^ Absolute differences correspond to AME-24hR and PC-24hR respectively, and percentage differences correspond to absolute difference/24hR*100.

^5^ Differences between means were significant for all dietary factors (paired t-test, P<0.01) with the exception of meat/eggs (P=0.63), and SFA (P=0.24).

^6^ Differences between means were significant for all dietary factors (paired t-test, P<0.001).

MUFA, Monounsaturated fats; PUFA, Polyunsaturated fats; SFA, Saturated fat

# Table G. Comparison of individualized household consumption and individual dietary intake estimates by dietary factor in children 6-10 years old in the 2011-2012 BIHS.

| **Dietary Factor, unit^1^** | **Observations (n)^2^** | **Consumption**  **(mean, SD)^3^** | | | **Difference between 24hR and AME (mean, SD)^4^** | | **Difference between 24hR and PC (mean, SD)^4^** | |
| --- | --- | --- | --- | --- | --- | --- | --- | --- |
|  |  | **AME^3^** | **PC^3^** | **24hR^3^** | **Absolute^5^** | **Percent** | **Absolute^6^** | **Percent** |
| **Food groups** |  |  |  |  |  |  |  |  |
| Fruits, g/d | 3,076 | 21.8 (37.4) | 26.8 (45.3) | 9.4 (36.4) | 12.3 (45.4) | 131 | 17.4 (50.6) | 185 |
| Non-starchy vegetables, g/d | 3,078 | 190.8 (108.6) | 236.1 (132.5) | 124.9 (112.4) | 65.9 (136.1) | 53 | 111.2 (152.2) | 89 |
| Starchy vegetables, g/d | 3,031 | 79.2 (46.1) | 97.9 (55.9) | 83.9 (75.6) | -4.7 (76.0) | -6 | 14.0 (80.0) | 17 |
| Legumes, g/d | 2,776 | 16.6 (24.4) | 20.4 (29.4) | 14.5 (37.9) | 2.1 (37.0) | 14 | 5.9 (39.1) | 41 |
| Total grains, g/d | 1,775 | 1139.8 (319.8) | 1405.7 (364.6) | 1050.7 (329.3) | 89.0 (337.2) | 8 | 355.0 (394.4) | 34 |
| Meat/Eggs, g/d | 1,972 | 12.7 (14.9) | 15.7 (18.1) | 9.7 (20.5) | 3.0 (20.4) | 31 | 6.1 (21.6) | 63 |
| Seafood, g/d | 3,078 | 18.2 (16.4) | 22.5 (20.1) | 18.5 (22.5) | -0.3 (21.3) | -2 | 4.0 (22.8) | 22 |
| Milk, whole fat, g/d | 2,944 | 17.9 (36.0) | 22.1 (44.3) | 16.1 (65.6) | 1.7 (59.9) | 11 | 6.0 (61.1) | 37 |
| Fats/Oils, g/d | 3,075 | 14.7 (9.0) | 18.1 (10.7) | 13.4 (11.6) | 1.3 (11.7) | 10 | 4.8 (12.6) | 36 |
|  |  |  |  |  |  |  |  |  |
| **Energy & Macronutrients** |  |  |  |  |  |  |  |  |
| Energy, kcal/d | 3,078 | 1762.3 (504.5) | 2178.9 (580.6) | 1596.7 (475.1) | 165.6 (515.4) | 10 | 582.2 (600.4) | 36 |
| Protein, g/d | 3,078 | 42.4 (14.1) | 52.5 (16.6) | 38.5 (14.1) | 3.9 (14.8) | 10 | 14.0 (17.0) | 36 |
| Carbohydrates, g/d | 3,078 | 339.8 (95.6) | 420.1 (110.2) | 308.0 (91.8) | 31.8 (98.7) | 10 | 112.1 (115.6) | 36 |
| Total fat, g/d | 3,078 | 21.7 (12.5) | 26.8 (14.9) | 19.7 (14.7) | 2.0 (14.5) | 10 | 7.1 (15.6) | 36 |
| SFA, g/d | 3,078 | 4.7 (3.7) | 5.8 (4.3) | 4.2 (4.1) | 0.5 (4.5) | 12 | 1.6 (4.9) | 38 |
| MUFA, g/d | 3,078 | 6.6 (3.5) | 8.1 (4.2) | 6.0 (4.5) | 0.5 (4.2) | 8 | 2.1 (4.5) | 35 |
| PUFA, g/d | 3,078 | 10.0 (6.2) | 12.4 (7.4) | 9.2 (7.5) | 0.8 (7.2) | 9 | 3.2 (7.8) | 35 |
| Cholesterol, mg/d | 3,078 | 31.0 (33.2) | 38.4 (40.0) | 26.1 (46.4) | 4.9 (45.1) | 19 | 12.3 (47.4) | 47 |
| Fiber, g/d | 3,078 | 23.6 (7.4) | 29.2 (8.8) | 19.9 (6.8) | 3.8 (7.9) | 19 | 9.4 (9.2) | 47 |
|  |  |  |  |  |  |  |  |  |
| **Vitamins** |  |  |  |  |  |  |  |  |
| Vitamin A, μg RAE/d | 3,078 | 241.4 (247.8) | 297.6 (303.9) | 163.0 (283.9) | 78.4 (310.5) | 48 | 134.6 (342.7) | 83 |
| Vitamin D, μg/d | 3,078 | 0.9 (1.4) | 1.2 (1.7) | 0.8 (2.0) | 0.1 (1.9) | 13 | 0.3 (2.0) | 38 |
| Vitamin E, mg/d | 3,078 | 4.0 (2.2) | 4.9 (2.6) | 3.4 (2.5) | 0.5 (2.4) | 15 | 1.5 (2.6) | 44 |
| Thiamine, mg/d | 3,078 | 0.7 (0.2) | 0.9 (0.3) | 0.6 (0.2) | 0.1 (0.3) | 17 | 0.3 (0.3) | 50 |
| Riboflavin, mg/d | 3,078 | 0.4 (0.2) | 0.5 (0.2) | 0.4 (0.2) | 0.1 (0.2) | 25 | 0.2 (0.2) | 50 |
| Niacin, mg/d | 3,078 | 12.2 (4.0) | 15.1 (4.7) | 10.9 (3.8) | 1.3 (3.7) | 12 | 4.2 (4.3) | 39 |
| Vitamin B6, mg/d | 3,078 | 1.0 (0.4) | 1.3 (0.5) | 0.9 (0.4) | 0.1 (0.4) | 11 | 0.4 (0.4) | 44 |
| Folate, μg/d | 3,078 | 116.1 (52.0) | 143.6 (62.7) | 94.4 (59.5) | 21.6 (66.2) | 23 | 49.2 (72.7) | 52 |
| Vitamin C, mg/d | 3,078 | 47.8 (29.6) | 59.2 (36.2) | 33.2 (32.2) | 14.6 (39.1) | 44 | 26.0 (43.3) | 78 |
|  |  |  |  |  |  |  |  |  |
| **Minerals** |  |  |  |  |  |  |  |  |
| Calcium, mg/d | 3,078 | 251.5 (128.1) | 311.3 (156.5) | 213.9 (173.9) | 37.6 (171.3) | 18 | 97.4 (184.5) | 46 |
| Iron, mg/d | 3,078 | 9.0 (3.5) | 11.1 (4.2) | 7.6 (3.3) | 1.4 (3.6) | 18 | 3.5 (4.1) | 46 |
| Sodium, mg/d | 3,078 | 4377.1 (2013.7) | 5406.3 (2403.5) | 3270.2 (2016.8) | 1106.9 (2660.8) | 34 | 2136.0 (2959.9) | 65 |
| Potassium, mg/d | 3,078 | 1300.2 (477.4) | 1608.1 (567.1) | 1077.1 (418.0) | 223.1 (500.8) | 21 | 530.9 (576.5) | 49 |
| Magnesium, mg/d | 3,078 | 285.3 (85.6) | 352.8 (99.8) | 246.1 (76.9) | 39.3 (88.5) | 16 | 106.8 (103.9) | 43 |
| Zinc, mg/d | 3,078 | 7.4 (2.2) | 9.2 (2.6) | 6.6 (2.1) | 0.8 (2.3) | 12 | 2.5 (2.6) | 38 |

^1^ Dietary factors presented had adequate data/information for the present analysis.

^2^ Sample sizes differ because we performed paired analysis for each dietary factor, i.e., for each analysis we used only the individuals with available intake data for both diet assessments.

^3^ Bangladesh Integrated Household Survey (BIHS) 2011-2012 provided household-level dietary data from a 7-day household consumption questionnaire and individual-level data from 24-hour recalls (24hR). Household consumption was individualized by applying a) the Adult Male Equivalent (AME) method,^12^ as proposed by FAO^14^, assuming moderate physical activity, and b) the per capita (PC) approach assuming equal distribution among household members (Appendix B). Individual intake was estimated from 24hR.

^4^ Absolute differences correspond to HH PC-24hR and HH AME-24hR respectively, and percentage differences correspond to absolute difference/24hR*100.

^5^ Differences between means were significant for all dietary factors (paired t-test, P<0.01), with the exception of seafood (p=0.42), and milk (p=0.11).

^6^ Differences between means were significant for all dietary factors (paired t-test, P<0.0001).

MUFA, Monounsaturated fats; PUFA, Polyunsaturated fats; SFA, Saturated fat

# Table H. Comparison of individualized household consumption and individual dietary intake estimates by dietary factor in adolescents 11-19 years old in the 2011-2012 BIHS.

| **Dietary Factor, unit^1^** | **Observations (n)^2^** | **Consumption**  **(mean, SD)^3^** | | | **Difference between 24hR and AME (mean, SD)^4^** | | **Difference between 24hR and PC (mean, SD)^4^** | |
| --- | --- | --- | --- | --- | --- | --- | --- | --- |
|  |  | **AME^3^** | **PC^3^** | **24hR^3^** | **Absolute^5^** | **Percent** | **Absolute^6^** | **Percent** |
| **Food groups** |  |  |  |  |  |  |  |  |
| Fruits, g/d | 3,877 | 32.5 (55.8) | 30.2 (51.0) | 9.2 (34.1) | 23.3 (58.1) | 253 | 21.0 (53.9) | 228 |
| Non-starchy vegetables, g/d | 3,885 | 286.5 (168.7) | 266.9 (156.3) | 178.0 (144.2) | 108.5 (182.2) | 61 | 89.0 (175.9) | 50 |
| Starchy vegetables, g/d | 3,847 | 116.5 (70.6) | 108.7 (65.9) | 110.6 (94.0) | 5.9 (99.3) | 5 | -2.0 (98.4) | -2 |
| Legumes, g/d | 3,448 | 24.5 (34.2) | 22.7 (31.3) | 21.8 (51.8) | 2.7 (50.5) | 12 | 0.9 (49.7) | 4 |
| Total grains, g/d | 2,350 | 1642.2 (454.7) | 1524.0 (394.0) | 1458.9 (422.1) | 183.3 (423.7) | 13 | 65.1 (429.9) | 4 |
| Meat/Eggs, g/d | 2,684 | 17.7 (20.9) | 16.5 (19.6) | 12.5 (26.4) | 5.2 (26.8) | 42 | 4.0 (26.4) | 32 |
| Seafood, g/d | 3,885 | 27.8 (26.0) | 25.9 (24.7) | 25.5 (28.6) | 2.3 (28.9) | 9 | 0.5 (28.5) | 2 |
| Milk, whole fat, g/d | 3,720 | 25.6 (52.0) | 24.0 (48.0) | 14.8 (49.0) | 10.9 (47.5) | 74 | 9.2 (45.4) | 62 |
| Fats/Oils, g/d | 3,884 | 21.7 (12.7) | 20.2 (11.8) | 18.4 (16.1) | 3.2 (15.6) | 17 | 1.7 (15.4) | 9 |
|  |  |  |  |  |  |  |  |  |
| **Energy & Macronutrients** |  |  |  |  |  |  |  |  |
| Energy, kcal/d | 3,885 | 2583.6 (736.7) | 2402.8 (651.2) | 2186.1 (625.6) | 397.5 (693.1) | 18 | 216.6 (695.5) | 10 |
| Protein, g/d | 3,885 | 62.7 (21.1) | 58.3 (19.1) | 52.8 (17.8) | 9.9 (20.0) | 19 | 5.6 (19.9) | 11 |
| Carbohydrates, g/d | 3,885 | 496.7 (138.3) | 461.8 (120.9) | 422.5 (119.6) | 74.3 (131.9) | 18 | 39.3 (132.7) | 90 |
| Total fat, g/d | 3,885 | 32.2 (17.7) | 30.0 (16.4) | 26.6 (19.6) | 5.6 (19.1) | 21 | 3.4 (18.7) | 13 |
| SFA, g/d | 3,885 | 7.1 (4.9) | 6.6 (4.6) | 5.6 (5.0) | 1.5 (5.2) | 17 | 1.0 (5.1) | 18 |
| MUFA, g/d | 3,885 | 9.7 (5.4) | 9.1 (5.1) | 8.1 (6.0) | 1.7 (5.6) | 21 | 1.0 (5.6) | 12 |
| PUFA, g/d | 3,885 | 14.8 (8.7) | 13.8 (8.0) | 12.7 (10.2) | 2.2 (9.6) | 17 | 1.1 (9.4) | 9 |
| Cholesterol, mg/d | 3,885 | 45.0 (45.9) | 42.0 (43.4) | 34.1 (55.8) | 11.0 (55.9) | 32 | 8.0 (55.3) | 23 |
| Fiber, g/d | 3,885 | 34.8 (11.1) | 32.4 (10.0) | 27.6 (9.1) | 7.2 (10.8) | 26 | 4.8 (10.7) | 17 |
|  |  |  |  |  |  |  |  |  |
| **Vitamins** |  |  |  |  |  |  |  |  |
| Vitamin A, μg RAE/d | 3,885 | 354.9 (371.7) | 330.3 (347.6) | 222.4 (423.2) | 132.4 (465.5) | 60 | 107.8 (455.8) | 48 |
| Vitamin D, μg/d | 3,885 | 1.4 (1.9) | 1.3 (1.8) | 1.2 (2.6) | 0.2 (2.4) | 17 | 0.1 (2.4) | 8 |
| Vitamin E, mg/d | 3,885 | 5.9 (3.1) | 5.5 (2.8) | 4.7 (3.3) | 1.2 (3.2) | 26 | 0.7 (3.1) | 15 |
| Thiamine, mg/d | 3,885 | 1.0 (0.4) | 0.9 (0.3) | 0.8 (0.3) | 0.2 (0.4) | 25 | 0.1 (0.3) | 13 |
| Riboflavin, mg/d | 3,885 | 0.6 (0.3) | 0.6 (0.3) | 0.5 (0.2) | 0.1 (0.3) | 20 | 0.1 (0.3) | 20 |
| Niacin, mg/d | 3,885 | 17.6 (5.8) | 16.4 (5.2) | 14.8 (4.9) | 2.8 (4.9) | 19 | 1.6 (4.9) | 11 |
| Vitamin B6, mg/d | 3,885 | 1.5 (0.7) | 1.4 (0.6) | 1.3 (0.6) | 0.3 (0.5) | 23 | 0.2 (0.5) | 15 |
| Folate, μg/d | 3,885 | 172.2 (77.9) | 160.4 (71.8) | 128.2 (73.1) | 44.0 (85.6) | 34 | 32.2 (83.4) | 25 |
| Vitamin C, mg/d | 3,885 | 71.5 (45.5) | 66.7 (42.2) | 44.4 (40.8) | 27.1 (51.4) | 61 | 22.2 (49.6) | 50 |
|  |  |  |  |  |  |  |  |  |
| **Minerals** |  |  |  |  |  |  |  |  |
| Calcium, mg/d | 3,885 | 375.1 (207.6) | 349.7 (196.7) | 282.6 (202.7) | 92.5 (212.7) | 33 | 67.1 (209.9) | 24 |
| Iron, mg/d | 3,885 | 13.3 (5.3) | 12.4 (4.9) | 10.4 (4.4) | 2.9 (5.0) | 28 | 1.9 (5.0) | 18 |
| Sodium, mg/d | 3,885 | 6271.7 (2951.1) | 5831.1 (2689.0) | 4293.1 (2569.6) | 1978.7 (3515.1) | 46 | 1538.1 (3367.3) | 36 |
| Potassium, mg/d | 3,885 | 1915.2 (719.2) | 1783.5 (659.3) | 1462.8 (530.2) | 452.3 (685.6) | 31 | 320.6 (668.9) | 22 |
| Magnesium, mg/d | 3,885 | 419.1 (126.8) | 389.9 (113.4) | 339.3 (101.9) | 79.8 (120.1) | 24 | 50.6 (119.7) | 15 |
| Zinc, mg/d | 3,885 | 10.8 (3.2) | 10.1 (2.9) | 9.1 (2.7) | 1.8 (3.1) | 20 | 1.0 (3.1) | 11 |

^1^ Dietary factors presented had adequate data/information for the present analysis.

^2^ Sample sizes differ because we performed paired analysis for each dietary factor, i.e., for each analysis we used only the individuals with available intake data for both diet assessments.

^3^ Bangladesh Integrated Household Survey (BIHS) 2011-2012 provided household-level dietary data from a 7-day household consumption questionnaire and individual-level data from 24-hour recalls (24hR). Household consumption was individualized by applying a) the Adult Male Equivalent (AME) method,^12^ as proposed by FAO^14^, assuming moderate physical activity, and b) the per capita (PC) approach assuming equal distribution among household members (Appendix B). Individual intake was estimated from 24hR.

^4^ Absolute differences correspond to AME-24hR and PC-24hR respectively, and percentage differences correspond to absolute difference/24hR*100.

^5^ Differences between means were significant for all dietary factors (paired t-test, P<0.01).

^6^ Differences between means were significant for all dietary factors (paired t-test, P<0.01), with the exception of starchy vegetables (P=0.22), legumes (P=0.27), and seafood (P=0.30).

MUFA, Monounsaturated fats; PUFA, Polyunsaturated fats; SFA, Saturated fat

# Table I. Comparison of individualized household consumption and individual dietary intake estimates by dietary factor in adults 20-44 years old in the 2011-2012 BIHS.

| **Dietary Factor, unit^1^** | **Observations (n)^2^** | **Consumption**  **(mean, SD)^3^** | | | **Difference between 24hR and AME (mean, SD)^4^** | | **Difference between 24hR and PC (mean, SD)^4^** | |
| --- | --- | --- | --- | --- | --- | --- | --- | --- |
|  |  | **AME^3^** | **PC^3^** | **24hR^3^** | **Absolute^5^** | **Percent** | **Absolute^6^** | **Percent** |
| **Food groups** |  |  |  |  |  |  |  |  |
| Fruits, g/d | 7,719 | 36.3 (58.8) | 30.7 (49.7) | 9.1 (39.2) | 27.2 (61.6) | 299 | 21.6 (55.0) | 237 |
| Non-starchy vegetables, g/d | 7,728 | 301.6 (169.3) | 256.8 (146.5) | 201.5 (155.6) | 100.1 (191.0) | 50 | 55.3 (179.8) | 27 |
| Starchy vegetables, g/d | 7,634 | 124.1 (71.1) | 105.5 (61.4) | 127.8 (108.6) | -3.8 (107.5) | -3 | -22.4 (106.4) | -18 |
| Legumes, g/d | 6,873 | 25.7 (34.7) | 22.0 (29.8) | 22.9 (54.9) | 2.9 (54.1) | 13 | -0.9 (53.0) | -4 |
| Total grains, g/d | 4,534 | 1698.7 (457.2) | 1450.7 (395.2) | 1660.0 (465.3) | 38.6 (468.2) | 2 | -209.4 (488.8) | -13 |
| Meat/Eggs, g/d | 5,236 | 20.6 (22.7) | 17.6 (19.4) | 13.1 (29.1) | 7.5 (30.2) | 57 | 4.5 (29.1) | 34 |
| Seafood, g/d | 7,728 | 30.4 (27.6) | 25.8 (23.4) | 29.7 (33.6) | 0.7 (32.8) | 2 | -3.9 (31.7) | -13 |
| Milk, whole fat, g/d | 7,355 | 30.5 (60.7) | 25.9 (52.2) | 14.3 (50.8) | 16.3 (53.0) | 114 | 11.7 (48.5) | 82 |
| Fats/Oils, g/d | 7,726 | 23.8 (13.6) | 20.3 (11.8) | 21.4 (17.3) | 2.3 (17.0) | 11 | -1.2 (16.5) | -6 |
|  |  |  |  |  |  |  |  |  |
| **Energy & Macronutrients** |  |  |  |  |  |  |  |  |
| Energy, kcal/d | 7,728 | 2690.7 (728.8) | 2287.4 (633.2) | 2476.9 (668.6) | 213.8 (732.7) | 9 | -189.5 (747.5) | -8 |
| Protein, g/d | 7,728 | 65.5 (21.1) | 55.7 (18.2) | 59.7 (20.1) | 5.8 (21.8) | 10 | -4.0 (21.8) | -7 |
| Carbohydrates, g/d | 7,728 | 513.6 (137.6) | 436.5 (118.7) | 478.6 (128.9) | 35.0 (139.9) | 7 | -42.1 (143.4) | -9 |
| Total fat, g/d | 7,728 | 35.1 (18.9) | 29.9 (16.3) | 30.1 (20.8) | 5.0 (20.5) | 17 | -0.2 (19.8) | -1 |
| SFA, g/d | 7,728 | 7.6 (5.8) | 6.5 (4.9) | 6.1 (4.9) | 1.5 (5.9) | 25 | 0.4 (5.4) | 7 |
| MUFA, g/d | 7,728 | 10.6 (5.5) | 9.0 (4.8) | 9.0 (6.1) | 1.6 (5.9) | 18 | 0.02 (5.7) | 0 |
| PUFA, g/d | 7,728 | 16.1 (9.3) | 13.8 (8.0) | 14.7 (11.1) | 1.4 (10.5) | 10 | -0.9 (10.2) | -6 |
| Cholesterol, mg/d | 7,728 | 51.9 (0.6) | 44.1 (44.5) | 37.7 (65.3) | 14.2 (67.4) | 38 | 6.5 (65.0) | 17 |
| Fiber, g/d | 7,728 | 36.3 (11.0) | 30.9 (9.6) | 31.3 (9.8) | 5.0 (11.6) | 16 | -0.4 (11.5) | -1 |
|  |  |  |  |  |  |  |  |  |
| **Vitamins** |  |  |  |  |  |  |  |  |
| Vitamin A, μg RAE/d | 7,728 | 367.7 (381.9) | 312.4 (322.5) | 248.3 (450.4) | 119.5 (488.9) | 48 | 64.1 (465.3) | 26 |
| Vitamin D, μg/d | 7,728 | 1.6 (2.1) | 1.3 (1.8) | 1.3 (3.1) | 0.2 (2.9) | 15 | 0.005 (2.9) | 0 |
| Vitamin E, mg/d | 7,728 | 6.3 (3.2) | 5.4 (2.8) | 5.4 (3.6) | 0.9 (3.4) | 17 | 0.03 (3.3) | 1 |
| Thiamine, mg/d | 7,728 | 1.1 (0.4) | 0.9 (0.3) | 0.9 (0.3) | 0.2 (0.4) | 22 | 0.00005 (0.4) | 0 |
| Riboflavin, mg/d | 7,728 | 0.7 (0.3) | 0.6 (0.2) | 0.5 (0.2) | 0.1 (0.3) | 20 | 0.03 (0.3) | 6 |
| Niacin, mg/d | 7,728 | 18.5 (5.6) | 15.8 (4.9) | 17.0 (5.4) | 1.5 (5.2) | 9 | -1.2 (5.4) | -7 |
| Vitamin B6, mg/d | 7,728 | 1.6 (0.7) | 1.3 (0.6) | 1.4 (0.7) | 0.2 (0.6) | 14 | -0.1 (0.6) | -7 |
| Folate, μg/d | 7,728 | 183.0 (79.3) | 155.8 (69.1) | 142.7 (79.1) | 40.3 (92.0) | 28 | 13.1 (88.5) | 9 |
| Vitamin C, mg/d | 7,728 | 76.4 (47.0) | 65.1 (40.8) | 50.4 (43.3) | 25.9 (54.9) | 51 | 14.6 (51.5) | 29 |
|  |  |  |  |  |  |  |  |  |
| **Minerals** |  |  |  |  |  |  |  |  |
| Calcium, mg/d | 7,728 | 399.8 (212.2) | 339.8 (182.6) | 318.4 (239.2) | 81.4 (248.4) | 26 | 21.4 (240.9) | 7 |
| Iron, mg/d | 7,728 | 13.8 (5.3) | 11.7 (4.6) | 11.8 (4.8) | 2.1 (5.4) | 18 | -0.007 (5.3) | 0 |
| Sodium, mg/d | 7,728 | 6798.3 (3088.4) | 5772.8 (2646.9) | 5036.0 (3560.7) | 1762.4 (4426.1) | 35 | 736.8 (4210.8) | 15 |
| Potassium, mg/d | 7,728 | 2025.3 (714.2) | 1722.6 (622.6) | 1659.6 (573.9) | 365.6 (716.7) | 22 | 62.9 (693.2) | 4 |
| Magnesium, mg/d | 7,728 | 436.6 (125.7) | 371.0 (108.7) | 385.8 (110.8) | 50.8 (129.0) | 13 | -14.8 (129.3) | -4 |
| Zinc, mg/d | 7,728 | 11.4 (3.2) | 9.7 (2.8) | 10.3 (2.9) | 1.0 (3.3) | 10 | -0.7 (3.3) | -7 |

^1^ Dietary factors presented had adequate data/information for the present analysis.

^2^ Sample sizes differ because we performed paired analysis for each dietary factor, i.e., for each analysis we used only the individuals with available intake data for both diet assessments.

^3^ Bangladesh Integrated Household Survey (BIHS) 2011-2012 provided household-level dietary data from a 7-day household consumption questionnaire and individual-level data from 24-hour recalls (24hR). Household consumption was individualized by applying a) the Adult Male Equivalent (AME) method,^12^ as proposed by FAO^14^, assuming moderate physical activity, and b) the per capita (PC) approach assuming equal distribution among household members (Appendix B). Individual intake was estimated from 24hR.

^4^ Absolute differences correspond to AME-24hR and PC-24hR respectively, and percentage differences correspond to absolute difference/24hR*100.

^5^ Differences between means were significant for all dietary factors (paired t-test, P<0.01), with the exception of seafood (P=0.05).

^6^ Differences between means were significant for all dietary factors (paired t-test, P<0.001), with the exception of legumes (P=0.17), total fat (P=0.32), MUFA (P=0.70), thiamine (P=0.99), vitamin D (P=0.88), vitamin E (P=0.47), and iron (P=0.91).

MUFA, Monounsaturated fats; PUFA, Polyunsaturated fats; SFA, Saturated fat

# Table J. Comparison of individualized household consumption and individual dietary intake estimates by dietary factor in adults over 45 years of age in the 2011-2012 BIHS.

| **Dietary Factor, unit^1^** | **Observations (n)^2^** | **Consumption**  **(mean, SD)^3^** | | | **Difference between 24hR and AME (mean, SD)^4^** | | **Difference between 24hR and PC (mean, SD)^4^** | |
| --- | --- | --- | --- | --- | --- | --- | --- | --- |
|  |  | **AME^3^** | **PC^3^** | **24hR^3^** | **Absolute^5^** | **Percent** | **Absolute^6^** | **Percent** |
| **Food groups** |  |  |  |  |  |  |  |  |
| Fruits, g/d | 4,688 | 35.1 (58.4) | 33.7 (55.0) | 8.4 (33.0) | 26.7 (59.6) | 318 | 25.3 (56.6) | 301 |
| Non-starchy vegetables, g/d | 4,675 | 296.5 (174.3) | 285.4 (168.6) | 193.1 (152.0 ) | 103.4 (192.7) | 54 | 92.3 (191.0) | 48 |
| Starchy vegetables, g/d | 4,637 | 115.2 (73.0) | 110.7 (69.5) | 112.5 (98.2) | 2.7 (101.8) | 2 | -1.7 (101.2) | -2 |
| Legumes, g/d | 4,277 | 26.4 (36.1) | 25.5 (35.0) | 24.5 (56.6) | 1.9 (55.5) | 8 | 1.0 (55.5) | 4 |
| Total grains, g/d | 3,064 | 1605.4 (452.8) | 1553.5 (421.6) | 1496.5 (453.4) | 109.0 (473.4) | 7 | 57.0 (499.7) | 4 |
| Meat/Eggs, g/d | 3,363 | 18.4 (21.9) | 17.8 (21.2) | 12.0 (27.5) | 6.5 (28.1) | 54 | 5.8 (28.1) | 48 |
| Seafood, g/d | 4,675 | 28.8 (25.0) | 27.8 (24.3) | 28.2 (31.7) | 0.6 (30.9) | 2 | -0.4 (30.8) | -1 |
| Milk, whole fat, g/d | 4,477 | 34.0 (63.8) | 32.6 (61.6) | 20.3 (61.8) | 13.7 (56.7) | 67 | 12.3 (55.9) | 61 |
| Fats/Oils, g/d/ | 4,670 | 23.2 (13.9) | 22.4 (13.5) | 20.3 (16.7) | 2.9 (16.8) | 14 | 2.1 (16.8) | 10 |
|  |  |  |  |  |  |  |  |  |
| **Energy & Macronutrients** |  |  |  |  |  |  |  |  |
| Energy, kcal/d | 4,675 | 2581.7 (772.2) | 2480.0 (724.4) | 2301.6 (690.0) | 280.1 (770.6) | 12 | 178.4 (808.9) | 8 |
| Protein, g/d | 4,675 | 63.2 (22.2) | 60.7 (21.2) | 56.0 (20.3) | 7.2 (22.4) | 13 | 4.7 (23.1) | 8 |
| Carbohydrates, g/d | 4,675 | 490.9 (142.7) | 471.4 (132.5) | 442.6 (131.0) | 48.3 (145.4) | 11 | 28.7 (153.1) | 6 |
| Total fat, g/d | 4,675 | 34.4 (19.8) | 33.1 (19.0) | 28.7 (20.6) | 5.7 (21.0) | 20 | 4.4 (21.0) | 15 |
| SFA, g/d | 4,675 | 7.6 (6.2) | 7.3 (5.9) | 5.9 (5.0) | 1.7 (6.2) | 29 | 1.4 (6.1) | 24 |
| MUFA, g/d | 4,675 | 10.5 (6.1) | 10.1 (5.9) | 8.7 (6.5) | 1.8 (6.3) | 21 | 1.4 (6.3) | 16 |
| PUFA, g/d | 4,675 | 15.5 (9.3) | 15.0 (9.0) | 13.7 (10.7) | 1.8 (10.3) | 13 | 1.3 (10.3) | 9 |
| Cholesterol, mg/d | 4,675 | 49.2 (50.6) | 47.3 (48.3) | 35.6 (58.4) | 13.6 (60.7) | 38 | 11.7 (60.2) | 33 |
| Fiber, g/d | 4,675 | 35.1 (11.8) | 33.7 (11.1) | 29.3 (10.1) | 5.8 (12.1) | 20 | 4.4 (12.5) | 15 |
|  |  |  |  |  |  |  |  |  |
| **Vitamins** |  |  |  |  |  |  |  |  |
| Vitamin A, μg RAE/d | 4,675 | 374.1 (398.0) | 360.1 (384.2) | 251.7 (446.4) | 122.3 (486.7) | 49 | 108.4 (482.7 | 43 |
| Vitamin D, μg/d | 4,675 | 1.5 (2.1) | 1.5 (1.9) | 1.4 (3.0) | 0.2 (2.9) | 14 | 0.1 (2.8) | 7 |
| Vitamin E, mg/d | 4,675 | 6.1 (3.3) | 5.8 (3.2) | 5.1 (3.5) | 1.0 (3.4) | 20 | 0.8 (3.4) | 16 |
| Thiamine, mg/d | 4,675 | 1.0 (0.4) | 1.0 (0.4) | 0.9 (0.3) | 0.2 (0.4) | 22 | 0.1 (0.4) | 11 |
| Riboflavin, mg/d | 4,675 | 0.7 (0.3) | 0.6 (0.3) | 0.5 (0.2) | 0.1 (0.3) | 20 | 0.1 (0.3) | 20 |
| Niacin, mg/d | 4,675 | 17.8 (5.8) | 17.1 (5.4) | 15.8 (5.3) | 2.0 (5.4) | 13 | 1.3 (5.6) | 8 |
| Vitamin B6, mg/d | 4,675 | 1.5 (0.7) | 1.5 (0.6) | 1.3 (0.6) | 0.2 (0.6) | 15 | 0.2 (0.6) | 15 |
| Folate, μg/d | 4,675 | 177.9 (83.8) | 171.2 (80.2) | 134.0 (75.9) | 44.0 (93.7) | 33 | 37.2 (93.5) | 28 |
| Vitamin C, mg/d | 4,675 | 73.9 (48.0) | 71.1 (45.7) | 47.1 (42.6) | 26.9 (54.4) | 57 | 24.0 (53.1) | 51 |
|  |  |  |  |  |  |  |  |  |
| **Minerals** |  |  |  |  |  |  |  |  |
| Calcium, mg/d | 4,675 | 390.8 (213.5) | 376.0 (205.4) | 307.6 (225.8) | 83.2 (231.3) | 27 | 68.4 (231.5) | 22 |
| Iron, mg/d | 4,675 | 13.5 (5.7) | 13.0 (5.4) | 11.3 (5.7) | 2.2 (6.2) | 19 | 1.7 (6.3) | 15 |
| Sodium, mg/d | 4,675 | 6705.5 (3297.9) | 6452.8 (3194.6) | 4816.2 (2948.8) | 1889.3 (3981.3) | 39 | 1636.5 (3964.7) | 34 |
| Potassium, mg/d | 4,675 | 1973.0 (769.7) | 1896.2 (732.8) | 1573.4 (607.1) | 399.5 (771.6) | 25 | 322.8 (780.9 | 21 |
| Magnesium, mg/d | 4,675 | 419.7 (133.5) | 403.3 (126.1) | 359.9 (116.6) | 59.8 (136.4) | 17 | 43.4 (142.0) | 12 |
| Zinc, mg/d | 4,675 | 10.9 (3.4) | 10.5 (3.2) | 9.6 (3.0) | 1.3 (3.5) | 14 | 0.9 (3.6) | 9 |

^1^ Dietary factors presented had adequate data/information for the present analysis.

^2^ Sample sizes differ because we performed paired analysis for each dietary factor, i.e., for each analysis we used only the individuals with available intake data for both diet assessments.

^3^ Bangladesh Integrated Household Survey (BIHS) 2011-2012 provided household-level dietary data from a 7-day household consumption questionnaire and individual-level data from 24-hour recalls (24hR). Household consumption was individualized by applying a) the Adult Male Equivalent (AME) method,^12^ as proposed by FAO^14^, assuming moderate physical activity, and b) the per capita (PC) approach assuming equal distribution among household members (Appendix B). Individual intake was estimated from 24hR.

^4^ Absolute differences correspond to AME-24hR and PC-24hR respectively, and percentage differences correspond to absolute difference/24hR*100.

^5^ Differences between means were significant for all dietary factors (paired t-test, P<0.05), with the exception of starchy vegetables (P=0.07), and seafood (P=0.19).

^6^ Differences between means were significant for all dietary factors (paired t-test, P<0.05), with the exception of starchy vegetables (P=0.24), legumes (P=0.23), and seafood (P=0.35).

MUFA, Monounsaturated fats; PUFA, Polyunsaturated fats; SFA, Saturated fat

# Table K. Comparison of individualized household consumption and individual dietary intake estimates by dietary factor in adults (≥20 years old) of low educational level (<6 years) in the 2011-2012 BIHS.

| **Dietary Factor, unit^1^** | **Observations (n)^2^** | **Consumption**  **(mean, SD)^3^** | | | **Difference between 24hR and AME (mean, SD)^4^** | | **Difference between 24hR and PC (mean, SD)^4^** | |
| --- | --- | --- | --- | --- | --- | --- | --- | --- |
|  |  | **AME^3^** | **PC^3^** | **24hR^3^** | **Absolute^5^** | **Percent** | **Absolute^6^** | **Percent** |
| **Food groups** |  |  |  |  |  |  |  |  |
| Fruits, g/d | 8920 | 30.0 (52.6) | 27.2 (47.4) | 7.7 (34.7) | 22.3 (55.0) | 290 | 19.4 (51.2) | 252 |
| Non-starchy vegetables, g/d | 8934 | 287.9 (167) | 260.9 (155.1) | 191.5 (148.4) | 96.4 (187.8) | 50 | 69.4 (182.9) | 36 |
| Starchy vegetables, g/d | 8852 | 119.7 (71.3) | 108.0 (65.4) | 122.6 (105.6) | -3.0 (104.8) | -2 | -14.6 (105.4) | -12 |
| Legumes, g/d | 7978 | 23.7 (33.6) | 21.6 (31.1) | 21.6 (53.9) | 2.1 (52.3) | 10 | 0.1 (51.9) | 0 |
| Total grains, g/d | 5706 | 1655.5 (450.5) | 1505.7 (405.0) | 1601.2 (471.3) | 54.3 (464.7) | 3 | -95.5 (513.4) | -6 |
| Meat/Eggs, g/d | 6004 | 16.7 (20.2) | 15.3 (18.7) | 10.7 (26.5) | 6.0 (27.2) | 56 | 4.6 (26.8) | 43 |
| Seafood, g/d | 8934 | 27.7 (25.8) | 25.0 (23.4) | 27.7 (32.0) | 0.01 (30.9) | 0 | -2.6 (30.4) | -9 |
| Milk, whole fat, g/d | 8539 | 26.4 (55.3) | 24.2 (51.7) | 13.9 (50.9) | 12.4 (49.6) | 89 | 10.2 (47.8) | 73 |
| Fats/Oils, g/d/ | 8927 | 21.5 (12.4) | 19.6 (11.7) | 19.1 (15.7) | 2.4 (15.7) | 13 | 0.4 (15.6) | 2 |
|  |  |  |  |  |  |  |  |  |
| **Energy & Macronutrients** |  |  |  |  |  |  |  |  |
| Energy, kcal/d | 8934 | 2583.6 (720.3) | 2332.6 (663.9) | 2376.1 (681.4) | 207.5 (718.0) | 9 | -43.5 (785.3) | -2 |
| Protein, g/d | 8934 | 62.5 (20.5) | 56.5 (19) | 57.1 (20.2) | 5.4 (21.0) | 9 | -0.6 (22.3) | -1 |
| Carbohydrates, g/d | 8934 | 497.4 (137.9) | 449.1 (124.8) | 463.1 (132.2) | 34.9 (138.8) | 8 | -14 (151.9) | -3 |
| Total fat, g/d | 8934 | 31.7 (16.8) | 28.8 (15.9) | 27.2 (19.2) | 4.5 (19.0) | 17 | 1.7 (19.0) | 6 |
| SFA, g/d | 8934 | 6.8 (4.7) | 6.2 (4.4) | 5.6 (4.7) | 1.2 (5.1) | 21 | 0.6 (5.0) | 11 |
| MUFA, g/d | 8934 | 9.9 (5.3) | 9.0 (5.0) | 8.4 (6.0) | 1.5 (5.7) | 18 | 0.6 (5.8) | 7 |
| PUFA, g/d | 8934 | 14.5 (8.4) | 13.2 (7.9) | 13.0 (10.1) | 1.4 (9.6) | 11 | 0.1 (9.6) | 1 |
| Cholesterol, mg/d | 8933 | 43.6 (44.3) | 39.6 (40.7) | 31.5 (52.5) | 12 (55.8) | 38 | 8.1 (54.5) | 26 |
| Fiber, g/d | 8934 | 35.0 (10.9) | 31.6 (10.1) | 30.2 (9.8) | 4.9 (11.3) | 16 | 1.5 (12.0) | 5 |
|  |  |  |  |  |  |  |  |  |
| **Vitamins** |  |  |  |  |  |  |  |  |
| Vitamin A, μg RAE/d | 8934 | 351.1 (377.2) | 318.1 (343.2) | 240.0 (452.5) | 111.1 (485.6) | 46 | 78.1 (473.6) | 33 |
| Vitamin D, μg/d | 8934 | 1.4 (2.0) | 1.3 (1.8) | 1.2 (2.9) | 0.2 (2.8) | 17 | 0.05 (2.7) | 4 |
| Vitamin E, mg/d | 8934 | 5.7 (3.0) | 5.2 (2.8) | 4.9 (3.3) | 0.9 (3.2) | 18 | 0.3 (3.2) | 6 |
| Thiamine, mg/d | 8934 | 1.0 (0.4) | 0.9 (0.3) | 0.9 (0.3) | 0.2 (0.4) | 22 | 0.1 (0.4) | 11 |
| Riboflavin, mg/d | 8934 | 0.6 (0.3) | 0.6 (0.2) | 0.5 (0.2) | 0.1 (0.3) | 20 | 0.1 (0.3) | 20 |
| Niacin, mg/d | 8934 | 18.0 (5.6) | 16.2 (5.1) | 16.5 (5.4) | 1.5 (5.2) | 9 | -0.2 (5.6) | -1 |
| Vitamin B6, mg/d | 8934 | 1.5 (0.7) | 1.4 (0.6) | 1.3 (0.6) | 0.2 (0.6) | 15 | 0.02 (0.6) | 2 |
| Folate, μg/d | 8934 | 173.6 (77.8) | 157.2 (72.8) | 134.8 (73.2) | 38.8 (87.6) | 29 | 22.4 (87.5) | 17 |
| Vitamin C, mg/d | 8934 | 71.5 (44.4) | 64.8 (41.0) | 48.0 (41.2) | 23.6 (51.8) | 49 | 16.8 (50.2) | 35 |
|  |  |  |  |  |  |  |  |  |
| **Minerals** |  |  |  |  |  |  |  |  |
| Calcium, mg/d | 8934 | 375.1 (197.1) | 339.5 (182.4) | 301.7 (228.4) | 73.4 (233.3) | 24 | 37.8 (232.9) | 13 |
| Iron, mg/d | 8934 | 13.3 (5.2) | 12.0 (4.9) | 11.3 (5.2) | 2.0 (5.6) | 18 | 0.7 (5.7) | 6 |
| Sodium, mg/d | 8934 | 6638.5 (3091.4) | 6001.6 (2874.4) | 4905.2 (3342.6) | 1733.3 (4222.3) | 35 | 1096.3 (4152.5) | 22 |
| Potassium, mg/d | 8934 | 1932.3 (695.5) | 1747.9 (653.5) | 1587.0 (572.9) | 345.3 (706.7) | 22 | 160.9 (726.9) | 10 |
| Magnesium, mg/d | 8934 | 421.2 (125.0) | 380.2 (114.9) | 371.9 (113.6) | 49.3 (127.5) | 13 | 8.3 (136.1) | 2 |
| Zinc, mg/d | 8934 | 10.9 (3.2) | 9.9 (2.9) | 9.9 (3.0) | 1.0 (3.2) | 10 | -0.04 (3.5) | 0 |

^1^ Dietary factors presented had adequate data/information for the present analysis.

^2^ Sample sizes differ because we performed paired analysis for each dietary factor, i.e., for each analysis we used only the individuals with available intake data for both diet assessments.

^3^ Bangladesh Integrated Household Survey (BIHS) 2011-2012 provided household-level dietary data from a 7-day household consumption questionnaire and individual-level data from 24-hour recalls (24hR). Household consumption was individualized by applying a) the Adult Male Equivalent (AME) method,^12^ as proposed by FAO^14^, assuming moderate physical activity, and b) the per capita (PC) approach assuming equal distribution among household members (Appendix B). Individual intake was estimated from 24hR.

^4^ Absolute differences correspond to AME-24hR and PC-24hR respectively, and percentage differences correspond to absolute difference/24hR*100.

^5^ Differences between means were significant for all dietary factors (paired t-test, P<0.01), with the exception of seafood (P=0.98).

^6^ Differences between means were significant for all dietary factors (paired t-test, P<0.01), with the exception of legumes (P=0.89), PUFA (P=0.22), vitamin D (P=0.12), and zinc (P=0.23).

MUFA, Monounsaturated fats; PUFA, Polyunsaturated fats; SFA, Saturated fat

# Table L. Comparison of individualized household consumption and individual dietary intake estimates by dietary factor in adults (≥20 years old) of medium and high educational level (≥6 years) in the 2011-2012 BIHS.

| **Dietary Factor, unit^1^** | **Observations (n)^2^** | **Consumption**  **(mean, SD)^3^** | | | **Difference between 24hR and AME (mean, SD)^4^** | | **Difference between 24hR and PC (mean, SD)^4^** | |
| --- | --- | --- | --- | --- | --- | --- | --- | --- |
|  |  | **AME^3^** | **PC^3^** | **24hR^3^** | **Absolute^5^** | **Percent** | **Absolute^6^** | **Percent** |
| **Food groups** |  |  |  |  |  |  |  |  |
| Fruits, g/d | 3467 | 51.0 (69.7) | 43.9 (59.9) | 11.8 (42.3) | 39.2 (72.4) | 332 | 32.1 (64.8) | 272 |
| Non-starchy vegetables, g/d | 3469 | 330.0 (178.2) | 284.8 (156.4) | 216.0 (167.4) | 114.0 (200.6) | 53 | 68.8 (190.1) | 32 |
| Starchy vegetables, g/d | 3419 | 123.5 (73.7) | 106.0 (62.4) | 120.5 (103.6) | 3.0 (106.9) | 2 | -14.5 (103.7) | -12 |
| Legumes, g/d | 3172 | 31.9 (0.7) | 27.6 (33.6) | 28.4 (59.3) | 3.5 (60.2) | 12 | -0.7 (58.8) | -2 |
| Total grains, g/d | 1892 | 1677.8 (478.5) | 1451.2 (418.8) | 1572.6 (455.3) | 105.2 (489.3) | 7 | -121.4 (499.9) | -8 |
| Meat/Eggs, g/d | 2595 | 26.7 (25.6) | 23.0 (22.2) | 17.1 (32.2) | 9.6 (33.8) | 56 | 5.9 (32.7) | 35 |
| Seafood, g/d | 3469 | 35.2 (28.0) | 30.4 (24.2) | 32.8 (35.0) | 2.4 (34.8) | 7 | -2.4 (33.8) | -7 |
| Milk, whole fat, g/d | 3293 | 46.0 (74.6) | 39.5 (64.6) | 23.3 (65.0) | 22.7 (64.8) | 97 | 16.2 (59.6) | 70 |
| Fats/Oils, g/d/ | 3469 | 28.8 (15.4) | 24.9 (13.6) | 25.8 (19.3) | 3.0 (19.7) | 12 | -0.9 (19.2) | -3 |
|  |  |  |  |  |  |  |  |  |
| **Energy & Macronutrients** |  |  |  |  |  |  |  |  |
| Energy, kcal/d | 3469 | 2819.6 (787.8) | 2430.6 (699.8) | 2500.3 (675.7) | 319.4 (814.5) | 13 | -69.7 (807.3) | -3 |
| Protein, g/d | 3469 | 70.0 (23.1) | 60.4 (20.5) | 61.2 (20.2) | 8.8 (24.3) | 14 | -0.9 (23.7) | -1 |
| Carbohydrates, g/d | 3469 | 523.4 (143.5) | 451.0 (126.5) | 470.2 (127.3) | 53.1 (149.6) | 11 | -19.2 (149.0) | -4 |
| Total fat, g/d | 3469 | 42.9 (22.4) | 37.0 (19.7) | 35.8 (23.0) | 7.1 (24.5) | 20 | 1.2 (23.5) | 3 |
| SFA, g/d | 3469 | 9.6 (8.1) | 8.3 (6.9) | 7.2 (5.3) | 2.4 (8.0) | 33 | 1.1 (7.1) | 15 |
| MUFA, g/d | 3469 | 12.3 (6.4) | 10.6 (5.7) | 10.2 (6.7) | 2.1 (6.7) | 21 | 0.4 (6.5) | 4 |
| PUFA, g/d | 3469 | 19.6 (10.3) | 16.9 (9.0) | 17.6 (12.4) | 2.0 (12.3) | 11 | -0.7 (12.0) | -4 |
| Cholesterol, mg/d | 3469 | 69.7 (62.3) | 60.1 (54.7) | 50.7 (82.1) | 19 (83.7) | 37 | 9.4 (81.7) | 19 |
| Fiber, g/d | 3469 | 38.0 (12.1) | 32.7 (10.7) | 31.5 (10.3) | 6.5 (12.8) | 21 | 1.2 (12.5) | 4 |
|  |  |  |  |  |  |  |  |  |
| **Vitamins** |  |  |  |  |  |  |  |  |
| Vitamin A, μg RAE/d | 3469 | 419.1 (410.6) | 362.0 (357.3) | 274.2 (438.6) | 144.9 (493.5) | 53 | 87.8 (469.5) | 32 |
| Vitamin D, μg/d | 3469 | 1.9 (2.4) | 1.6 (2.0) | 1.6 (3.4) | 0.3 (3.2) | 19 | 0.03 (3.1) | 2 |
| Vitamin E, mg/d | 3469 | 7.5 (3.6) | 6.4 (3.1) | 6.3 (4.0) | 1.2 (4.0) | 19 | 0.1 (3.8) | 2 |
| Thiamine, mg/d | 3469 | 1.1 (0.4) | 1.0 (0.3) | 0.9 (0.3) | 0.2 (0.4) | 22 | 0.04 (0.4) | 4 |
| Riboflavin, mg/d | 3469 | 0.8 (0.3) | 0.7 (0.3) | 0.6 (0.2) | 0.2 (0.3) | 33 | 0.1 (0.3) | 17 |
| Niacin, mg/d | 3469 | 19.0 (5.9) | 16.3 (5.2) | 16.8 (5.2) | 2.2 (5.5) | 13 | -0.4 (5.6) | -2 |
| Vitamin B6, mg/d | 3469 | 1.7 (0.7) | 1.4 (0.6) | 1.4 (0.6) | 0.2 (0.6) | 14 | 0.01 (0.6) | 1 |
| Folate, μg/d | 3469 | 200.5 (85.9) | 172.9 (75.3) | 151.3 (88.2) | 49.2 (104.3) | 33 | 21.6 (100.1) | 14 |
| Vitamin C, mg/d | 3469 | 85.6 (53.2) | 73.8 (46.5) | 52.2 (47.3) | 33.3 (61.1) | 64 | 21.6 (57.3) | 41 |
|  |  |  |  |  |  |  |  |  |
| **Minerals** |  |  |  |  |  |  |  |  |
| Calcium, mg/d | 3469 | 451.2 (239.9) | 389.3 (211.6) | 346.8 (245.8) | 104.4 (262.0) | 30 | 42.5 (252.4) | 12 |
| Iron, mg/d | 3469 | 14.8 (5.8) | 12.8 (5.1) | 12.3 (5.2) | 2.5 (6.1) | 20 | 0.4 (5.9) | 3 |
| Sodium, mg/d | 3469 | 7084.8 (3340.3) | 6099.9 (2909.2) | 5076.5 (3347.9) | 2008.3 (4364.4) | 40 | 1023.4 (4117.2) | 20 |
| Potassium, mg/d | 3469 | 2194.2 (801.0) | 1891.4 (705.5) | 1730.6 (613.5) | 463.6 (807.2) | 27 | 160.8 (766.9) | 9 |
| Magnesium, mg/d | 3469 | 453.4 (135.9) | 390.9 (120.5) | 386.7 (113.3) | 66.8 (142.1) | 17 | 4.3 (139.6) | 1 |
| Zinc, mg/d | 3469 | 11.9 (3.5) | 10.2 (3.1) | 10.4 (3.0) | 1.5 (3.7) | 14 | -0.2 (3.6) | -2 |

^1^ Dietary factors presented had adequate data/information for the present analysis.

^2^ Sample sizes differ because we performed paired analysis for each dietary factor, i.e., for each analysis we used only the individuals with available intake data for both diet assessments.

^3^ Bangladesh Integrated Household Survey (BIHS) 2011-2012 provided household-level dietary data from a 7-day household consumption questionnaire and individual-level data from 24-hour recalls (24hR). Household consumption was individualized by applying a) the Adult Male Equivalent (AME) method,^12^ as proposed by FAO^14^, assuming moderate physical activity, and b) the per capita (PC) approach assuming equal distribution among household members (Appendix B). Individual intake was estimated from 24hR.

^4^ Absolute differences correspond to AME-24hR and PC-24hR respectively, and percentage differences correspond to absolute difference/24hR*100.

^5^ Differences between means were significant for all dietary factors (paired t-test, P<0.001), with the exception of starchy vegetables (P=0.10).

^6^ Differences between means were significant for all dietary factors (paired t-test, P<0.05), with the exception of legumes (P=0.48), vitamin B_6_ (P=0.15), vitamin D (P=0.51), and magnesium (P=0.07).

MUFA, Monounsaturated fats; PUFA, Polyunsaturated fats; SFA, Saturated fat

# Table M. Comparison of individualized household consumption and individual dietary intake estimates by dietary factor in Muslims^1^ in the 2011-2012 BIHS.

| **Dietary Factor, unit^2^** | **Observations (n)^3^** | **Consumption**  **(mean, SD)^4^** | | | **Difference between 24hR and AME (mean, SD)^5^** | | **Difference between 24hR and PC (mean, SD)^5^** | |
| --- | --- | --- | --- | --- | --- | --- | --- | --- |
|  |  | **HH AME^4^** | **HH PC^4^** | **24hR^4^** | **Absolute^6^** | **Percent** | **Absolute^7^** | **Percent** |
| **Food groups** |  |  |  |  |  |  |  |  |
| Fruits, g/d | 19712 | 30.8 (53.8) | 30.6 (51.0) | 9.0 (35.9) | 21.9 (56.6) | 243 | 21.6 (54.3) | 240 |
| Non-starchy vegetables, g/d | 19735 | 255.9 (164.6) | 254.0 (148.5) | 164.6 (146.5) | 91.3 (172.6) | 55 | 89.4 (175.0) | 54 |
| Starchy vegetables, g/d | 19493 | 104.7 (69.4) | 103.9 (62.6) | 102.1 (96.8) | 2.6 (94.9) | 3 | 1.8 (98.4) | 2 |
| Legumes, g/d | 17581 | 21.7 (32.3) | 21.6 (30.9) | 19.0 (48.8) | 2.7 (47.7) | 14 | 2.6 (47.8) | 14 |
| Total grains, g/d | 11805 | 1463.9 (535.5) | 1452.9 (400.4) | 1361.6 (563.5) | 102.3 (428.6) | 8 | 91.3 (559.1) | 7 |
| Meat/Eggs, g/d | 19735 | 25.5 (23.8) | 25.3 (22.2) | 24.8 (29.7) | 5.3 (27.5) | 21 | 5.2 (27.3) | 21 |
| Seafood, g/d | 13376 | 17.6 (21.0) | 17.5 (19.9) | 12.3 (26.9) | 0.7 (28.4) | 6 | 0.5 (29.0) | 4 |
| Milk, whole fat, g/d | 18930 | 25.6 (53.4) | 25.4 (51.3) | 17.3 (60.7) | 8.3 (59.1) | 48 | 8.1 (55.6) | 47 |
| Fats/Oils, g/d/ | 19729 | 20.2 (13.2) | 20.1 (11.9) | 17.6 (15.9) | 2.6 (15.2) | 15 | 2.5 (15.7) | 14 |
|  |  |  |  |  |  |  |  |  |
| **Energy & Macronutrients** |  |  |  |  |  |  |  |  |
| Energy, kcal/d | 19735 | 2303.9 (868.5) | 2286.5 (652.5) | 2046.8 (813.9) | 257.0 (678.9) | 13 | 239.6 (845.3) | 12 |
| Protein, g/d | 19735 | 56.1 (23.6) | 55.6 (18.8) | 49.7 (22.2) | 6.4 (19.8) | 13 | 6.0 (23.2) | 12 |
| Carbohydrates, g/d | 19735 | 440.2 (163.2) | 436.9 (121.1) | 394.2 (157.4) | 46.0 (129.0) | 12 | 42.7 (163.2) | 11 |
| Total fat, g/d | 19735 | 29.9 (18.7) | 29.7 (16.8) | 25.4 (19.6) | 4.5 (18.8) | 18 | 4.3 (19.3) | 17 |
| SFA, g/d | 19735 | 6.5 (5.5) | 6.5 (5.1) | 5.3 (4.8) | 1.2 (5.5) | 23 | 1.2 (5.5) | 23 |
| MUFA, g/d | 19735 | 9.0 (5.5) | 8.9 (4.9) | 7.6 (5.8) | 1.4 (5.5) | 18 | 1.3 (5.7) | 17 |
| PUFA, g/d | 19735 | 13.8 (9.0) | 13.7 (8.1) | 12.2 (10.2) | 1.6 (9.4) | 13 | 1.5 (9.8) | 12 |
| Cholesterol, mg/d | 19734 | 43.5 (47.2) | 43.2 (44.5) | 33.7 (58.0) | 9.8 (59.7) | 29 | 9.4 (58.9) | 28 |
| Fiber, g/d | 19735 | 31.0 (12.6) | 30.8 (9.9) | 25.7 (11.3) | 5.4 (10.6) | 21 | 5.1 (12.4) | 20 |
|  |  |  |  |  |  |  |  |  |
| **Vitamins** |  |  |  |  |  |  |  |  |
| Vitamin A, μg RAE/d | 19735 | 315.8 (352.3) | 313.5 (333.1) | 209.4 (398.8) | 106.4 (436.7) | 51 | 104.2 (435.6) | 50 |
| Vitamin D, μg/d | 19735 | 1.3 (1.9) | 1.3 (1.8) | 1.1 (2.7) | 0.2 (2.5) | 18 | 0.2 (2.5) | 18 |
| Vitamin E, mg/d | 19735 | 5.4 (3.2) | 5.3 (2.8) | 4.5 (3.4) | 0.9 (3.1) | 20 | 0.9 (3.2) | 20 |
| Thiamine, mg/d | 19735 | 0.9 (0.4) | 0.9 (0.3) | 0.8 (0.3) | 0.2 (0.3) | 25 | 0.2 (0.4) | 25 |
| Riboflavin, mg/d | 19735 | 0.6 (0.3) | 0.6 (0.2) | 0.5 (0.2) | 0.1 (0.3) | 20 | 0.1 (0.3) | 20 |
| Niacin, mg/d | 19735 | 15.9 (6.4) | 15.8 (5.0) | 14.1 (6.2) | 1.9 (4.8) | 13 | 1.7 (6.0) | 12 |
| Vitamin B6, mg/d | 19735 | 1.4 (0.7) | 1.3 (0.6) | 1.2 (0.6) | 0.2 (0.5) | 17 | 0.2 (0.6) | 17 |
| Folate, μg/d | 19735 | 155.4 (81.3) | 154.2 (70.2) | 118.8 (76.4) | 36.6 (83.5) | 31 | 35.4 (86.6) | 30 |
| Vitamin C, mg/d | 19735 | 64.1 (44.3) | 63.7 (40.2) | 41.0 (40.1) | 23.1 (49.1) | 56 | 22.7 (48.9) | 55 |
|  |  |  |  |  |  |  |  |  |
| **Minerals** |  |  |  |  |  |  |  |  |
| Calcium, mg/d | 19735 | 336.6 (200.0) | 334.0 (177.0) | 269.2 (216.1) | 67.4 (217.5) | 25 | 64.9 (220.6) | 24 |
| Iron, mg/d | 19735 | 11.8 (5.6) | 11.7 (4.6) | 9.7 (5.2) | 2.1 (5.1) | 22 | 2.0 (5.6) | 21 |
| Sodium, mg/d | 19735 | 5857.0 (3163.5) | 5813.8 (2755.0) | 4165.6 (3062.1) | 1691.3 (3752.4) | 41 | 1648.2 (3796.9) | 40 |
| Potassium, mg/d | 19735 | 1730.3 (778.2) | 1717.1 (640.6) | 1381.5 (634.9) | 348.8 (668.9) | 25 | 335.6 (733.3) | 24 |
| Magnesium, mg/d | 19735 | 373.1 (145.4) | 370.3 (111.5) | 317.2 (132.1) | 56.0 (118.3) | 18 | 53.1 (142.4) | 17 |
| Zinc, mg/d | 19735 | 9.7 (3.8) | 9.7 (2.9) | 8.5 (3.5) | 1.2 (3.0) | 14 | 1.1 (3.7) | 13 |

^1^ Religion data was available only for household head. We assumed that other household members were of the same religion.

^2^ Dietary factors presented had adequate data/information for the present analysis.

^3^ Sample sizes differ because we performed paired analysis for each dietary factor, i.e., for each analysis we used only the individuals with available intake data for both diet assessments.

^4^ Bangladesh Integrated Household Survey (BIHS) 2011-2012 provided household-level dietary data from a 7-day household consumption questionnaire and individual-level data from 24-hour recalls (24hR). Household consumption was individualized by applying a) the Adult Male Equivalent (AME) method,^12^ as proposed by FAO^14^, assuming moderate physical activity, and b) the per capita (PC) approach assuming equal distribution among household members (Appendix B). Individual intake was estimated from 24hR.

^5^ Absolute differences correspond to AME-24hR and PC-24hR respectively, and percentage differences correspond to absolute difference/24hR*100.

^6^ Differences between means were significant for all dietary factors (paired t-test, P<0.001).

^7^ Differences between means were significant for all dietary factors (paired t-test, P<0.05).

MUFA, Monounsaturated fats; PUFA, Polyunsaturated fats; SFA, Saturated fat

# Table N. Comparison of individualized household consumption and individual dietary intake estimates by dietary factor among other religions^1^ in the 2011-2012 BIHS.

| **Dietary Factor, unit^2^** | **Observations (n)^3^** | **Consumption**  **(mean, SD)^4^** | | | **Difference between 24hR and AME (mean, SD)^5^** | | **Difference between 24hR and PC (mean, SD)^5^** | |
| --- | --- | --- | --- | --- | --- | --- | --- | --- |
|  |  | **AME^4^** | **PC^4^** | **24hR^4^** | **Absolute^6^** | **Percent** | **Absolute^7^** | **Percent** |
| **Food groups** |  |  |  |  |  |  |  |  |
| Fruits, g/d | 2434 | 30.4 (34.7) | 30.1 (46.0) | 9.2 (34.7) | 21.2 (53) | 230 | 21.0 (51.5) | 228 |
| Non-starchy vegetables, g/d | 2438 | 295.3 (151.2) | 293.4 (162.1) | 189.3 (151.2) | 105.9 (185.2) | 56 | 104.0 (188.6) | 55 |
| Starchy vegetables, g/d | 2426 | 112.7 (103.2) | 112.1 (62.1) | 125.6 (103.2) | -12.9 (97.8) | -10 | -13.4 (102.7) | -11 |
| Legumes, g/d | 2304 | 28.0 (58.8) | 27.7 (30.3) | 28.5 (58.8) | -0.5 (57.5) | -2 | -0.7 (57.7) | -2 |
| Total grains, g/d | 1505 | 1551.2 (565.1) | 1545.6 (386.4) | 1411.3 (565.1) | 140.0 (442.6) | 10 | 134.3 (575.9) | 10 |
| Meat/Eggs, g/d | 2438 | 26.6 (31.7) | 26.4 (29.4) | 22.5 (31.7) | 5.9 (21.6) | 26 | 5.8 (21.3) | 26 |
| Seafood, g/d | 1643 | 13.9 (20.7) | 13.8 (15.0) | 8.0 (20.7) | 4.2 (29.7) | 53 | 3.9 (30.1) | 49 |
| Milk, whole fat, g/d | 2271 | 32.5 (60.6) | 32.3 (56.9) | 21.2 (60.6) | 11.3 (50.0) | 53 | 11.2 (46.9) | 53 |
| Fats/Oils, g/d/ | 2433 | 20.5 (17.4) | 20.4 (12.1) | 19.7 (17.4) | 0.9 (15.2) | 5 | 0.7 (15.7) | 4 |
|  |  |  |  |  |  |  |  |  |
| **Energy & Macronutrients** |  |  |  |  |  |  |  |  |
| Energy, kcal/d | 2438 | 2472.8 (843.2) | 2458.6 (650.6) | 2208.8 (843.2) | 264.0 (684.1) | 12 | 249.8 (861.0) | 11 |
| Protein, g/d | 2438 | 60.1 (22.3) | 59.8 (18.9) | 52.2 (22.3) | 7.9 (19.7) | 15 | 7.5 (23.1) | 14 |
| Carbohydrates, g/d | 2438 | 475.6 (164.3) | 472.9 (124.4) | 426.2 (164.3) | 49.4 (132.5) | 12 | 46.7 (168.7) | 11 |
| Total fat, g/d | 2438 | 30.5 (20.4) | 30.3 (16.1) | 27.4 (20.4) | 3.1 (18) | 11 | 2.9 (18.6) | 11 |
| SFA, g/d | 2438 | 6.6 (4.8) | 6.5 (4.8) | 5.4 (4.8) | 1.1 (5.3) | 20 | 1.1 (2.3) | 20 |
| MUFA, g/d | 2438 | 9.9 (7.1) | 9.9 (5.5) | 8.9 (7.1) | 1.0 (5.7) | 11 | 0.9 (5.9) | 10 |
| PUFA, g/d | 2438 | 13.2 (10.8) | 13.2 (7.9) | 12.5 (10.8) | 0.7 (9.0) | 6 | 0.6 (9.4) | 5 |
| Cholesterol, mg/d | 2438 | 42.3 (64.5) | 41.9 (39.7) | 31.1 (64.5) | 11.2 (59.4) | 36 | 10.9 (59.3) | 35 |
| Fiber, g/d | 2438 | 33.9 (12.1) | 33.7 (10.2) | 28.5 (12.1) | 5.4 (11.1) | 19 | 5.2 (13.1) | 18 |
|  |  |  |  |  |  |  |  |  |
| **Vitamins** |  |  |  |  |  |  |  |  |
| Vitamin A, μg RAE/d | 2438 | 379.2 (448.2) | 376.7 (361.9) | 259.2 (448.2) | 120.0 (460.1) | 46 | 117.4 (462.6) | 45 |
| Vitamin D, μg/d | 2438 | 1.4 (2.6) | 1.3 (1.9) | 1.1 (2.6) | 0.3 (2.6) | 27 | 0.3 (2.6) | 27 |
| Vitamin E, mg/d | 2438 | 5.4 (3.5) | 5.3 (2.9) | 4.6 (3.5) | 0.8 (3.0) | 17 | 0.7 (3.2) | 15 |
| Thiamine, mg/d | 2438 | 1.0 (0.4) | 1.0 (0.3) | 0.8 (0.4) | 0.2 (0.4) | 25 | 0.2 (0.4) | 25 |
| Riboflavin, mg/d | 2438 | 0.6 (0.3) | 0.6 (0.3) | 0.5 (0.3) | 0.1 (0.2) | 20 | 0.1 (0.3) | 20 |
| Niacin, mg/d | 2438 | 16.2 (6.2) | 16.1 (5.3) | 14.0 (6.2) | 2.2 (4.8) | 16 | 2.1 (5.9) | 15 |
| Vitamin B6, mg/d | 2438 | 1.5 (0.7) | 1.5 (0.6) | 1.3 (0.7) | 0.2 (0.5) | 15 | 0.2 (0.6) | 15 |
| Folate, μg/d | 2438 | 172.1 (82.3) | 170.9 (75.1) | 133.9 (82.3) | 38.2 (87.9) | 29 | 37.0 (91.5) | 28 |
| Vitamin C, mg/d | 2438 | 76.1 (43.9) | 75.7 (47.7) | 51.6 (43.9) | 24.5 (54.9) | 47 | 24.1 (55.4) | 47 |
|  |  |  |  |  |  |  |  |  |
| **Minerals** |  |  |  |  |  |  |  |  |
| Calcium, mg/d | 2438 | 398.5 (266.9) | 395.8 (233.7) | 315.0 (266.9) | 83.5 (257.1) | 27 | 80.8 (260.5) | 26 |
| Iron, mg/d | 2438 | 13.4 (5.8) | 13.3 (5.4) | 11.2 (5.8) | 2.2 (5.2) | 20 | 2.1 (5.8) | 19 |
| Sodium, mg/d | 2438 | 5840.7 (3125.3) | 5805.6 (2610.1) | 4705.1 (3125.3) | 1135.6 (3572.7) | 24 | 1100.5 (3706.1) | 23 |
| Potassium, mg/d | 2438 | 1869.2 (660.6) | 1857.6 (687.3) | 1500.6 (660.6) | 368.6 (679.5) | 25 | 357.0 (751.9) | 24 |
| Magnesium, mg/d | 2438 | 407.7 (140.4) | 405.3 (118.5) | 348.4 (140.4) | 59.3 (124.4) | 17 | 56.9 (150.2) | 16 |
| Zinc, mg/d | 2438 | 10.3 (3.6) | 10.3 (2.9) | 9.1 (3.6) | 1.2 (3.1) | 13 | 1.2 (3.8) | 13 |

^1^ Religion data was available only for household head. We assumed that other household members were of the same religion. Other religions refer to Christians and Hindus.

^2^ Dietary factors presented had adequate data/information for the present analysis.

^3^ Sample sizes differ because we performed paired analysis for each dietary factor, i.e., for each analysis we used only the individuals with available intake data for both diet assessments.

^4^ Bangladesh Integrated Household Survey (BIHS) 2011-2012 provided household-level dietary data from a 7-day household consumption questionnaire and individual-level data from 24-hour recalls (24hR). Household consumption was individualized by applying a) the Adult Male Equivalent (AME) method,^12^ as proposed by FAO^14^, assuming moderate physical activity, and b) the per capita (PC) approach assuming equal distribution among household members (Appendix B). Individual intake was estimated from 24hR.

^5^ Absolute differences correspond to AME-24hR and PC-24hR respectively, and percentage differences correspond to absolute difference/24hR*100.

^6^ Differences between means were significant for all dietary factors (paired t-test, P<0.01), with the exception of legumes (P=0.67).

^7^ Differences between means were significant for all dietary factors (paired t-test, P<0.05), with the exception of legumes (P=0.54).

MUFA, Monounsaturated fats; PUFA, Polyunsaturated fats; SFA, Saturated fat

# Table O. Comparison of individualized household consumption and individual dietary intake estimates by dietary factor among individuals in the first quintile of household income in the 2011-2012 BIHS.

| **Dietary Factor, unit^1^** | **Observations (n)^2^** | **Consumption**  **(mean, SD)^3^** | | | **Difference between 24hR and AME mean, SD)^4^** | | **Difference between 24hR and PC (mean, SD)^4^** | |
| --- | --- | --- | --- | --- | --- | --- | --- | --- |
|  |  | **AME^3^** | **PC^3^** | **24hR^3^** | **Absolute^5^** | **Percent** | **Absolute^6^** | **Percent** |
| **Food groups** |  |  |  |  |  |  |  |  |
| Fruits, g/d | 4441 | 35.4 (59.3) | 35.2 (56.9) | 8.5 (37.6) | 26.9 (61.3) | 316 | 26.7 (59.5) | 314 |
| Non-starchy vegetables, g/d | 4441 | 268.1 (175.4) | 266.2 (160.7) | 160.3 (146.3) | 107.8 (181.6) | 67 | 105.8 (182.7) | 66 |
| Starchy vegetables, g/d | 4391 | 107.8 (73.9) | 106.9 (67.6) | 95.6 (92.2) | 12.2 (95.9) | 13 | 11.4 (97.8) | 12 |
| Legumes, g/d | 4144 | 25.2 (35.3) | 25.0 (33.6) | 22.5 (53.4) | 2.7 (52.0) | 12 | 2.5 (52.0) | 11 |
| Total grains, g/d | 2844 | 1434.9 (532.8) | 1425.9 (417.3) | 1284.5 (549.9) | 150.3 (437.9) | 12 | 141.4 (545.4) | 11 |
| Meat/Eggs, g/d | 4441 | 25.2 (23.1) | 25.0 (21.6) | 22.4 (27.6) | 7.2 (27.1) | 32 | 7.1 (26.7) | 32 |
| Seafood, g/d | 2983 | 18.8 (24.3) | 18.7 (23.2) | 11.6 (26.4) | 2.8 (27.5) | 24 | 2.6 (27.8) | 22 |
| Milk, whole fat, g/d | 4304 | 25.7 (54.9) | 25.5 (52.9) | 17.1 (57.9) | 8.6 (57.1) | 50 | 8.5 (53.4) | 50 |
| Fats/Oils, g/d/ | 4437 | 22.6 (15.2) | 22.4 (13.9) | 18.7 (17.4) | 3.9 (17.0) | 21 | 3.7 (17.4) | 20 |
|  |  |  |  |  |  |  |  |  |
| **Energy & Macronutrients** |  |  |  |  |  |  |  |  |
| Energy, kcal/d | 4441 | 2331.3 (906.7) | 2314.3 (721.7) | 1979.3 (791) | 352.0 (727.7) | 18 | 335.0 (856.9) | 17 |
| Protein, g/d | 4441 | 57.1 (24.7) | 56.6 (20.6) | 48.1 (21.7) | 9.0 (20.6) | 19 | 8.6 (23.2) | 18 |
| Carbohydrates, g/d | 4441 | 439.3 (166.7) | 436.1 (130.9) | 377.4 (151.8) | 61.9 (135.6) | 16 | 58.7 (162.6) | 16 |
| Total fat, g/d | 4441 | 32.9 (21.6) | 32.6 (19.7) | 26.3 (20.8) | 6.6 (20.8) | 25 | 6.4 (21.2) | 24 |
| SFA, g/d | 4441 | 7.1 (6.2) | 7.1 (5.8) | 5.4 (4.8) | 1.7 (5.8) | 31 | 1.7 (5.7) | 31 |
| MUFA, g/d | 4441 | 9.5 (6.2) | 9.4 (5.6) | 7.7 (6.2) | 1.8 (6.1) | 23 | 1.7 (6.2) | 22 |
| PUFA, g/d | 4441 | 15.3 (10.4) | 15.2 (9.5) | 12.7 (11.1) | 2.6 (10.5) | 20 | 2.5 (10.9) | 20 |
| Cholesterol, mg/d | 4440 | 45.4 (51.8) | 45.1 (49.1) | 31.8 (61.3) | 13.6 (64.3) | 43 | 13.3 (62.5) | 42 |
| Fiber, g/d | 4441 | 31.7 (13.2) | 31.5 (10.9) | 24.9 (11.3) | 6.8 (11.4) | 27 | 6.6 (12.8) | 27 |
|  |  |  |  |  |  |  |  |  |
| **Vitamins** |  |  |  |  |  |  |  |  |
| Vitamin A, μg RAE/d | 4441 | 343.1 (379.9) | 340.9 (361.7) | 211.1 (388.4) | 132.1 (459.7) | 63 | 129.9 (456.6) | 62 |
| Vitamin D, μg/d | 4441 | 1.2 (1.8) | 1.2 (1.7) | 1.1 (2.7) | 0.2 (2.4) | 18 | 0.2 (2.5) | 18 |
| Vitamin E, mg/d | 4441 | 6.0 (3.7) | 6.0 (3.3) | 4.7 (3.7) | 1.3 (3.6) | 28 | 1.2 (3.7) | 26 |
| Thiamine, mg/d | 4441 | 0.9 (0.4) | 0.9 (0.3) | 0.7 (0.3) | 0.2 (0.4) | 29 | 0.2 (0.4) | 29 |
| Riboflavin, mg/d | 4441 | 0.6 (0.3) | 0.6 (0.3) | 0.5 (0.2) | 0.1 (0.3) | 20 | 0.1 (0.3) | 20 |
| Niacin, mg/d | 4441 | 15.7 (6.4) | 15.6 (5.2) | 13.2 (5.9) | 2.5 (4.9) | 19 | 2.3 (5.9) | 17 |
| Vitamin B6, mg/d | 4441 | 1.4 (0.7) | 1.4 (0.7) | 1.1 (0.7) | 0.3 (0.6) | 27 | 0.3 (0.6) | 27 |
| Folate, μg/d | 4441 | 162.6 (87.2) | 161.4 (77.2) | 117.4 (78.2) | 45.2 (89.5) | 39 | 44.0 (91.6) | 37 |
| Vitamin C, mg/d | 4441 | 67.5 (48.0) | 66.9 (44.2) | 39.7 (42.4) | 27.7 (52.9) | 70 | 27.2 (52.5) | 69 |
|  |  |  |  |  |  |  |  |  |
| **Minerals** |  |  |  |  |  |  |  |  |
| Calcium, mg/d | 4441 | 348.3 (213.2) | 345.8 (192.1) | 262.6 (219.1) | 85.7 (238) | 33 | 83.2 (235.8) | 32 |
| Iron, mg/d | 4441 | 12.4 (6.1) | 12.3 (5.3) | 9.8 (5.2) | 2.6 (5.4) | 27 | 2.5 (5.9) | 26 |
| Sodium, mg/d | 4441 | 6208.3 (3491.8) | 6157.9 (3113.6) | 4158.8 (3122.6) | 2049.5 (4021.5) | 49 | 1999.1 (4018.9) | 48 |
| Potassium, mg/d | 4441 | 1805.2 (852.8) | 1792.0 (732.3) | 1353.3 (631.1) | 451.8 (733.5) | 33 | 438.6 (778.7) | 32 |
| Magnesium, mg/d | 4441 | 378.0 (152.4) | 375.2 (123.3) | 307.1 (130.0) | 70.9 (127.3) | 23 | 68.1 (146.6) | 22 |
| Zinc, mg/d | 4441 | 9.8 (3.9) | 9.7 (3.1) | 8.2 (3.4) | 1.6 (3.2) | 20 | 1.5 (3.7) | 18 |

^1^ Dietary factors presented had adequate data/information for the present analysis.

^2^ Sample sizes differ because we performed paired analysis for each dietary factor, i.e., for each analysis we used only the individuals with available intake data for both diet assessments.

^3^ Bangladesh Integrated Household Survey (BIHS) 2011-2012 provided household-level dietary data from a 7-day household consumption questionnaire and individual-level data from 24-hour recalls (24hR). Household consumption was individualized by applying a) the Adult Male Equivalent (AME) method,^12^ as proposed by FAO^14^, assuming moderate physical activity, and b) the per capita (PC) approach assuming equal distribution among household members (Appendix B). Individual intake was estimated from 24hR.

^4^ Absolute differences correspond to AME-24hR and PC-24hR respectively, and percentage differences correspond to absolute difference/24hR*100.

^5^ Differences between means were significant for all dietary factors (paired t-test, P<0.001).

^6^ Differences between means were significant for all dietary factors (paired t-test, P<0.01).

MUFA, Monounsaturated fats; PUFA, Polyunsaturated fats; SFA, Saturated fat

# Table P. Comparison of individualized household consumption and individual dietary intake estimates by dietary factor among individuals in the second quintile of household income in the 2011-2012 BIHS.

| **Dietary Factor, unit^1^** | **Observations (n)^2^** | **Consumption**  **(mean, SD)^3^** | | | **Difference between 24hR and AME (mean, SD)^4^** | | **Difference between 24hR and PC (mean, SD)^4^** | |
| --- | --- | --- | --- | --- | --- | --- | --- | --- |
|  |  | **AME^3^** | **PC^3^** | **24hR^3^** | **Absolute^5^** | **Percent** | **Absolute^6^** | **Percent** |
| **Food groups** |  |  |  |  |  |  |  |  |
| Fruits, g/d | 4439 | 25.1 (44.5) | 24.8 (42.1) | 8.6 (35.3) | 16.5 (51.2) | 192 | 16.2 (49.5) | 188 |
| Non-starchy vegetables, g/d | 4450 | 246.1 (161.4) | 243.9 (145.5) | 163.0 (140.9) | 83.1 (161.2) | 51 | 80.9 (165.3) | 50 |
| Starchy vegetables, g/d | 4419 | 107.4 (71.2) | 106.5 (64.2) | 103.2 (95.6) | 4.2 (93.2) | 4 | 3.3 (97.3) | 3 |
| Legumes, g/d | 3929 | 19.7 (30.6) | 19.5 (29.2) | 16.5 (47.1) | 3.2 (47.2) | 19 | 3.0 (47.0) | 18 |
| Total grains, g/d | 2782 | 1477.6 (538.0) | 1466.5 (403.0) | 1379.7 (558.8) | 97.9 (426.2) | 7 | 86.8 (560.8) | 6 |
| Meat/Eggs, g/d | 2845 | 23.2 (22.0) | 22.9 (20.4) | 24.0 (28.6) | 4.6 (23.7) | 19 | 4.5 (23.5) | 19 |
| Seafood, g/d | 4450 | 14.0 (18.4) | 13.8 (17.5) | 9.3 (23.7) | -0.8 (27.0) | -9 | -1.1 (27.7) | -12 |
| Milk, whole fat, g/d | 4217 | 20.3 (45.5) | 20.2 (44.0) | 12.9 (50.7) | 7.5 (48.5) | 58 | 7.3 (45.9) | 57 |
| Fats/Oils, g/d/ | 4448 | 18.2 (11.9) | 18.0 (10.7) | 16.0 (14.5) | 2.1 (13.5) | 13 | 1.9 (14.0) | 12 |
|  |  |  |  |  |  |  |  |  |
| **Energy & Macronutrients** |  |  |  |  |  |  |  |  |
| Energy, kcal/d | 4450 | 2264.2 (848.7) | 2244.9 (631.4) | 2036.0 (819.7) | 228.2 (637.8) | 11 | 208.9 (826.2) | 10 |
| Protein, g/d | 4450 | 54.3 (22.5) | 53.8 (17.8) | 48.6 (22.2) | 5.7 (18.8) | 12 | 5.3 (22.5) | 11 |
| Carbohydrates, g/d | 4450 | 439.3 (162.6) | 435.6 (119.7) | 397.4 (160.4) | 41.9 (124.6) | 11 | 38.2 (162.8) | 10 |
| Total fat, g/d | 4450 | 26.7 (16.3) | 26.4 (14.6) | 23.2 (18) | 3.5 (16.3) | 15 | 3.2 (17) | 14 |
| SFA, g/d | 4450 | 5.7 (4.2) | 5.7 (3.9) | 4.8 (4.4) | 0.9 (4.6) | 19 | 0.8 (4.7) | 17 |
| MUFA, g/d | 4450 | 8.3 (5.3) | 8.2 (4.8) | 7.2 (5.8) | 1.1 (4.9) | 15 | 1.0 (5.1) | 14 |
| PUFA, g/d | 4450 | 12.4 (8.0) | 12.2 (7.2) | 11.1 (9.3) | 1.2 (8.3) | 11 | 1.1 (8.7) | 10 |
| Cholesterol, mg/d | 4450 | 35.6 (40.6) | 35.2 (38.3) | 27.9 (49) | 7.7 (49.7) | 28 | 7.3 (48.7) | 26 |
| Fiber, g/d | 4450 | 30.6 (12.0) | 30.3 (9.2) | 25.6 (11.2) | 5.0 (9.9) | 20 | 4.7 (12.0) | 18 |
|  |  |  |  |  |  |  |  |  |
| **Vitamins** |  |  |  |  |  |  |  |  |
| Vitamin A, μg RAE/d | 4450 | 301.1 (352.2) | 298.4 (334.4) | 208.4 (423.7) | 92.7 (424.8) | 44 | 90.0 (425.4) | 43 |
| Vitamin D, μg/d | 4450 | 1.1 (1.8) | 1.1 (1.7) | 1.0 (2.5) | 0.1 (2.3) | 10 | 0.1 (2.3) | 10 |
| Vitamin E, mg/d | 4450 | 4.9 (2.8) | 4.8 (2.5) | 4.2 (3.1) | 0.7 (2.7) | 17 | 0.7 (2.8) | 17 |
| Thiamine, mg/d | 4450 | 0.9 (0.4) | 0.9 (0.3) | 0.7 (0.3) | 0.1 (0.3) | 14 | 0.1 (0.4) | 14 |
| Riboflavin, mg/d | 4450 | 0.5 (0.3) | 0.5 (0.2) | 0.4 (0.2) | 0.1 (0.2) | 25 | 0.1 (0.2) | 25 |
| Niacin, mg/d | 4450 | 15.6 (6.3) | 15.5 (5.0) | 13.9 (6.3) | 1.7 (4.7) | 12 | 1.5 (6.0) | 11 |
| Vitamin B6, mg/d | 4450 | 1.4 (0.7) | 1.3 (0.6) | 1.2 (0.7) | 0.2 (0.5) | 17 | 0.2 (0.6) | 17 |
| Folate, μg/d | 4450 | 148.2 (74.8) | 146.9 (63.7) | 115.1 (72.5) | 33.1 (75.9) | 29 | 31.8 (80.0) | 28 |
| Vitamin C, mg/d | 4450 | 61.5 (41.7) | 61.0 (38.0) | 39.9 (36.1) | 21.6 (44.4) | 54 | 21.1 (44.8) | 53 |
|  |  |  |  |  |  |  |  |  |
| **Minerals** |  |  |  |  |  |  |  |  |
| Calcium, mg/d | 4450 | 316.2 (186.5) | 313.3 (164.9) | 260.2 (224.9) | 56.0 (212.4) | 22 | 53.2 (218.7) | 20 |
| Iron, mg/d | 4450 | 11.5 (5.4) | 11.4 (4.5) | 9.6 (5.6) | 2.0 (5.3) | 21 | 1.9 (5.8) | 20 |
| Sodium, mg/d | 4450 | 5614.5 (3041.1) | 5569.7 (2667.1) | 4303.8 (3479.3) | 1310.7 (4020.8) | 30 | 1265.8 (4112.7) | 29 |
| Potassium, mg/d | 4450 | 1668.2 (729.1) | 1653.5 (591.1) | 1349.8 (640.9) | 318.3 (636.3) | 24 | 303.7 (712.6) | 22 |
| Magnesium, mg/d | 4450 | 368.8 (141.8) | 365.6 (107.4) | 317.1 (135.3) | 51.7 (114.2) | 16 | 48.6 (141.4) | 15 |
| Zinc, mg/d | 4450 | 9.5 (3.6) | 9.4 (2.8) | 8.4 (3.5) | 1.1 (2.8) | 13 | 1.0 (3.6) | 12 |

^1^ Dietary factors presented had adequate data/information for the present analysis.

^2^ Sample sizes differ because we performed paired analysis for each dietary factor, i.e., for each analysis we used only the individuals with available intake data for both diet assessments.

^3^ Bangladesh Integrated Household Survey (BIHS) 2011-2012 provided household-level dietary data from a 7-day household consumption questionnaire and individual-level data from 24-hour recalls (24hR). Household consumption was individualized by applying a) the Adult Male Equivalent (AME) method,^12^ as proposed by FAO^14^, assuming moderate physical activity, and b) the per capita (PC) approach assuming equal distribution among household members (Appendix B). Individual intake was estimated from 24hR.

^4^ Absolute differences correspond to AME-24hR and PC-24hR respectively, and percentage differences correspond to absolute difference/24hR*100.

^5^ Differences between means were significant for all dietary factors (paired t-test, P<0.05).

^6^ Differences between means were significant for all dietary factors (paired t-test, P<0.05).

MUFA, Monounsaturated fats; PUFA, Polyunsaturated fats; SFA, Saturated fat

# Table Q. Comparison of individualized household consumption and individual dietary intake estimates by dietary factor among individuals in the third quintile of household income in the 2011-2012 BIHS.

| **Dietary Factor, unit^1^** | **Observations (n)^2^** | **Consumption**  **(mean, SD)^3^** | | | **Difference between 24hR and AME (mean, SD)^4^** | | **Difference between 24hR and PC (mean, SD)^4^** | |
| --- | --- | --- | --- | --- | --- | --- | --- | --- |
|  |  | **AME^3^** | **PC^3^** | **24hR^3^** | **Absolute^5^** | **Percent** | **Absolute^6^** | **Percent** |
| **Food groups** |  |  |  |  |  |  |  |  |
| Fruits, g/d | 4425 | 26.9 (52.3) | 26.6 (49.5) | 7.8 (33.0) | 19.0 (54.3) | 244 | 18.8 (51.9) | 241 |
| Non-starchy vegetables, g/d | 4429 | 248.0 (160.4) | 245.8 (143.2) | 161.3 (140.5) | 86.7 (164) | 54 | 84.5 (166.6) | 52 |
| Starchy vegetables, g/d | 4394 | 103.8 (69.9) | 102.8 (62.7) | 105.9 (98.0) | -2.1 (95.0) | -2 | -3.1 (99.6) | -3 |
| Legumes, g/d | 3940 | 20.0 (30.6) | 19.8 (29.2) | 18.7 (48.4) | 1.3 (43.7) | 7 | 1.1 (44.3) | 6 |
| Total grains, g/d | 2818 | 1462.7 (529.3) | 1449.6 (368.7) | 1369.4 (568.2) | 93.3 (414.5) | 7 | 80.2 (568.9) | 6 |
| Meat/Eggs, g/d | 4429 | 23.2 (25.4) | 22.9 (23.7) | 22.3 (28.9) | 3.5 (26.9) | 16 | 3.3 (26.7) | 15 |
| Seafood, g/d | 3038 | 14.9 (18.3) | 14.8 (17.1) | 11.4 (27.1) | 0.9 (27.9) | 8 | 0.6 (28.4) | 5 |
| Milk, whole fat, g/d | 4266 | 20.9 (43.7) | 20.8 (41.9) | 14.5 (49.9) | 6.4 (47.4) | 44 | 6.3 (45.3) | 43 |
| Fats/Oils, g/d/ | 4424 | 17.8 (11.2) | 17.6 (9.9) | 16.3 (15.0) | 1.5 (13.8) | 9 | 1.3 (14.3) | 8 |
|  |  |  |  |  |  |  |  |  |
| **Energy & Macronutrients** |  |  |  |  |  |  |  |  |
| Energy, kcal/d | 4429 | 2244.8 (832.8) | 2224.5 (589.1) | 2023.7 (814.3) | 221.1 (622.5) | 11 | 200.8 (824.1) | 10 |
| Protein, g/d | 4429 | 54.1 (22.3) | 53.6 (17.0) | 48.8 (22.5) | 5.3 (18.6) | 11 | 4.8 (22.5) | 10 |
| Carbohydrates, g/d | 4429 | 435.2 (160.7) | 431.3 (112.8) | 393.2 (159.1) | 42.0 (121.2) | 11 | 38.1 (162.3) | 10 |
| Total fat, g/d | 4429 | 26.5 (15.4) | 26.3 (13.5) | 23.6 (18.2) | 2.9 (16.8) | 12 | 2.7 (17.4) | 11 |
| SFA, g/d | 4429 | 5.7 (4.0) | 5.7 (3.7) | 4.9 (4.0) | 0.9 (4.2) | 18 | 0.8 (4.2) | 16 |
| MUFA, g/d | 4429 | 8.4 (4.9) | 8.3 (4.3) | 7.4 (5.5) | 0.9 (5.1) | 12 | 0.9 (5.3) | 12 |
| PUFA, g/d | 4429 | 12.0 (7.7) | 11.9 (6.9) | 11.1 (9.8) | 0.9 (8.4) | 8 | 0.8 (8.8) | 7 |
| Cholesterol, mg/d | 4429 | 38.2 (41.6) | 37.8 (38.5) | 31.8 (61.1) | 6.3 (60.4) | 20 | 6.0 (60.6) | 19 |
| Fiber, g/d | 4429 | 30.3 (12.1) | 30.0 (9.2) | 25.5 (11.3) | 4.8 (9.8) | 19 | 4.5 (12.0) | 18 |
|  |  |  |  |  |  |  |  |  |
| **Vitamins** |  |  |  |  |  |  |  |  |
| Vitamin A, μg RAE/d | 4429 | 295.4 (340.1) | 292.6 (319.2) | 211.5 (399.0) | 83.9 (434.5) | 40 | 81.2 (434.1) | 38 |
| Vitamin D, μg/d | 4429 | 1.3 (1.8) | 1.3 (1.7) | 1.2 (2.9) | 0.1 (2.6) | 8 | 0.1 (2.7) | 8 |
| Vitamin E, mg/d | 4429 | 4.8 (2.8) | 4.7 (2.5) | 4.1 (3.2) | 0.7 (2.8) | 17 | 0.6 (2.9) | 15 |
| Thiamine, mg/d | 4429 | 0.9 (0.4) | 0.9 (0.3) | 0.7 (0.4) | 0.1 (0.3) | 14 | 0.1 (0.4) | 14 |
| Riboflavin, mg/d | 4429 | 0.5 (0.3) | 0.5 (0.2) | 0.5 (0.2) | 0.1 (0.2) | 20 | 0.1 (0.2) | 20 |
| Niacin, mg/d | 4429 | 15.8 (6.3) | 15.6 (4.8) | 14.1 (6.3) | 1.7 (4.6) | 12 | 1.5 (6.1) | 11 |
| Vitamin B6, mg/d | 4429 | 1.3 (0.6) | 1.3 (0.5) | 1.1 (0.6) | 0.2 (0.5) | 18 | 0.1 (0.6) | 9 |
| Folate, μg/d | 4429 | 149.2 (77.8) | 147.8 (65.8) | 114.7 (71.1) | 34.4 (76.0) | 30 | 33.1 (80.1) | 29 |
| Vitamin C, mg/d | 4429 | 61.3 (43.1) | 60.8 (38.8) | 40.8 (38.4) | 20.5 (45.9) | 50 | 20.0 (45.9) | 49 |
|  |  |  |  |  |  |  |  |  |
| **Minerals** |  |  |  |  |  |  |  |  |
| Calcium, mg/d | 4429 | 324.8 (209.9) | 321.8 (187.0) | 268.2 (249.3) | 56.6 (244.2) | 21 | 53.6 (249.6) | 20 |
| Iron, mg/d | 4429 | 11.4 (5.4) | 11.3 (4.4) | 9.6 (5.1) | 1.8 (4.6) | 19 | 1.7 (5.2) | 18 |
| Sodium, mg/d | 4429 | 5580.2 (2865.6) | 5527.2 (2379.4) | 4177.8 (3009.2) | 1402.4 (3506.3) | 34 | 1349.4 (3568.6) | 32 |
| Potassium, mg/d | 4429 | 1661.3 (739.2) | 1646.4 (592.3) | 1351.0 (618.0) | 310.4 (610.7) | 23 | 295.5 (693.3) | 22 |
| Magnesium, mg/d | 4429 | 365.3 (142.7) | 361.9 (105.2) | 315.8 (133.2) | 49.5 (111.9) | 16 | 46.2 (140.5) | 15 |
| Zinc, mg/d | 4429 | 9.5 (3.6) | 9.4 (2.7) | 8.5 (3.5) | 1.0 (2.8) | 12 | 0.9 (3.6) | 11 |

^1^ Dietary factors presented had adequate data/information for the present analysis.

^2^ Sample sizes differ because we performed paired analysis for each dietary factor, i.e., for each analysis we used only the individuals with available intake data for both diet assessments.

^3^ Bangladesh Integrated Household Survey (BIHS) 2011-2012 provided household-level dietary data from a 7-day household consumption questionnaire and individual-level data from 24-hour recalls (24hR). Household consumption was individualized by applying a) the Adult Male Equivalent (AME) method,^12^ as proposed by FAO^14^, assuming moderate physical activity, and b) the per capita (PC) approach assuming equal distribution among household members (Appendix B). Individual intake was estimated from 24hR.

^4^ Absolute differences correspond to AME-24hR and PC-24hR respectively, and percentage differences correspond to absolute difference/24hR*100.

^5^ Differences between means were significant for all dietary factors (paired t-test, P<0.05), with the exception of starchy vegetables (P=0.14), and legumes (P=0.07).

^6^ Differences between means were significant for all dietary factors (paired t-test, P<0.05), with the exception of legumes (P0.13), and seafood (P=0.14).

MUFA, Monounsaturated fats; PUFA, Polyunsaturated fats; SFA, Saturated fat

# Table R. Comparison of individualized household consumption and individual dietary intake estimates by dietary factor among individuals in the fourth quintile of household income in the 2011-2012 BIHS.

| **Dietary Factor, unit^1^** | **Observations (n)^2^** | **Consumption**  **(mean, SD)^3^** | | | **Difference between 24hR and AME (mean, SD)^4^** | | **Difference between 24hR and PC (mean, SD)^4^** | |
| --- | --- | --- | --- | --- | --- | --- | --- | --- |
|  |  | **AME^3^** | **PC^3^** | **24hR^3^** | **Absolute^5^** | **Percent** | **Absolute^6^** | **Percent** |
| **Food groups** |  |  |  |  |  |  |  |  |
| Fruits, g/d | 4416 | 28.2 (49.6) | 28.0 (47.5) | 8.2 (32) | 20.0 (50.7) | 244 | 19.8 (50.7) | 241 |
| Non-starchy vegetables, g/d | 4420 | 256.2 (160.2) | 254.8.0 (143.2) | 172.7 (142.3) | 83.6 (169.3) | 48 | 82.1 (173.2) | 48 |
| Starchy vegetables, g/d | 4340 | 107.9 (68.7) | 107.3 (61.6) | 107.6 (98.4) | 0.4 (94.4) | 0 | -0.3 (98.8) | 0 |
| Legumes, g/d | 3901 | 20.1 (27.0) | 20.0 (25.5) | 19.5 (49.5) | 0.7 (47.0) | 4 | 0.5 (47.3) | 3 |
| Total grains, g/d | 2,556 | 1493.6 (549.1) | 1484.1 (401.6) | 1396.6 (571.9) | 96.9 (427.3) | 7 | 87.5 (563.8) | 6 |
| Meat/Eggs, g/d | 4420 | 25.7 (24.2) | 25.5 (22.6) | 24.5 (29.3) | 4.8 (25.7) | 20 | 4.7 (25.6) | 19 |
| Seafood, g/d | 3024 | 16.3 (18.5) | 16.2 (17.5) | 11.4 (25.6) | 1.2 (27.9) | 11 | 1.0 (28.7) | 9 |
| Milk, whole fat, g/d | 4220 | 27.4 (58.4) | 27.3 (56.2) | 17.6 (68.1) | 9.8 (69.3) | 56 | 9.6 (65.4) | 55 |
| Fats/Oils, g/d | 4420 | 19.7 (12.5) | 19.6 (11.2) | 17.5 (15.7) | 2.2 (14.8) | 13 | 2.0 (15.4) | 11 |
|  |  |  |  |  |  |  |  |  |
| **Energy & Macronutrients** |  |  |  |  |  |  |  |  |
| Energy, kcal/d | 4420 | 2326.3 (870.5) | 2312.0 (641.3) | 2082.3 (817.4) | 243.9 (683.5) | 12 | 229.6 (854.1) | 11 |
| Protein, g/d | 4420 | 56.3 (23.2) | 56.0 (18.2) | 50.2 (21.8) | 6.2 (19.6) | 12 | 5.8 (23.1) | 12 |
| Carbohydrates, g/d | 4420 | 446.7 (165.9) | 444.0 (121.4) | 402.8 (158.9) | 43.9 (131.3) | 11 | 41.2 (165.9) | 10 |
| Total fat, g/d | 4420 | 29.3 (17.3) | 29.1 (15.3) | 25.2 (19.1) | 4.1 (17.8) | 16 | 3.9 (18.4) | 15 |
| SFA, g/d | 4420 | 6.4 (5.0) | 6.4 (4.5) | 5.2 (4.5) | 1.2 (5.1) | 23 | 1.1 (5.0) | 21 |
| MUFA, g/d | 4420 | 9.1 (5.4) | 9.0 (4.8) | 7.7 (5.9) | 1.3 (5.2) | 17 | 1.3 (5.5) | 17 |
| PUFA, g/d | 4420 | 13.3 (8.4) | 13.2 (7.6) | 12.0 (10.1) | 1.3 (9.0) | 11 | 1.3 (9.5) | 11 |
| Cholesterol, mg/d | 4420 | 42.0 (41.3) | 41.8 (38.8) | 33.0 (56.4) | 9.1 (54.4) | 28 | 8.8 (53.9) | 27 |
| Fiber, g/d | 4420 | 31.2 (12.6) | 31.0 (9.7) | 26.4 (11.4) | 4.9 (10.5) | 19 | 4.7 (12.5) | 18 |
|  |  |  |  |  |  |  |  |  |
| **Vitamins** |  |  |  |  |  |  |  |  |
| Vitamin A, μg RAE/d | 4420 | 311.0 (327.2) | 309.3 (307.5) | 215.6 (407.4) | 95.5 (432.5) | 44 | 93.8 (432.2) | 44 |
| Vitamin D, μg/d | 4420 | 1.4 (2.0) | 1.4 (1.9) | 1.1 (2.6) | 0.3 (2.6) | 27 | 0.3 (2.6) | 27 |
| Vitamin E, mg/d | 4420 | 5.2 (3.0) | 5.2 (2.6) | 4.4 (3.3) | 0.8 (2.9) | 18 | 0.8 (3.1) | 18 |
| Thiamine, mg/d | 4420 | 0.9 (0.4) | 0.9 (0.3) | 0.8 (0.3) | 0.1 (0.3) | 13 | 0.1 (0.4) | 13 |
| Riboflavin, mg/d | 4420 | 0.6 (0.3) | 0.6 (0.2) | 0.5 (0.2) | 0.1 (0.3) | 20 | 0.1 (0.3) | 20 |
| Niacin, mg/d | 4420 | 16.3 (6.5) | 16.2 (4.9) | 14.4 (6.2) | 1.9 (4.9) | 13 | 1.7 (6.1) | 12 |
| Vitamin B6, mg/d | 4420 | 1.3 (0.7) | 1.3 (0.6) | 1.2 (0.6) | 0.2 (0.5) | 17 | 0.2 (0.6) | 17 |
| Folate, μg/d | 4420 | 155.8 (83.3) | 154.8 (71.9) | 123.1 (80.9) | 32.7 (89.4) | 27 | 31.7 (92.5) | 26 |
| Vitamin C, mg/d | 4420 | 64.4 (44.3) | 64.1 (40.0) | 43.1 (37.6) | 21.4 (47.3) | 50 | 21.0 (47.5) | 49 |
|  |  |  |  |  |  |  |  |  |
| **Minerals** |  |  |  |  |  |  |  |  |
| Calcium, mg/d | 4420 | 341.9 (204.1) | 339.9 (180.1) | 274.5 (191.4) | 67.5 (199.1) | 25 | 65.4 (202.9) | 24 |
| Iron, mg/d | 4420 | 11.8 (5.5) | 11.8 (4.5) | 9.9 (5.1) | 1.9 (4.9) | 19 | 1.8 (5.4) | 18 |
| Sodium, mg/d | 4420 | 5855.7 (3096.8) | 5823.1 (2669.2) | 4236.4 (3116.9) | 1619.3 (3698.3) | 38 | 1586.7 (3768.9) | 37 |
| Potassium, mg/d | 4420 | 1732.3 (767.4) | 1721.6 (621.1) | 1413.9 (627.5) | 318.5 (655.9) | 23 | 307.8 (728.7) | 22 |
| Magnesium, mg/d | 4420 | 377.8 (146.6) | 375.5 (110.7) | 324.0 (132.1) | 53.8 (118.7) | 17 | 51.5 (143.7) | 16 |
| Zinc, mg/d | 4420 | 9.8 (3.8) | 9.8 (2.8) | 8.7 (3.5) | 1.1 (3.1) | 13 | 1.1 (3.7) | 13 |

^1^ Dietary factors presented had adequate data/information for the present analysis.

^2^ Sample sizes differ because we performed paired analysis for each dietary factor, i.e., for each analysis we used only the individuals with available intake data for both diet assessments.

^3^ Bangladesh Integrated Household Survey (BIHS) 2011-2012 provided household-level dietary data from a 7-day household consumption questionnaire and individual-level data from 24-hour recalls (24hR). Household consumption was individualized by applying a) the Adult Male Equivalent (AME) method,^12^ as proposed by FAO^14^, assuming moderate physical activity, and b) the per capita (PC) approach assuming equal distribution among household members (Appendix B). Individual intake was estimated from 24hR.

^4^ Absolute differences correspond to AME-24hR and PC-24hR respectively, and percentage differences correspond to absolute difference/24hR*100.

^5^ Differences between means were significant for all dietary factors (paired t-test, P<0.01), with the exception of starchy vegetables (P=0.80), and legumes (P=0.38).

^6^ Differences between means were significant for all dietary factors (paired t-test, P<0.05), with the exception of starchy vegetables (P=0.85), and legumes (P=0.48).

MUFA, Monounsaturated fats; PUFA, Polyunsaturated fats; SFA, Saturated fat

# Table S. Comparison of individualized household consumption and individual dietary intake estimates by dietary factor among individuals in the fifth quintile of household income in the 2011-2012 BIHS.

| **Dietary Factor, unit^1^** | **Observations (n)^2^** | **Consumption**  **(mean, SD)^3^** | | | **Difference between 24hR and AME (mean, SD)^4^** | | **Difference between 24hR and PC (mean, SD)^4^** | |
| --- | --- | --- | --- | --- | --- | --- | --- | --- |
|  |  | **AME^3^** | **PC^3^** | **24hR^3^** | **Absolute^5^** | **Percent** | **Absolute^6^** | **Percent** |
| **Food groups** |  |  |  |  |  |  |  |  |
| Fruits, g/d | 4425 | 38.4 (57.3) | 38.1 (53.9) | 11.8 (39.9) | 26.6 (60.5) | 225 | 26.3 (57.1) | 223 |
| Non-starchy vegetables, g/d | 4433 | 282.9 (173) | 281.2 (156.1) | 179.5 (163.7) | 103.4 (191.0) | 58 | 101.7 (192.4) | 57 |
| Starchy vegetables, g/d | 4375 | 101.0 (62.3) | 100.4 (56.1) | 111.1 (103.9) | -10.1 (96.9) | -9 | -10.7 (100.0) | -10 |
| Legumes, g/d | 3971 | 27.1 (36.2) | 27.0 (34.8) | 23.1 (51.9) | 4.0 (53.6) | 17 | 3.9 (53.9) | 17 |
| Total grains, g/d | 2310 | 1508.4 (532.2) | 1499.6 (404.4) | 1418.7 (562.4) | 89.7 (444.8) | 6 | 80.9 (565.8) | 6 |
| Meat/Eggs, g/d | 4433 | 31.1 (27.6) | 30.9 (25.8) | 29.6 (34.2) | 6.6 (30.4) | 22 | 6.5 (30.3) | 22 |
| Seafood, g/d | 3129 | 21.7 (21.4) | 21.6 (20.3) | 15.0 (28.0) | 1.5 (32.1) | 10 | 1.2 (32.8) | 8 |
| Milk, whole fat, g/d | 4194 | 37.5 (63.5) | 37.3 (60.6) | 26.8 (72.6) | 10.8 (65.3) | 40 | 10.5 (60.9) | 39 |
| Fats/Oils, g/d/ | 4433 | 23.0 (13.9) | 22.8 (12.5) | 20.5 (17.1) | 2.5 (16.5) | 12 | 2.3 (17.0) | 11 |
|  |  |  |  |  |  |  |  |  |
| **Energy & Macronutrients** |  |  |  |  |  |  |  |  |
| Energy, kcal/d | 4433 | 2445.9 (881.3) | 2431.4 (661.7) | 2202.1 (833.4) | 243.7 (711.6) | 11 | 229.2 (866.5) | 10 |
| Protein, g/d | 4433 | 60.7 (24.6) | 60.4 (19.8) | 54.2 (22.6) | 6.5 (21.2) | 12 | 6.2 (24.3) | 11 |
| Carbohydrates, g/d | 4433 | 459.9 (164.1) | 457.2 (122.6) | 417.8 (159.6) | 42.0 (132.7) | 10 | 39.4 (164.5) | 9 |
| Total fat, g/d | 4433 | 34.5 (20.3) | 34.3 (18.1) | 29.8 (21.4) | 4.8 (21.1) | 16 | 4.5 (21.6) | 15 |
| SFA, g/d | 4433 | 7.8 (6.8) | 7.7 (6.3) | 6.4 (5.9) | 1.4 (7.2) | 22 | 1.4 (7.1) | 22 |
| MUFA, g/d | 4433 | 10.2 (5.8) | 10.2 (5.1) | 8.7 (6.4) | 1.5 (6.0) | 17 | 1.4 (6.2) | 16 |
| PUFA, g/d | 4433 | 15.6 (9.4) | 15.5 (8.4) | 14.1 (10.9) | 1.5 (10.1) | 11 | 1.4 (10.5) | 10 |
| Cholesterol, mg/d | 4433 | 55.6 (53.6) | 55.3 (50.7) | 42.6 (63.7) | 13.0 (67.1) | 31 | 12.7 (66.8) | 30 |
| Fiber, g/d | 4433 | 33.0 (13.1) | 32.8 (10.4) | 27.6 (11.7) | 5.4 (11.2) | 20 | 5.2 (12.8) | 19 |
|  |  |  |  |  |  |  |  |  |
| **Vitamins** |  |  |  |  |  |  |  |  |
| Vitamin A, μg RAE/d | 4433 | 363.2 (374.2) | 361.0 (353.6) | 227.7 (404.6) | 135.5 (442.0) | 60 | 133.3 (441.9) | 59 |
| Vitamin D, μg/d | 4433 | 1.6 (2.1) | 1.6 (1.9) | 1.3 (2.8) | 0.3 (2.7) | 23 | 0.3 (2.7) | 23 |
| Vitamin E, mg/d | 4433 | 6.0 (3.3) | 6.0 (2.9) | 5.1 (3.6) | 0.9 (3.2) | 18 | 0.9 (3.4) | 18 |
| Thiamine, mg/d | 4433 | 1.0 (0.4) | 1.0 (0.3) | 0.8 (0.4) | 0.2 (0.4) | 25 | 0.2 (0.4) | 25 |
| Riboflavin, mg/d | 4433 | 0.6 (0.3) | 0.6 (0.3) | 0.5 (0.3) | 0.1 (0.3) | 20 | 0.1 (0.3) | 20 |
| Niacin, mg/d | 4433 | 16.6 (6.5) | 16.5 (5.1) | 14.8 (6.2) | 1.8 (5.0) | 12 | 1.7 (6.1) | 11 |
| Vitamin B6, mg/d | 4433 | 1.4 (0.7) | 1.4 (0.6) | 1.3 (0.7) | 0.2 (0.5) | 15 | 0.2 (0.6) | 15 |
| Folate, μg/d | 4433 | 170.5 (84.4) | 169.4 (72.8) | 132.0 (81.4) | 38.4 (87.6) | 29 | 37.4 (90.1) | 28 |
| Vitamin C, mg/d | 4433 | 72.6 (48.1) | 72.1 (43.9) | 47.3 (47.4) | 25.3 (56.8) | 53 | 24.8 (56.6) | 52 |
|  |  |  |  |  |  |  |  |  |
| **Minerals** |  |  |  |  |  |  |  |  |
| Calcium, mg/d | 4433 | 385.9 (216.9) | 383.4 (192.2) | 305.6 (222.0) | 80.3 (212.8) | 26 | 77.8 (215.5) | 25 |
| Iron, mg/d | 4433 | 12.7 (5.8) | 12.6 (4.9) | 10.6 (5.2) | 2.2 (5.1) | 21 | 2.1 (5.6) | 20 |
| Sodium, mg/d | 4433 | 6017.2 (3155.7) | 5986.8 (2763.5) | 4247.8 (2568.2) | 1769.4 (3341.1) | 42 | 1739.0 (3389.8) | 41 |
| Potassium, mg/d | 4433 | 1861.0 (807.4) | 1849.4 (665.0) | 1505.2 (661.8) | 355.8 (696.5) | 24 | 344.2 (751.0) | 23 |
| Magnesium, mg/d | 4433 | 394.8 (148.2) | 392.5 (114.1) | 339.2 (134.1) | 55.6 (120.8) | 16 | 53.3 (143.2) | 16 |
| Zinc, mg/d | 4433 | 10.3 (3.8) | 10.2 (3.0) | 9.2 (3.6) | 1.1 (3.2) | 12 | 1.1 (3.8) | 12 |

^1^ Dietary factors presented had adequate data/information for the present analysis.

^2^ Sample sizes differ because we performed paired analysis for each dietary factor, i.e., for each analysis we used only the individuals with available intake data for both diet assessments.

^3^ Bangladesh Integrated Household Survey (BIHS) 2011-2012 provided household-level dietary data from a 7-day household consumption questionnaire and individual-level data from 24-hour recalls (24hR). Household consumption was individualized by applying a) the Adult Male Equivalent (AME) method,^12^ as proposed by FAO^14^, assuming moderate physical activity, and b) the per capita (PC) approach assuming equal distribution among household members (Appendix B). Individual intake was estimated from 24hR.

^4^ Absolute differences correspond to AME-24hR and PC-24hR respectively, and percentage differences correspond to absolute difference/24hR*100.

^5^ Differences between means were significant for all dietary factors (paired t-test, P<0.01).

^6^ Differences between means were significant for all dietary factors (paired t-test, P<0.05).

MUFA, Monounsaturated fats; PUFA, Polyunsaturated fats; SFA, Saturated fat

# Table T. Relation between individualized household intake estimates as predictors of individual dietary intakes by sex in the 2011-2012 BIHS.

|  | **Men** | | **Women** | |
| --- | --- | --- | --- | --- |
| **Dietary Factor, unit** | ***β,* AME (SE)** | **R** | ***β,* AME (SE)** | **R** |
| **Food groups** |  |  |  |  |
| Fruits, g/d |  |  |  |  |
| Unadjusted | 0.1 (0.02) | 0.06 | 0.2 (0.02) | 0.07 |
| Sex and age | 0.1 (0.02) | 0.06 | 0.2 (0.02) | 0.07 |
| Multivariate | 0.1 (0.02) | 0.06 | 0.2 (0.02) | 0.07 |
| Non-starchy vegetables, g/d |  |  |  |  |
| Unadjusted | 0.5 (0.01) | 0.16 | 0.5 (0.01) | 0.14 |
| Sex and age | 0.3 (0.02) | 0.20 | 0.3 (0.02) | 0.17 |
| Multivariate | 0.3 (0.02) | 0.21 | 0.3 (0.02) | 0.19 |
| Starchy vegetables, g/d |  |  |  |  |
| Unadjusted | 0.8 (0.03) | 0.17 | 0.7 (0.02) | 0.13 |
| Sex and age | 0.5 (0.03) | 0.20 | 0.5 (0.03) | 0.15 |
| Multivariate | 0.5 (0.03) | 0.20 | 0.5 (0.03) | 0.16 |
| Legumes, g/d |  |  |  |  |
| Unadjusted | 0.6 (0.04) | 0.13 | 0.6 (0.04) | 0.13 |
| Sex and age | 0.5 (0.04) | 0.13 | 0.5 (0.04) | 0.14 |
| Multivariate | 0.5 (0.04) | 0.13 | 0.6 (0.04) | 0.14 |
| Total grains, g/d |  |  |  |  |
| Unadjusted | 0.8 (0.01) | 0.51 | 0.8 (0.01) | 0.43 |
| Sex and age | 0.5 (0.02) | 0.60 | 0.5 (0.02) | 0.53 |
| Multivariate | 0.6 (0.02) | 0.63 | 0.5 (0.02) | 0.56 |
| Meat/Eggs, g/d |  |  |  |  |
| Unadjusted | 0.5 (0.04) | 0.13 | 0.4 (0.04) | 0.12 |
| Sex and age | 0.5 (0.04) | 0.13 | 0.4 (0.04) | 0.12 |
| Multivariate | 0.4 (0.04) | 0.15 | 0.4 (0.04) | 0.14 |
| Seafood, g/d |  |  |  |  |
| Unadjusted | 0.7 (0.02) | 0.22 | 0.6 (0.03) | 0.21 |
| Sex and age | 0.5 (0.03) | 0.24 | 0.5 (0.03) | 0.22 |
| Multivariate | 0.5 (0.02) | 0.25 | 0.5 (0.03) | 0.22 |
| Milk, whole fat, g/d |  |  |  |  |
| Unadjusted | 0.5 (0.03) | 0.28 | 0.5 (0.03) | 0.20 |
| Sex and age | 0.5 (0.03) | 0.29 | 0.5 (0.03) | 0.22 |
| Multivariate | 0.5 (0.03) | 0.30 | 0.5 (0.03) | 0.22 |
| Fats/Oils, g/d |  |  |  |  |
| Unadjusted | 0.7 (0.02) | 0.24 | 0.7 (0.02) | 0.21 |
| Sex and age | 0.5 (0.02) | 0.26 | 0.5 (0.02) | 0.22 |
| Multivariate | 0.5 (0.02) | 0.26 | 0.5 (0.03) | 0.23 |
|  |  |  |  |  |
| **Energy & Macronutrients** |  |  |  |  |
| Energy, kcal/d |  |  |  |  |
| Unadjusted | 0.8 (0.01) | 0.49 | 0.7 (0.01) | 0.41 |
| Sex and age | 0.4 (0.01) | 0.58 | 0.4 (0.01) | 0.50 |
| Multivariate | 0.5 (0.01) | 0.60 | 0.4 (0.01) | 0.53 |
| Protein, g/d |  |  |  |  |
| Unadjusted | 0.7 (0.01) | 0.42 | 0.7 (0.01) | 0.35 |
| Sex and age | 0.4 (0.01) | 0.50 | 0.4 (0.01) | 0.42 |
| Multivariate | 0.5 (0.01) | 0.51 | 0.4 (0.01) | 0.45 |
| Carbohydrates, g/d |  |  |  |  |
| Unadjusted | 0.8 (0.01) | 0.49 | 0.7 (0.01) | 0.40 |
| Sex and age | 0.4 (0.01) | 0.58 | 0.4 (0.01) | 0.51 |
| Multivariate | 0.5 (0.01) | 0.60 | 0.4 (0.01) | 0.54 |
| Total fat, g/d |  |  |  |  |
| Unadjusted | 0.6 (0.02) | 0.29 | 0.6 (0.02) | 0.25 |
| Sex and age | 0.5 (0.02) | 0.31 | 0.5 (0.02) | 0.26 |
| Multivariate | 0.5 (0.02) | 0.31 | 0.5 (0.02) | 0.27 |
| SFA, g/d |  |  |  |  |
| Unadjusted | 0.4 (0.04) | 0.20 | 0.4 (0.04) | 0.17 |
| Sex and age | 0.3 (0.04) | 0.21 | 0.3 (0.04) | 0.18 |
| Multivariate | 0.3 (0.04) | 0.22 | 0.3 (0.04) | 0.19 |
| MUFA, g/d |  |  |  |  |
| Unadjusted | 0.6 (0.02) | 0.32 | 0.6 (0.02) | 0.27 |
| Sex and age | 0.5 (0.03) | 0.32 | 0.5 (0.02) | 0.28 |
| Multivariate | 0.5 (0.03) | 0.33 | 0.5 (0.02) | 0.28 |
| PUFA, g/d |  |  |  |  |
| Unadjusted | 0.7 (0.02) | 0.30 | 0.7 (0.02) | 0.27 |
| Sex and age | 0.6 (0.02) | 0.32 | 0.6 (0.02) | 0.28 |
| Multivariate | 0.5 (0.02) | 0.33 | 0.6 (0.02) | 0.29 |
| Cholesterol, mg/d |  |  |  |  |
| Unadjusted | 0.5 (0.03) | 0.15 | 0.3 (0.03) | 0.13 |
| Sex and age | 0.5 (0.03) | 0.15 | 0.4 (0.03) | 0.13 |
| Multivariate | 0.4 (0.03) | 0.16 | 0.3 (0.03) | 0.13 |
| Fiber, g/d |  |  |  |  |
| Unadjusted | 0.7 (0.01) | 0.40 | 0.7 (0.01) | 0.33 |
| Sex and age | 0.4 (0.01) | 0.51 | 0.3 (0.01) | 0.44 |
| Multivariate | 0.4 (0.01) | 0.52 | 0.4 (0.01) | 0.46 |
|  |  |  |  |  |
| **Vitamins** |  |  |  |  |
| Vitamin A, μg RAE/d |  |  |  |  |
| Unadjusted | 0.4 (0.03) | 0.12 | 0.4 (0.03) | 0.11 |
| Sex and age | 0.4 (0.03) | 0.12 | 0.4 (0.03) | 0.11 |
| Multivariate | 0.4 (0.03) | 0.13 | 0.4 (0.03) | 0.11 |
| Vitamin D, μg/d |  |  |  |  |
| Unadjusted | 0.6 (0.04) | 0.19 | 0.6 (0.04) | 0.17 |
| Sex and age | 0.6 (0.04) | 0.19 | 0.5 (0.04) | 0.18 |
| Multivariate | 0.6 (0.04) | 0.19 | 0.5 (0.04) | 0.18 |
| Vitamin E, mg/d |  |  |  |  |
| Unadjusted | 0.7 (0.01) | 0.33 | 0.7 (0.01) | 0.30 |
| Sex and age | 0.5 (0.02) | 0.35 | 0.5 (0.02) | 0.32 |
| Multivariate | 0.5 (0.02) | 0.36 | 0.5 (0.02) | 0.32 |
| Thiamine, mg/d |  |  |  |  |
| Unadjusted | 0.7 (0.01) | 0.36 | 0.6 (0.01) | 0.30 |
| Sex and age | 0.4 (0.01) | 0.45 | 0.4 (0.01) | 0.38 |
| Multivariate | 0.4 (0.01) | 0.46 | 0.4 (0.01) | 0.39 |
| Riboflavin, mg/d |  |  |  |  |
| Unadjusted | 0.5 (0.01) | 0.33 | 0.5 (0.01) | 0.24 |
| Sex and age | 0.4 (0.02) | 0.37 | 0.3 (0.01) | 0.27 |
| Multivariate | 0.4 (0.02) | 0.38 | 0.3 (0.02) | 0.29 |
| Niacin, mg/d |  |  |  |  |
| Unadjusted | 0.8 (0.01) | 0.52 | 0.8 (0.01) | 0.46 |
| Sex and age | 0.5 (0.01) | 0.58 | 0.5 (0.01) | 0.53 |
| Multivariate | 0.6 (0.01) | 0.60 | 0.5 (0.01) | 0.55 |
| Vitamin B6, mg/d |  |  |  |  |
| Unadjusted | 0.8 (0.01) | 0.51 | 0.7 (0.01) | 0.46 |
| Sex and age | 0.6 (0.02) | 0.54 | 0.6 (0.02) | 0.49 |
| Multivariate | 0.6 (0.02) | 0.57 | 0.6 (0.02) | 0.53 |
| Folate, μg/d |  |  |  |  |
| Unadjusted | 0.6 (0.01) | 0.22 | 0.5 (0.01) | 0.17 |
| Sex and age | 0.3 (0.02) | 0.26 | 0.3 (0.02) | 0.21 |
| Multivariate | 0.3 (0.02) | 0.28 | 0.3 (0.02) | 0.22 |
| Vitamin C, mg/d |  |  |  |  |
| Unadjusted | 0.4 (0.02) | 0.11 | 0.4 (0.02) | 0.11 |
| Sex and age | 0.2 (0.02) | 0.14 | 0.3 (0.02) | 0.13 |
| Multivariate | 0.3 (0.02) | 0.15 | 0.3 (0.02) | 0.14 |
|  |  |  |  |  |
| **Minerals** |  |  |  |  |
| Calcium, mg/d |  |  |  |  |
| Unadjusted | 0.6 (0.02) | 0.24 | 0.5 (0.02) | 0.19 |
| Sex and age | 0.4 (0.03) | 0.25 | 0.4 (0.02) | 0.19 |
| Multivariate | 0.5 (0.03) | 0.26 | 0.4 (0.02) | 0.20 |
| Iron, mg/d |  |  |  |  |
| Unadjusted | 0.7 (0.01) | 0.34 | 0.6 (0.01) | 0.29 |
| Sex and age | 0.4 (0.02) | 0.40 | 0.4 (0.01) | 0.35 |
| Multivariate | 0.4 (0.02) | 0.41 | 0.4 (0.01) | 0.37 |
| Sodium, mg/d |  |  |  |  |
| Unadjusted | 0.5 (0.01) | 0.10 | 0.4 (0.01) | 0.06 |
| Sex and age | 0.2 (0.02) | 0.18 | 0.2 (0.01) | 0.10 |
| Multivariate | 0.2 (0.02) | 0.19 | 0.2 (0.01) | 0.11 |
| Potassium, mg/d |  |  |  |  |
| Unadjusted | 0.6 (0.01) | 0.35 | 0.5 (0.01) | 0.29 |
| Sex and age | 0.3 (0.01) | 0.44 | 0.3 (0.01) | 0.38 |
| Multivariate | 0.3 (0.01) | 0.45 | 0.3 (0.01) | 0.40 |
| Magnesium, mg/d |  |  |  |  |
| Unadjusted | 0.7 (0.01) | 0.44 | 0.7 (0.01) | 0.36 |
| Sex and age | 0.4 (0.01) | 0.55 | 0.3 (0.01) | 0.47 |
| Multivariate | 0.4 (0.01) | 0.56 | 0.4 (0.01) | 0.50 |
| Zinc, mg/d |  |  |  |  |
| Unadjusted | 0.7 (0.01) | 0.46 | 0.7 (0.01) | 0.38 |
| Sex and age | 0.4 (0.01) | 0.55 | 0.4 (0.01) | 0.48 |
| Multivariate | 0.5 (0.01) | 0.57 | 0.4 (0.01) | 0.50 |

All p-values are <0.001.

On the basis of linear regression models with 24-hour diet recall intakes (24hR) as the dependent variable and individualized Adult Male Equivalent (AME) or per capita (PC) consumption estimates from household questionnaire as the independent variable. The sex and age model was categorized as follows: age, ≤5, 6-10, 11-19, 20-44, and ≥45 years; sex was not considered a covariate for this analysis. The multivariate model was adjusted for the following covariates: age (≤5, 6-10, 11-19, 20-44, and ≥45 years), education (<6 years of education, ≥6 years of education), religion (Muslims, other), household income (quintiles), respondent’s age (continuous), sex (men, women) and education (<6 years of education, ≥6 years of education), household size, number of children within household, and food wastage percentage (using 24hR data, we calculated for each household, the percent of food wastage -sum of food waste, and food given to guests, others and animals- to total consumed food (mean: 11.6%, SD: 13.6)). *Βs* represent the change in the individual intake (24hR) for every unit increase in the AME mean. SEs for *βs* are presented. R^2^ represents the coefficient of determination for the overall model.

BIHS, Bangladesh Integrated Household Survey; MUFA, Monounsaturated fats; PUFA, Polyunsaturated fats; SFA, Saturated fat

# Table U. Relation between individualized household intake estimates as predictors of individual dietary intakes by age in the 2011-2012 BIHS.

|  | **0-5** | | **6-10** | | **11-19** | | **20-44** | | **≥45** | |
| --- | --- | --- | --- | --- | --- | --- | --- | --- | --- | --- |
| **Dietary Factor, unit** | ***β,* AME (SE)** | **R** | ***β,* AME (SE)** | **R** | ***β,* AME (SE)** | **R** | ***β,* AME (SE)** | **R** | ***β,* AME (SE)** | **R** |
| **Food groups** |  |  |  |  |  |  |  |  |  |  |
| Fruits, g/d |  |  |  |  |  |  |  |  |  |  |
| Unadjusted | 0.3 (0.06) | 0.09 | 0.2 (0.04) | 0.06 | 0.1 (0.02) | 0.06 | 0.2 (0.02) | 0.07 | 0.1 (0.02) | 0.06 |
| Sex and age | 0.3 (0.06) | 0.09 | 0.2 (0.04) | 0.06 | 0.1 (0.02) | 0.06 | 0.2 (0.02) | 0.07 | 0.1 (0.02) | 0.06 |
| Multivariate | 0.3 (0.06) | 0.09 | 0.2 (0.04) | 0.08 | 0.1 (0.02) | 0.06 | 0.2 (0.02) | 0.07 | 0.1 (0.02) | 0.06 |
| Non-starchy vegetables, g/d |  |  |  |  |  |  |  |  |  |  |
| Unadjusted | 0.4 (0.04) | 0.10 | 0.3 (0.02) | 0.06 | 0.3 (0.02) | 0.11 | 0.3 (0.02) | 0.10 | 0.3 (0.02) | 0.10 |
| Sex and age | 0.4 (0.04) | 0.10 | 0.3 (0.02) | 0.06 | 0.3 (0.02) | 0.11 | 0.2 (0.02) | 0.10 | 0.3 (0.02) | 0.10 |
| Multivariate | 0.4 (0.04) | 0.13 | 0.3 (0.02) | 0.08 | 0.3 (0.02) | 0.12 | 0.3 (0.02) | 0.11 | 0.3 (0.02) | 0.11 |
| Starchy vegetables, g/d |  |  |  |  |  |  |  |  |  |  |
| Unadjusted | 0.5 (0.04) | 0.10 | 0.5 (0.04) | 0.09 | 0.4 (0.04) | 0.09 | 0.5 (0.03) | 0.12 | 0.5 (0.03) | 0.10 |
| Sex and age | 0.5 (0.04) | 0.10 | 0.5 (0.04) | 0.09 | 0.4 (0.04) | 0.09 | 0.5 (0.03) | 0.12 | 0.4 (0.03) | 0.11 |
| Multivariate | 0.5 (0.04) | 0.12 | 0.5 (0.04) | 0.10 | 0.4 (0.04) | 0.10 | 0.5 (0.03) | 0.13 | 0.5 (0.04) | 0.12 |
| Legumes, g/d |  |  |  |  |  |  |  |  |  |  |
| Unadjusted | 0.7 (0.09) | 0.13 | 0.6 (0.06) | 0.13 | 0.5 (0.06) | 0.14 | 0.5 (0.04) | 0.12 | 0.5 (0.06) | 0.12 |
| Sex and age | 0.7 (0.09) | 0.13 | 0.6 (0.06) | 0.13 | 0.5 (0.06) | 0.14 | 0.5 (0.04) | 0.12 | 0.5 (0.06) | 0.12 |
| Multivariate | 0.7 (0.09) | 0.14 | 0.6 (0.06) | 0.14 | 0.5 (0.06) | 0.14 | 0.5 (0.04) | 0.12 | 0.5 (0.06) | 0.13 |
| Total grains, g/d |  |  |  |  |  |  |  |  |  |  |
| Unadjusted | 0.9 (0.03) | 0.38 | 0.5 (0.03) | 0.21 | 0.5 (0.02) | 0.29 | 0.5 (0.02) | 0.24 | 0.5 (0.02) | 0.21 |
| Sex and age | 1 (0.03) | 0.39 | 0.5 (0.03) | 0.21 | 0.5 (0.02) | 0.29 | 0.4 (0.02) | 0.25 | 0.4 (0.02) | 0.22 |
| Multivariate | 1 (0.03) | 0.44 | 0.6 (0.03) | 0.30 | 0.5 (0.02) | 0.34 | 0.5 (0.02) | 0.30 | 0.5 (0.02) | 0.28 |
| Meat/Eggs, g/d |  |  |  |  |  |  |  |  |  |  |
| Unadjusted | 0.7 (0.09) | 0.13 | 0.5 (0.06) | 0.13 | 0.4 (0.05) | 0.14 | 0.4 (0.03) | 0.12 | 0.5 (0.06) | 0.14 |
| Sex and age | 0.7 (0.09) | 0.13 | 0.5 (0.06) | 0.14 | 0.4 (0.05) | 0.14 | 0.4 (0.03) | 0.12 | 0.5 (0.06) | 0.14 |
| Multivariate | 0.6 (0.09) | 0.14 | 0.5 (0.06) | 0.16 | 0.4 (0.05) | 0.16 | 0.4 (0.03) | 0.14 | 0.5 (0.06) | 0.15 |
| Seafood, g/d |  |  |  |  |  |  |  |  |  |  |
| Unadjusted | 0.6 (0.05) | 0.15 | 0.6 (0.05) | 0.19 | 0.5 (0.03) | 0.20 | 0.5 (0.02) | 0.19 | 0.5 (0.03) | 0.18 |
| Sex and age | 0.6 (0.05) | 0.15 | 0.6 (0.05) | 0.19 | 0.5 (0.04) | 0.20 | 0.5 (0.03) | 0.20 | 0.5 (0.03) | 0.18 |
| Multivariate | 0.6 (0.05) | 0.16 | 0.6 (0.05) | 0.19 | 0.5 (0.04) | 0.20 | 0.5 (0.03) | 0.20 | 0.5 (0.03) | 0.20 |
| Milk, whole fat, g/d |  |  |  |  |  |  |  |  |  |  |
| Unadjusted | 1.8 (0.15) | 0.28 | 0.7 (0.06) | 0.18 | 0.5 (0.04) | 0.31 | 0.5 (0.03) | 0.31 | 0.6 (0.04) | 0.35 |
| Sex and age | 1.8 (0.15) | 0.28 | 0.7 (0.06) | 0.18 | 0.5 (0.04) | 0.31 | 0.5 (0.03) | 0.31 | 0.6 (0.04) | 0.35 |
| Multivariate | 1.7 (0.15) | 0.30 | 0.7 (0.06) | 0.18 | 0.5 (0.04) | 0.32 | 0.5 (0.03) | 0.32 | 0.6 (0.04) | 0.36 |
| Fats/Oils, g/d |  |  |  |  |  |  |  |  |  |  |
| Unadjusted | 0.7 (0.04) | 0.21 | 0.5 (0.04) | 0.14 | 0.5 (0.04) | 0.18 | 0.5 (0.02) | 0.17 | 0.5 (0.03) | 0.17 |
| Sex and age | 0.7 (0.04) | 0.21 | 0.5 (0.04) | 0.14 | 0.5 (0.04) | 0.18 | 0.5 (0.02) | 0.17 | 0.5 (0.03) | 0.17 |
| Multivariate | 0.7 (0.05) | 0.21 | 0.5 (0.04) | 0.15 | 0.5 (0.04) | 0.19 | 0.4 (0.03) | 0.19 | 0.5 (0.03) | 0.18 |
|  |  |  |  |  |  |  |  |  |  |  |
| **Energy & Macronutrients** |  |  |  |  |  |  |  |  |  |  |
| Energy, kcal/d |  |  |  |  |  |  |  |  |  |  |
| Unadjusted | 0.8 (0.03) | 0.34 | 0.5 (0.02) | 0.20 | 0.4 (0.02) | 0.24 | 0.4 (0.01) | 0.21 | 0.4 (0.02) | 0.20 |
| Sex and age | 0.8 (0.03) | 0.34 | 0.5 (0.02) | 0.20 | 0.4 (0.02) | 0.24 | 0.3 (0.01) | 0.23 | 0.4 (0.02) | 0.23 |
| Multivariate | 0.9 (0.03) | 0.37 | 0.5 (0.02) | 0.24 | 0.4 (0.02) | 0.28 | 0.4 (0.01) | 0.26 | 0.4 (0.02) | 0.27 |
| Protein, g/d |  |  |  |  |  |  |  |  |  |  |
| Unadjusted | 0.7 (0.03) | 0.29 | 0.5 (0.02) | 0.20 | 0.4 (0.02) | 0.23 | 0.4 (0.01) | 0.19 | 0.5 (0.02) | 0.20 |
| Sex and age | 0.8 (0.03) | 0.29 | 0.5 (0.02) | 0.20 | 0.4 (0.02) | 0.23 | 0.4 (0.01) | 0.21 | 0.4 (0.02) | 0.22 |
| Multivariate | 0.8 (0.03) | 0.32 | 0.5 (0.02) | 0.23 | 0.4 (0.02) | 0.27 | 0.4 (0.01) | 0.24 | 0.4 (0.02) | 0.26 |
| Carbohydrates, g/d |  |  |  |  |  |  |  |  |  |  |
| Unadjusted | 0.8 (0.03) | 0.33 | 0.5 (0.02) | 0.20 | 0.4 (0.02) | 0.24 | 0.4 (0.01) | 0.20 | 0.4 (0.02) | 0.19 |
| Sex and age | 0.8 (0.03) | 0.34 | 0.5 (0.02) | 0.20 | 0.4 (0.02) | 0.23 | 0.3 (0.01) | 0.22 | 0.4 (0.02) | 0.22 |
| Multivariate | 0.9 (0.03) | 0.37 | 0.5 (0.02) | 0.25 | 0.5 (0.02) | 0.28 | 0.4 (0.01) | 0.27 | 0.4 (0.02) | 0.27 |
| Total fat, g/d |  |  |  |  |  |  |  |  |  |  |
| Unadjusted | 0.7 (0.05) | 0.24 | 0.5 (0.03) | 0.20 | 0.5 (0.03) | 0.23 | 0.5 (0.02) | 0.22 | 0.5 (0.03) | 0.21 |
| Sex and age | 0.7 (0.05) | 0.24 | 0.5 (0.03) | 0.20 | 0.5 (0.03) | 0.23 | 0.5 (0.02) | 0.22 | 0.5 (0.03) | 0.21 |
| Multivariate | 0.7 (0.05) | 0.25 | 0.5 (0.04) | 0.21 | 0.5 (0.03) | 0.24 | 0.4 (0.02) | 0.23 | 0.5 (0.03) | 0.22 |
| SFA, g/d |  |  |  |  |  |  |  |  |  |  |
| Unadjusted | 0.4 (0.08) | 0.14 | 0.4 (0.07) | 0.10 | 0.4 (0.03) | 0.19 | 0.3 (0.04) | 0.16 | 0.4 (0.06) | 0.14 |
| Sex and age | 0.4 (0.08) | 0.14 | 0.4 (0.08) | 0.10 | 0.4 (0.04) | 0.19 | 0.3 (0.04) | 0.17 | 0.3 (0.06) | 0.15 |
| Multivariate | 0.4 (0.08) | 0.16 | 0.3 (0.08) | 0.12 | 0.4 (0.04) | 0.20 | 0.3 (0.04) | 0.18 | 0.3 (0.06) | 0.16 |
| MUFA, g/d |  |  |  |  |  |  |  |  |  |  |
| Unadjusted | 0.7 (0.04) | 0.19 | 0.6 (0.04) | 0.22 | 0.5 (0.04) | 0.27 | 0.5 (0.02) | 0.25 | 0.5 (0.04) | 0.25 |
| Sex and age | 0.7 (0.04) | 0.19 | 0.6 (0.04) | 0.22 | 0.6 (0.04) | 0.27 | 0.5 (0.03) | 0.25 | 0.5 (0.04) | 0.25 |
| Multivariate | 0.8 (0.04) | 0.20 | 0.6 (0.04) | 0.22 | 0.5 (0.04) | 0.28 | 0.5 (0.03) | 0.25 | 0.5 (0.04) | 0.26 |
| PUFA, g/d |  |  |  |  |  |  |  |  |  |  |
| Unadjusted | 0.7 (0.03) | 0.28 | 0.5 (0.03) | 0.21 | 0.5 (0.03) | 0.24 | 0.6 (0.02) | 0.24 | 0.5 (0.03) | 0.22 |
| Sex and age | 0.7 (0.03) | 0.28 | 0.5 (0.03) | 0.21 | 0.6 (0.03) | 0.24 | 0.5 (0.02) | 0.24 | 0.5 (0.03) | 0.23 |
| Multivariate | 0.7 (0.03) | 0.29 | 0.5 (0.03) | 0.22 | 0.5 (0.04) | 0.25 | 0.5 (0.02) | 0.25 | 0.5 (0.03) | 0.24 |
| Cholesterol, mg/d |  |  |  |  |  |  |  |  |  |  |
| Unadjusted | 0.8 (0.09) | 0.12 | 0.5 (0.04) | 0.16 | 0.5 (0.04) | 0.17 | 0.5 (0.03) | 0.13 | 0.4 (0.04) | 0.15 |
| Sex and age | 0.8 (0.09) | 0.12 | 0.5 (0.04) | 0.16 | 0.5 (0.04) | 0.17 | 0.4 (0.03) | 0.13 | 0.4 (0.04) | 0.15 |
| Multivariate | 0.8 (0.1) | 0.13 | 0.5 (0.04) | 0.16 | 0.4 (0.04) | 0.18 | 0.4 (0.03) | 0.14 | 0.4 (0.04) | 0.16 |
| Fiber, g/d |  |  |  |  |  |  |  |  |  |  |
| Unadjusted | 0.7 (0.04) | 0.28 | 0.4 (0.02) | 0.15 | 0.4 (0.02) | 0.20 | 0.4 (0.01) | 0.15 | 0.4 (0.02) | 0.15 |
| Sex and age | 0.7 (0.04) | 0.28 | 0.4 (0.02) | 0.15 | 0.4 (0.02) | 0.20 | 0.3 (0.01) | 0.17 | 0.3 (0.02) | 0.18 |
| Multivariate | 0.8 (0.04) | 0.32 | 0.4 (0.02) | 0.19 | 0.4 (0.02) | 0.23 | 0.3 (0.01) | 0.20 | 0.4 (0.02) | 0.21 |
|  |  |  |  |  |  |  |  |  |  |  |
| **Vitamins** |  |  |  |  |  |  |  |  |  |  |
| Vitamin A, μg RAE/d |  |  |  |  |  |  |  |  |  |  |
| Unadjusted | 0.3 (0.05) | 0.06 | 0.4 (0.03) | 0.10 | 0.4 (0.04) | 0.10 | 0.4 (0.03) | 0.10 | 0.4 (0.04) | 0.12 |
| Sex and age | 0.3 (0.05) | 0.06 | 0.4 (0.04) | 0.10 | 0.4 (0.04) | 0.10 | 0.4 (0.03) | 0.10 | 0.4 (0.04) | 0.12 |
| Multivariate | 0.3 (0.05) | 0.07 | 0.4 (0.04) | 0.11 | 0.4 (0.04) | 0.11 | 0.4 (0.03) | 0.10 | 0.4 (0.04) | 0.12 |
| Vitamin D, μg/d |  |  |  |  |  |  |  |  |  |  |
| Unadjusted | 0.5 (0.07) | 0.17 | 0.6 (0.06) | 0.18 | 0.5 (0.04) | 0.21 | 0.6 (0.04) | 0.17 | 0.6 (0.04) | 0.17 |
| Sex and age | 0.5 (0.07) | 0.18 | 0.6 (0.06) | 0.18 | 0.5 (0.04) | 0.21 | 0.5 (0.04) | 0.17 | 0.6 (0.04) | 0.17 |
| Multivariate | 0.6 (0.07) | 0.18 | 0.6 (0.06) | 0.18 | 0.6 (0.04) | 0.21 | 0.5 (0.04) | 0.17 | 0.6 (0.04) | 0.17 |
| Vitamin E, mg/d |  |  |  |  |  |  |  |  |  |  |
| Unadjusted | 0.7 (0.03) | 0.26 | 0.5 (0.03) | 0.23 | 0.5 (0.03) | 0.25 | 0.5 (0.02) | 0.26 | 0.5 (0.02) | 0.24 |
| Sex and age | 0.7 (0.03) | 0.26 | 0.5 (0.03) | 0.23 | 0.5 (0.03) | 0.25 | 0.5 (0.02) | 0.26 | 0.5 (0.03) | 0.24 |
| Multivariate | 0.7 (0.03) | 0.27 | 0.5 (0.03) | 0.24 | 0.5 (0.03) | 0.27 | 0.5 (0.02) | 0.27 | 0.5 (0.03) | 0.26 |
| Thiamine, mg/d |  |  |  |  |  |  |  |  |  |  |
| Unadjusted | 0.7 (0.03) | 0.25 | 0.4 (0.02) | 0.16 | 0.4 (0.02) | 0.19 | 0.4 (0.01) | 0.15 | 0.4 (0.02) | 0.16 |
| Sex and age | 0.7 (0.03) | 0.25 | 0.4 (0.02) | 0.16 | 0.4 (0.02) | 0.19 | 0.3 (0.01) | 0.16 | 0.3 (0.02) | 0.18 |
| Multivariate | 0.7 (0.03) | 0.28 | 0.5 (0.02) | 0.19 | 0.4 (0.02) | 0.21 | 0.3 (0.01) | 0.18 | 0.4 (0.02) | 0.20 |
| Riboflavin, mg/d |  |  |  |  |  |  |  |  |  |  |
| Unadjusted | 0.6 (0.05) | 0.13 | 0.4 (0.02) | 0.18 | 0.4 (0.02) | 0.20 | 0.4 (0.01) | 0.20 | 0.4 (0.02) | 0.20 |
| Sex and age | 0.6 (0.05) | 0.13 | 0.5 (0.02) | 0.18 | 0.4 (0.02) | 0.20 | 0.3 (0.01) | 0.21 | 0.3 (0.02) | 0.22 |
| Multivariate | 0.6 (0.05) | 0.15 | 0.5 (0.02) | 0.19 | 0.4 (0.02) | 0.22 | 0.3 (0.01) | 0.22 | 0.4 (0.02) | 0.24 |
| Niacin, mg/d |  |  |  |  |  |  |  |  |  |  |
| Unadjusted | 0.8 (0.03) | 0.37 | 0.5 (0.02) | 0.30 | 0.5 (0.02) | 0.34 | 0.5 (0.01) | 0.30 | 0.5 (0.02) | 0.28 |
| Sex and age | 0.9 (0.03) | 0.37 | 0.6 (0.02) | 0.30 | 0.5 (0.02) | 0.34 | 0.5 (0.01) | 0.31 | 0.5 (0.02) | 0.29 |
| Multivariate | 0.9 (0.03) | 0.40 | 0.6 (0.02) | 0.33 | 0.5 (0.02) | 0.37 | 0.5 (0.01) | 0.33 | 0.5 (0.02) | 0.33 |
| Vitamin B6, mg/d |  |  |  |  |  |  |  |  |  |  |
| Unadjusted | 0.7 (0.03) | 0.36 | 0.6 (0.02) | 0.38 | 0.6 (0.02) | 0.42 | 0.6 (0.02) | 0.41 | 0.5 (0.02) | 0.32 |
| Sex and age | 0.7 (0.03) | 0.37 | 0.6 (0.02) | 0.38 | 0.6 (0.02) | 0.42 | 0.6 (0.02) | 0.41 | 0.5 (0.02) | 0.33 |
| Multivariate | 0.7 (0.03) | 0.40 | 0.6 (0.02) | 0.42 | 0.6 (0.02) | 0.47 | 0.6 (0.02) | 0.45 | 0.6 (0.02) | 0.38 |
| Folate, μg/d |  |  |  |  |  |  |  |  |  |  |
| Unadjusted | 0.5 (0.04) | 0.16 | 0.4 (0.03) | 0.09 | 0.3 (0.02) | 0.13 | 0.3 (0.02) | 0.11 | 0.3 (0.02) | 0.10 |
| Sex and age | 0.6 (0.04) | 0.16 | 0.4 (0.03) | 0.09 | 0.3 (0.02) | 0.13 | 0.3 (0.02) | 0.11 | 0.3 (0.02) | 0.10 |
| Multivariate | 0.6 (0.04) | 0.18 | 0.4 (0.03) | 0.11 | 0.3 (0.02) | 0.14 | 0.3 (0.02) | 0.13 | 0.3 (0.02) | 0.13 |
| Vitamin C, mg/d |  |  |  |  |  |  |  |  |  |  |
| Unadjusted | 0.4 (0.06) | 0.09 | 0.2 (0.02) | 0.04 | 0.3 (0.03) | 0.09 | 0.2 (0.02) | 0.07 | 0.3 (0.02) | 0.08 |
| Sex and age | 0.4 (0.06) | 0.09 | 0.3 (0.02) | 0.04 | 0.3 (0.03) | 0.09 | 0.2 (0.02) | 0.07 | 0.2 (0.02) | 0.08 |
| Multivariate | 0.4 (0.06) | 0.10 | 0.3 (0.02) | 0.05 | 0.3 (0.03) | 0.10 | 0.2 (0.02) | 0.08 | 0.3 (0.02) | 0.09 |
|  |  |  |  |  |  |  |  |  |  |  |
| **Minerals** |  |  |  |  |  |  |  |  |  |  |
| Calcium, mg/d |  |  |  |  |  |  |  |  |  |  |
| Unadjusted | 0.6 (0.06) | 0.10 | 0.5 (0.04) | 0.15 | 0.4 (0.03) | 0.21 | 0.4 (0.03) | 0.16 | 0.5 (0.03) | 0.20 |
| Sex and age | 0.6 (0.06) | 0.10 | 0.5 (0.04) | 0.15 | 0.5 (0.03) | 0.21 | 0.4 (0.03) | 0.16 | 0.4 (0.03) | 0.20 |
| Multivariate | 0.6 (0.06) | 0.12 | 0.5 (0.03) | 0.16 | 0.5 (0.03) | 0.23 | 0.4 (0.03) | 0.17 | 0.5 (0.04) | 0.22 |
| Iron, mg/d |  |  |  |  |  |  |  |  |  |  |
| Unadjusted | 0.7 (0.04) | 0.25 | 0.4 (0.02) | 0.21 | 0.4 (0.02) | 0.23 | 0.4 (0.02) | 0.18 | 0.4 (0.02) | 0.17 |
| Sex and age | 0.7 (0.04) | 0.25 | 0.4 (0.02) | 0.21 | 0.4 (0.02) | 0.23 | 0.3 (0.02) | 0.20 | 0.4 (0.02) | 0.18 |
| Multivariate | 0.7 (0.04) | 0.27 | 0.5 (0.02) | 0.23 | 0.4 (0.02) | 0.25 | 0.4 (0.02) | 0.21 | 0.4 (0.02) | 0.20 |
| Sodium, mg/d |  |  |  |  |  |  |  |  |  |  |
| Unadjusted | 0.4 (0.03) | 0.06 | 0.2 (0.02) | 0.02 | 0.2 (0.02) | 0.04 | 0.2 (0.02) | 0.01 | 0.2 (0.02) | 0.04 |
| Sex and age | 0.4 (0.03) | 0.06 | 0.2 (0.02) | 0.02 | 0.2 (0.02) | 0.04 | 0.1 (0.02) | 0.02 | 0.2 (0.02) | 0.04 |
| Multivariate | 0.4 (0.03) | 0.07 | 0.2 (0.02) | 0.03 | 0.2 (0.02) | 0.05 | 0.1 (0.02) | 0.03 | 0.2 (0.02) | 0.05 |
| Potassium, mg/d |  |  |  |  |  |  |  |  |  |  |
| Unadjusted | 0.5 (0.04) | 0.21 | 0.4 (0.02) | 0.14 | 0.3 (0.02) | 0.19 | 0.3 (0.01) | 0.16 | 0.3 (0.02) | 0.15 |
| Sex and age | 0.6 (0.04) | 0.21 | 0.4 (0.02) | 0.14 | 0.3 (0.02) | 0.19 | 0.3 (0.01) | 0.17 | 0.3 (0.02) | 0.17 |
| Multivariate | 0.6 (0.04) | 0.23 | 0.4 (0.02) | 0.16 | 0.3 (0.02) | 0.21 | 0.3 (0.01) | 0.19 | 0.3 (0.02) | 0.20 |
| Magnesium, mg/d |  |  |  |  |  |  |  |  |  |  |
| Unadjusted | 0.7 (0.03) | 0.30 | 0.4 (0.02) | 0.17 | 0.4 (0.02) | 0.22 | 0.4 (0.01) | 0.17 | 0.4 (0.02) | 0.17 |
| Sex and age | 0.8 (0.03) | 0.30 | 0.4 (0.02) | 0.17 | 0.4 (0.02) | 0.22 | 0.3 (0.01) | 0.19 | 0.3 (0.02) | 0.19 |
| Multivariate | 0.8 (0.03) | 0.34 | 0.4 (0.02) | 0.22 | 0.4 (0.02) | 0.26 | 0.3 (0.01) | 0.22 | 0.4 (0.02) | 0.23 |
| Zinc, mg/d |  |  |  |  |  |  |  |  |  |  |
| Unadjusted | 0.8 (0.03) | 0.31 | 0.5 (0.02) | 0.19 | 0.4 (0.02) | 0.23 | 0.4 (0.01) | 0.18 | 0.4 (0.02) | 0.19 |
| Sex and age | 0.8 (0.03) | 0.31 | 0.5 (0.02) | 0.19 | 0.4 (0.02) | 0.23 | 0.3 (0.01) | 0.20 | 0.4 (0.02) | 0.21 |
| Multivariate | 0.8 (0.03) | 0.34 | 0.5 (0.02) | 0.23 | 0.4 (0.02) | 0.26 | 0.4 (0.01) | 0.23 | 0.4 (0.02) | 0.26 |

All p-values are <0.001.

On the basis of linear regression models with 24-hour diet recall (24hR) intakes as the dependent variable and individualized Adult Male Equivalent (AME) or per capita (PC) consumption estimates from household questionnaire as the independent variable. The sex and age model was categorized as follows: age was not considered for this analysis; sex, men and women. The multivariate model was adjusted for the following covariates: sex (men, women), education (<6 years of education, ≥6 years of education), religion (Muslims, other), household income (quintiles), respondent’s age (continuous), sex (men, women) and education (<6 years of education, ≥6 years of education), household size, number of children within household, and food wastage percentage (using 24hR data, we calculated for each household, the percent of food wastage -sum of food waste, and food given to guests, others and animals- to total consumed food (mean: 11.6%, SD: 13.6)). *Βs* represent the change in the individual intake (24hR) for every unit increase in the AME mean. SEs for *βs* are presented. R^2^ represents the coefficient of determination for the overall model.

BIHS, Bangladesh Integrated Household Survey; MUFA, Monounsaturated fats; PUFA, Polyunsaturated fats; SFA, Saturated fat

# Table V. Relation between individualized household intake estimates as predictors of individual dietary intakes by education in the 2011-2012 BIHS.

|  | **Low educational level** | | **Medium/High educational level** | |
| --- | --- | --- | --- | --- |
| **Dietary Factor, unit** | ***β,* AME (SE)** | **R** | ***β,* AME (SE)** | **R** |
| **Food groups** |  |  |  |  |
| Fruits, g/d |  |  |  |  |
| Unadjusted | 0.1 (0.02) | 0.06 | 0.1 (0.02) | 0.06 |
| Sex and age | 0.1 (0.02) | 0.06 | 0.1 (0.02) | 0.06 |
| Multivariate | 0.1 (0.02) | 0.06 | 0.2 (0.02) | 0.07 |
| Non-starchy vegetables, g/d |  |  |  |  |
| Unadjusted | 0.5 (0.01) | 0.15 | 0.3 (0.02) | 0.11 |
| Sex and age | 0.3 (0.01) | 0.19 | 0.3 (0.02) | 0.12 |
| Multivariate | 0.3 (0.02) | 0.20 | 0.3 (0.02) | 0.13 |
| Starchy vegetables, g/d |  |  |  |  |
| Unadjusted | 0.8 (0.02) | 0.17 | 0.4 (0.04) | 0.09 |
| Sex and age | 0.5 (0.03) | 0.20 | 0.4 (0.04) | 0.09 |
| Multivariate | 0.6 (0.03) | 0.21 | 0.4 (0.04) | 0.11 |
| Legumes, g/d |  |  |  |  |
| Unadjusted | 0.6 (0.04) | 0.14 | 0.5 (0.05) | 0.10 |
| Sex and age | 0.6 (0.04) | 0.14 | 0.5 (0.05) | 0.10 |
| Multivariate | 0.6 (0.04) | 0.14 | 0.5 (0.06) | 0.11 |
| Total grains, g/d |  |  |  |  |
| Unadjusted | 0.9 (0.01) | 0.53 | 0.4 (0.02) | 0.22 |
| Sex and age | 0.5 (0.01) | 0.61 | 0.4 (0.02) | 0.24 |
| Multivariate | 0.6 (0.01) | 0.64 | 0.5 (0.02) | 0.30 |
| Meat/Eggs, g/d |  |  |  |  |
| Unadjusted | 0.4 (0.04) | 0.12 | 0.4 (0.04) | 0.12 |
| Sex and age | 0.4 (0.04) | 0.12 | 0.4 (0.04) | 0.12 |
| Multivariate | 0.4 (0.04) | 0.13 | 0.4 (0.04) | 0.15 |
| Seafood, g/d |  |  |  |  |
| Unadjusted | 0.7 (0.02) | 0.22 | 0.5 (0.03) | 0.19 |
| Sex and age | 0.5 (0.02) | 0.23 | 0.5 (0.03) | 0.19 |
| Multivariate | 0.5 (0.02) | 0.24 | 0.5 (0.03) | 0.20 |
| Milk, whole fat, g/d |  |  |  |  |
| Unadjusted | 0.5 (0.03) | 0.21 | 0.5 (0.03) | 0.33 |
| Sex and age | 0.5 (0.03) | 0.23 | 0.5 (0.03) | 0.33 |
| Multivariate | 0.5 (0.03) | 0.24 | 0.5 (0.03) | 0.33 |
| Fats/Oils, g/d |  |  |  |  |
| Unadjusted | 0.7 (0.02) | 0.21 | 0.5 (0.03) | 0.16 |
| Sex and age | 0.5 (0.02) | 0.23 | 0.5 (0.03) | 0.16 |
| Multivariate | 0.5 (0.02) | 0.23 | 0.4 (0.03) | 0.17 |
|  |  |  |  |  |
| **Energy & Macronutrients** |  |  |  |  |
| Energy, kcal/d |  |  |  |  |
| Unadjusted | 0.8 (0.01) | 0.50 | 0.4 (0.01) | 0.18 |
| Sex and age | 0.5 (0.01) | 0.59 | 0.3 (0.02) | 0.21 |
| Multivariate | 0.5 (0.01) | 0.60 | 0.4 (0.02) | 0.24 |
| Protein, g/d |  |  |  |  |
| Unadjusted | 0.8 (0.01) | 0.43 | 0.4 (0.02) | 0.17 |
| Sex and age | 0.5 (0.01) | 0.50 | 0.3 (0.02) | 0.19 |
| Multivariate | 0.5 (0.01) | 0.52 | 0.4 (0.02) | 0.22 |
| Carbohydrates, g/d |  |  |  |  |
| Unadjusted | 0.8 (0.01) | 0.51 | 0.4 (0.02) | 0.17 |
| Sex and age | 0.5 (0.01) | 0.60 | 0.3 (0.02) | 0.20 |
| Multivariate | 0.5 (0.01) | 0.62 | 0.4 (0.02) | 0.25 |
| Total fat, g/d |  |  |  |  |
| Unadjusted | 0.6 (0.01) | 0.26 | 0.5 (0.02) | 0.20 |
| Sex and age | 0.5 (0.02) | 0.27 | 0.4 (0.02) | 0.20 |
| Multivariate | 0.5 (0.02) | 0.28 | 0.4 (0.03) | 0.21 |
| SFA, g/d |  |  |  |  |
| Unadjusted | 0.4 (0.03) | 0.18 | 0.3 (0.04) | 0.14 |
| Sex and age | 0.3 (0.04) | 0.19 | 0.3 (0.04) | 0.14 |
| Multivariate | 0.3 (0.04) | 0.19 | 0.3 (0.04) | 0.15 |
| MUFA, g/d |  |  |  |  |
| Unadjusted | 0.6 (0.02) | 0.28 | 0.5 (0.03) | 0.26 |
| Sex and age | 0.5 (0.02) | 0.29 | 0.5 (0.03) | 0.26 |
| Multivariate | 0.5 (0.02) | 0.29 | 0.5 (0.04) | 0.27 |
| PUFA, g/d |  |  |  |  |
| Unadjusted | 0.7 (0.02) | 0.28 | 0.5 (0.02) | 0.20 |
| Sex and age | 0.6 (0.02) | 0.30 | 0.5 (0.02) | 0.21 |
| Multivariate | 0.6 (0.02) | 0.30 | 0.5 (0.03) | 0.22 |
| Cholesterol, mg/d |  |  |  |  |
| Unadjusted | 0.4 (0.02) | 0.12 | 0.5 (0.04) | 0.14 |
| Sex and age | 0.4 (0.03) | 0.12 | 0.5 (0.04) | 0.14 |
| Multivariate | 0.3 (0.03) | 0.12 | 0.4 (0.04) | 0.15 |
| Fiber, g/d |  |  |  |  |
| Unadjusted | 0.8 (0.01) | 0.42 | 0.3 (0.02) | 0.14 |
| Sex and age | 0.4 (0.01) | 0.52 | 0.3 (0.02) | 0.16 |
| Multivariate | 0.4 (0.01) | 0.54 | 0.3 (0.02) | 0.21 |
|  |  |  |  |  |
| **Vitamins** |  |  |  |  |
| Vitamin A, μg RAE/d |  |  |  |  |
| Unadjusted | 0.5 (0.03) | 0.11 | 0.3 (0.03) | 0.11 |
| Sex and age | 0.4 (0.03) | 0.12 | 0.3 (0.03) | 0.11 |
| Multivariate | 0.4 (0.03) | 0.12 | 0.3 (0.03) | 0.12 |
| Vitamin D, μg/d |  |  |  |  |
| Unadjusted | 0.6 (0.03) | 0.16 | 0.6 (0.05) | 0.21 |
| Sex and age | 0.5 (0.04) | 0.17 | 0.6 (0.05) | 0.21 |
| Multivariate | 0.5 (0.04) | 0.17 | 0.6 (0.05) | 0.21 |
| Vitamin E, mg/d |  |  |  |  |
| Unadjusted | 0.7 (0.01) | 0.31 | 0.5 (0.02) | 0.23 |
| Sex and age | 0.5 (0.02) | 0.33 | 0.5 (0.02) | 0.23 |
| Multivariate | 0.5 (0.02) | 0.33 | 0.5 (0.02) | 0.24 |
| Thiamine, mg/d |  |  |  |  |
| Unadjusted | 0.7 (0.01) | 0.38 | 0.3 (0.02) | 0.14 |
| Sex and age | 0.4 (0.01) | 0.46 | 0.3 (0.02) | 0.15 |
| Multivariate | 0.4 (0.01) | 0.46 | 0.3 (0.02) | 0.17 |
| Riboflavin, mg/d |  |  |  |  |
| Unadjusted | 0.6 (0.01) | 0.29 | 0.4 (0.02) | 0.20 |
| Sex and age | 0.4 (0.01) | 0.32 | 0.3 (0.02) | 0.22 |
| Multivariate | 0.4 (0.01) | 0.33 | 0.3 (0.02) | 0.24 |
| Niacin, mg/d |  |  |  |  |
| Unadjusted | 0.8 (0.01) | 0.54 | 0.5 (0.02) | 0.28 |
| Sex and age | 0.6 (0.01) | 0.60 | 0.4 (0.02) | 0.29 |
| Multivariate | 0.6 (0.01) | 0.61 | 0.5 (0.02) | 0.33 |
| Vitamin B6, mg/d |  |  |  |  |
| Unadjusted | 0.8 (0.01) | 0.52 | 0.5 (0.02) | 0.35 |
| Sex and age | 0.6 (0.02) | 0.55 | 0.5 (0.02) | 0.35 |
| Multivariate | 0.6 (0.01) | 0.58 | 0.5 (0.02) | 0.41 |
| Folate, μg/d |  |  |  |  |
| Unadjusted | 0.6 (0.01) | 0.21 | 0.3 (0.02) | 0.10 |
| Sex and age | 0.4 (0.02) | 0.26 | 0.3 (0.02) | 0.10 |
| Multivariate | 0.4 (0.02) | 0.27 | 0.3 (0.02) | 0.13 |
| Vitamin C, mg/d |  |  |  |  |
| Unadjusted | 0.5 (0.02) | 0.11 | 0.2 (0.02) | 0.08 |
| Sex and age | 0.3 (0.02) | 0.15 | 0.2 (0.02) | 0.08 |
| Multivariate | 0.3 (0.02) | 0.15 | 0.2 (0.02) | 0.09 |
|  |  |  |  |  |
| **Minerals** |  |  |  |  |
| Calcium, mg/d |  |  |  |  |
| Unadjusted | 0.6 (0.02) | 0.21 | 0.4 (0.03) | 0.20 |
| Sex and age | 0.4 (0.02) | 0.21 | 0.4 (0.03) | 0.20 |
| Multivariate | 0.4 (0.02) | 0.22 | 0.4 (0.03) | 0.22 |
| Iron, mg/d |  |  |  |  |
| Unadjusted | 0.7 (0.01) | 0.34 | 0.4 (0.02) | 0.17 |
| Sex and age | 0.4 (0.02) | 0.40 | 0.3 (0.02) | 0.19 |
| Multivariate | 0.5 (0.02) | 0.41 | 0.4 (0.02) | 0.21 |
| Sodium, mg/d |  |  |  |  |
| Unadjusted | 0.5 (0.01) | 0.09 | 0.2 (0.02) | 0.03 |
| Sex and age | 0.2 (0.01) | 0.16 | 0.1 (0.02) | 0.03 |
| Multivariate | 0.2 (0.01) | 0.17 | 0.1 (0.02) | 0.04 |
| Potassium, mg/d |  |  |  |  |
| Unadjusted | 0.7 (0.01) | 0.35 | 0.3 (0.02) | 0.16 |
| Sex and age | 0.4 (0.01) | 0.44 | 0.3 (0.02) | 0.18 |
| Multivariate | 0.4 (0.01) | 0.45 | 0.3 (0.02) | 0.21 |
| Magnesium, mg/d |  |  |  |  |
| Unadjusted | 0.8 (0.01) | 0.46 | 0.3 (0.02) | 0.16 |
| Sex and age | 0.4 (0.01) | 0.55 | 0.3 (0.02) | 0.19 |
| Multivariate | 0.5 (0.01) | 0.57 | 0.3 (0.02) | 0.23 |
| Zinc, mg/d |  |  |  |  |
| Unadjusted | 0.8 (0.01) | 0.47 | 0.4 (0.02) | 0.16 |
| Sex and age | 0.5 (0.01) | 0.56 | 0.3 (0.02) | 0.19 |
| Multivariate | 0.5 (0.01) | 0.58 | 0.3 (0.02) | 0.23 |

On the basis of linear regression models with 24-hour diet recall (24hR) intakes as the dependent variable and individualized Adult Male Equivalent (AME) or per capita (PC) consumption estimates from household questionnaire as the independent variable. The sex and age model was categorized as follows: age, ≤5, 6-10, 11-19, 20-44, and ≥45 years; sex, men and women. The multivariate model was adjusted for the following covariates: age (≤5, 6-10, 11-19, 20-44, and ≥45 years),sex (men, women), religion (Muslims, other), household income (quintiles), respondent’s age (continuous), sex (men, women) and education (<6 years of education, ≥6 years of education), household size, number of children within household, and food wastage percentage (using 24hR data, we calculated for each household, the percent of food wastage -sum of food waste, and food given to guests, others and animals- to total consumed food (mean: 11.6%, SD: 13.6)). *Β*s represents the change in the individual intake (24hR) for every unit increase in the AME mean. SEs for *βs* are presented. R^2^ represents the coefficient of determination for the overall model. The analysis was realized only for the adult population (≥20 years) of the Bangladesh Integrated Household Survey.

BIHS, Bangladesh Integrated Household Survey; MUFA, Monounsaturated fats; PUFA, Polyunsaturated fats; SFA, Saturated fat

# Table W. Relation between individualized household intake estimates as predictors of individual dietary intakes by religion in the 2011-2012 BIHS.

|  | **Muslims** | | **Other religions** | |
| --- | --- | --- | --- | --- |
| **Dietary Factor, unit** | ***β,* AME (SE)** | **R** | ***β,* AME (SE)** | **R** |
| **Food groups** |  |  |  |  |
| Fruits, g/d |  |  |  |  |
| Unadjusted | 0.1 (0.02) | 0.06 | 0.1 (0.04) | 0.04 |
| Sex and age | 0.1 (0.02) | 0.06 | 0.1 (0.04) | 0.04 |
| Multivariate | 0.1 (0.02) | 0.07 | 0.1 (0.04) | 0.08 |
| Non-starchy vegetables, g/d |  |  |  |  |
| Unadjusted | 0.5 (0.01) | 0.15 | 0.5 (0.03) | 0.15 |
| Sex and age | 0.3 (0.02) | 0.19 | 0.3 (0.04) | 0.19 |
| Multivariate | 0.3 (0.02) | 0.20 | 0.3 (0.04) | 0.20 |
| Starchy vegetables, g/d |  |  |  |  |
| Unadjusted | 0.8 (0.02) | 0.15 | 0.9 (0.06) | 0.17 |
| Sex and age | 0.5 (0.03) | 0.17 | 0.6 (0.08) | 0.19 |
| Multivariate | 0.5 (0.03) | 0.18 | 0.6 (0.08) | 0.24 |
| Legumes, g/d |  |  |  |  |
| Unadjusted | 0.6 (0.04) | 0.13 | 0.7 (0.11) | 0.10 |
| Sex and age | 0.5 (0.04) | 0.14 | 0.5 (0.12) | 0.10 |
| Multivariate | 0.5 (0.04) | 0.14 | 0.5 (0.12) | 0.13 |
| Total grains, g/d |  |  |  |  |
| Unadjusted | 0.9 (0.01) | 0.49 | 0.8 (0.03) | 0.46 |
| Sex and age | 0.5 (0.01) | 0.57 | 0.5 (0.04) | 0.55 |
| Multivariate | 0.6 (0.01) | 0.60 | 0.5 (0.04) | 0.59 |
| Meat/Eggs, g/d |  |  |  |  |
| Unadjusted | 0.4 (0.03) | 0.13 | 0.3 (0.08) | 0.10 |
| Sex and age | 0.4 (0.03) | 0.13 | 0.3 (0.09) | 0.10 |
| Multivariate | 0.4 (0.04) | 0.14 | 0.3 (0.09) | 0.18 |
| Seafood, g/d |  |  |  |  |
| Unadjusted | 0.7 (0.02) | 0.21 | 0.6 (0.06) | 0.30 |
| Sex and age | 0.5 (0.02) | 0.22 | 0.5 (0.07) | 0.31 |
| Multivariate | 0.5 (0.02) | 0.23 | 0.5 (0.06) | 0.32 |
| Milk, whole fat, g/d |  |  |  |  |
| Unadjusted | 0.4 (0.03) | 0.22 | 0.5 (0.06) | 0.42 |
| Sex and age | 0.4 (0.03) | 0.24 | 0.6 (0.06) | 0.44 |
| Multivariate | 0.4 (0.03) | 0.24 | 0.6 (0.06) | 0.45 |
| Fats/Oils, g/d |  |  |  |  |
| Unadjusted | 0.7 (0.02) | 0.22 | 0.7 (0.05) | 0.29 |
| Sex and age | 0.5 (0.02) | 0.23 | 0.5 (0.06) | 0.29 |
| Multivariate | 0.5 (0.02) | 0.24 | 0.5 (0.06) | 0.30 |
|  |  |  |  |  |
| **Energy & Macronutrients** |  |  |  |  |
| Energy, kcal/d |  |  |  |  |
| Unadjusted | 0.8 (0.01) | 0.46 | 0.8 (0.02) | 0.47 |
| Sex and age | 0.4 (0.01) | 0.55 | 0.5 (0.03) | 0.55 |
| Multivariate | 0.5 (0.01) | 0.57 | 0.5 (0.03) | 0.57 |
| Protein, g/d |  |  |  |  |
| Unadjusted | 0.8 (0.01) | 0.39 | 0.8 (0.02) | 0.40 |
| Sex and age | 0.4 (0.01) | 0.47 | 0.5 (0.03) | 0.47 |
| Multivariate | 0.5 (0.01) | 0.49 | 0.5 (0.03) | 0.49 |
| Carbohydrates, g/d |  |  |  |  |
| Unadjusted | 0.8 (0.01) | 0.46 | 0.8 (0.02) | 0.47 |
| Sex and age | 0.4 (0.01) | 0.55 | 0.5 (0.03) | 0.55 |
| Multivariate | 0.5 (0.01) | 0.58 | 0.5 (0.03) | 0.58 |
| Total fat, g/d |  |  |  |  |
| Unadjusted | 0.6 (0.01) | 0.27 | 0.7 (0.03) | 0.32 |
| Sex and age | 0.5 (0.02) | 0.28 | 0.5 (0.05) | 0.32 |
| Multivariate | 0.5 (0.02) | 0.29 | 0.5 (0.05) | 0.33 |
| SFA, g/d |  |  |  |  |
| Unadjusted | 0.4 (0.04) | 0.18 | 0.4 (0.06) | 0.18 |
| Sex and age | 0.3 (0.04) | 0.20 | 0.3 (0.05) | 0.19 |
| Multivariate | 0.3 (0.04) | 0.20 | 0.3 (0.05) | 0.20 |
| MUFA, g/d |  |  |  |  |
| Unadjusted | 0.6 (0.01) | 0.28 | 0.7 (0.04) | 0.41 |
| Sex and age | 0.5 (0.02) | 0.29 | 0.6 (0.06) | 0.41 |
| Multivariate | 0.5 (0.02) | 0.29 | 0.6 (0.06) | 0.42 |
| PUFA, g/d |  |  |  |  |
| Unadjusted | 0.7 (0.01) | 0.28 | 0.7 (0.04) | 0.35 |
| Sex and age | 0.6 (0.02) | 0.30 | 0.6 (0.06) | 0.35 |
| Multivariate | 0.6 (0.02) | 0.30 | 0.6 (0.06) | 0.37 |
| Cholesterol, mg/d |  |  |  |  |
| Unadjusted | 0.4 (0.03) | 0.14 | 0.5 (0.09) | 0.19 |
| Sex and age | 0.3 (0.03) | 0.14 | 0.5 (0.1) | 0.20 |
| Multivariate | 0.3 (0.03) | 0.14 | 0.5 (0.09) | 0.26 |
| Fiber, g/d |  |  |  |  |
| Unadjusted | 0.7 (0.01) | 0.38 | 0.7 (0.02) | 0.37 |
| Sex and age | 0.4 (0.01) | 0.48 | 0.4 (0.03) | 0.47 |
| Multivariate | 0.4 (0.01) | 0.50 | 0.4 (0.03) | 0.49 |
|  |  |  |  |  |
| **Vitamins** |  |  |  |  |
| Vitamin A, μg RAE/d |  |  |  |  |
| Unadjusted | 0.4 (0.03) | 0.11 | 0.5 (0.08) | 0.16 |
| Sex and age | 0.4 (0.03) | 0.11 | 0.5 (0.09) | 0.16 |
| Multivariate | 0.4 (0.03) | 0.11 | 0.5 (0.09) | 0.17 |
| Vitamin D, μg/d |  |  |  |  |
| Unadjusted | 0.6 (0.04) | 0.19 | 0.5 (0.07) | 0.14 |
| Sex and age | 0.6 (0.04) | 0.19 | 0.4 (0.08) | 0.14 |
| Multivariate | 0.6 (0.04) | 0.19 | 0.4 (0.08) | 0.15 |
| Vitamin E, mg/d |  |  |  |  |
| Unadjusted | 0.7 (0.01) | 0.32 | 0.7 (0.03) | 0.37 |
| Sex and age | 0.5 (0.02) | 0.33 | 0.6 (0.05) | 0.37 |
| Multivariate | 0.5 (0.02) | 0.34 | 0.5 (0.05) | 0.39 |
| Thiamine, mg/d |  |  |  |  |
| Unadjusted | 0.7 (0.01) | 0.34 | 0.7 (0.03) | 0.34 |
| Sex and age | 0.4 (0.01) | 0.42 | 0.4 (0.04) | 0.40 |
| Multivariate | 0.4 (0.01) | 0.43 | 0.5 (0.04) | 0.42 |
| Riboflavin, mg/d |  |  |  |  |
| Unadjusted | 0.5 (0.01) | 0.28 | 0.6 (0.03) | 0.37 |
| Sex and age | 0.3 (0.01) | 0.32 | 0.4 (0.04) | 0.39 |
| Multivariate | 0.3 (0.01) | 0.33 | 0.4 (0.04) | 0.42 |
| Niacin, mg/d |  |  |  |  |
| Unadjusted | 0.8 (0.01) | 0.50 | 0.8 (0.02) | 0.52 |
| Sex and age | 0.5 (0.01) | 0.56 | 0.6 (0.03) | 0.57 |
| Multivariate | 0.6 (0.01) | 0.58 | 0.6 (0.03) | 0.60 |
| Vitamin B6, mg/d |  |  |  |  |
| Unadjusted | 0.8 (0.01) | 0.49 | 0.8 (0.03) | 0.51 |
| Sex and age | 0.6 (0.02) | 0.51 | 0.6 (0.03) | 0.54 |
| Multivariate | 0.6 (0.01) | 0.55 | 0.6 (0.03) | 0.57 |
| Folate, μg/d |  |  |  |  |
| Unadjusted | 0.6 (0.01) | 0.19 | 0.6 (0.03) | 0.21 |
| Sex and age | 0.3 (0.02) | 0.24 | 0.3 (0.04) | 0.25 |
| Multivariate | 0.4 (0.02) | 0.25 | 0.4 (0.04) | 0.28 |
| Vitamin C, mg/d |  |  |  |  |
| Unadjusted | 0.4 (0.02) | 0.11 | 0.4 (0.04) | 0.12 |
| Sex and age | 0.2 (0.02) | 0.13 | 0.3 (0.04) | 0.15 |
| Multivariate | 0.3 (0.02) | 0.14 | 0.3 (0.05) | 0.17 |
|  |  |  |  |  |
| **Minerals** |  |  |  |  |
| Calcium, mg/d |  |  |  |  |
| Unadjusted | 0.5 (0.02) | 0.21 | 0.6 (0.04) | 0.27 |
| Sex and age | 0.4 (0.02) | 0.21 | 0.4 (0.05) | 0.27 |
| Multivariate | 0.4 (0.02) | 0.22 | 0.4 (0.05) | 0.28 |
| Iron, mg/d |  |  |  |  |
| Unadjusted | 0.7 (0.01) | 0.31 | 0.7 (0.03) | 0.40 |
| Sex and age | 0.4 (0.01) | 0.37 | 0.5 (0.04) | 0.44 |
| Multivariate | 0.4 (0.01) | 0.38 | 0.5 (0.04) | 0.46 |
| Sodium, mg/d |  |  |  |  |
| Unadjusted | 0.5 (0.01) | 0.07 | 0.6 (0.03) | 0.10 |
| Sex and age | 0.2 (0.01) | 0.13 | 0.3 (0.04) | 0.16 |
| Multivariate | 0.2 (0.01) | 0.14 | 0.3 (0.04) | 0.17 |
| Potassium, mg/d |  |  |  |  |
| Unadjusted | 0.6 (0.01) | 0.32 | 0.6 (0.02) | 0.36 |
| Sex and age | 0.3 (0.01) | 0.41 | 0.4 (0.03) | 0.44 |
| Multivariate | 0.4 (0.01) | 0.43 | 0.4 (0.03) | 0.46 |
| Magnesium, mg/d |  |  |  |  |
| Unadjusted | 0.7 (0.01) | 0.41 | 0.7 (0.02) | 0.42 |
| Sex and age | 0.4 (0.01) | 0.51 | 0.4 (0.03) | 0.51 |
| Multivariate | 0.4 (0.01) | 0.54 | 0.5 (0.03) | 0.53 |
| Zinc, mg/d |  |  |  |  |
| Unadjusted | 0.8 (0.01) | 0.43 | 0.8 (0.02) | 0.43 |
| Sex and age | 0.4 (0.01) | 0.52 | 0.4 (0.03) | 0.52 |
| Multivariate | 0.4 (0.01) | 0.54 | 0.5 (0.03) | 0.54 |

All p-values are <0.001.

On the basis of linear regression models with 24-hour diet recall (24hR) intakes as the dependent variable and individualized Adult Male Equivalent (AME) or per capita (PC) consumption estimates from household questionnaire as the independent variable. The sex and age model was categorized as follows: age, ≤5, 6-10, 11-19, 20-44, and ≥45 years; sex, men and women. The multivariate model was adjusted for the following covariates: age (≤5, 6-10, 11-19, 20-44, and ≥45 years), sex (men, women), education (<6 years of education, ≥6 years of education), household income (quintiles), respondent’s age (continuous), sex (men, women) and education (<6 years of education, ≥6 years of education), household size, number of children within household, and food wastage percentage (using 24hR data, we calculated for each household, the percent of food wastage -sum of food waste, and food given to guests, others and animals- to total consumed food (mean: 11.6%, SD: 13.6)). *Βs* represent the change in the individual intake (24hR) for every unit increase in the AME mean. SEs for *β*s are presented. R^2^ represents the coefficient of determination for the overall model.

BIHS, Bangladesh Integrated Household Survey; MUFA, Monounsaturated fats; PUFA, Polyunsaturated fats; SFA, Saturated fat

# Table X. Relation between individualized household intake estimates as predictors of individual dietary intakes by household income in the 2011-2012 BIHS.

|  | **1^st^ quintile** | | **2^nd^ quintile** | | **3^rd^ quintile** | | **4^th^ quintile** | | **5^th^ quintile** | |
| --- | --- | --- | --- | --- | --- | --- | --- | --- | --- | --- |
| **Dietary Factor, unit** | ***β,* AME (SE)** | **R** | ***β,* AME (SE)** | **R** | ***β,* AME (SE)** | **R** | ***β,* AME (SE)** | **R** | ***β,* AME (SE)** | **R** |
| **Food groups** |  |  |  |  |  |  |  |  |  |  |
| Fruits, g/d |  |  |  |  |  |  |  |  |  |  |
| Unadjusted | 0.2 (0.04) | 0.07 | 0.1 (0.03) | 0.04 | 0.1 (0.04) | 0.06 | 0.1 (0.04) | 0.05 | 0.1 (0.04) | 0.07 |
| Sex and age | 0.2 (0.04) | 0.07 | 0.1 (0.03) | 0.04 | 0.1 (0.04) | 0.07 | 0.1 (0.04) | 0.05 | 0.1 (0.05) | 0.08 |
| Multivariate | 0.2 (0.05) | 0.07 | 0.1 (0.03) | 0.04 | 0.1 (0.04) | 0.08 | 0.1 (0.04) | 0.07 | 0.1 (0.05) | 0.07 |
| Non-starchy vegetables, g/d |  |  |  |  |  |  |  |  |  |  |
| Unadjusted | 0.5 (0.03) | 0.14 | 0.6 (0.03) | 0.19 | 0.5 (0.02) | 0.17 | 0.5 (0.03) | 0.14 | 0.5 (0.03) | 0.13 |
| Sex and age | 0.3 (0.03) | 0.18 | 0.4 (0.03) | 0.23 | 0.3 (0.03) | 0.20 | 0.3 (0.03) | 0.19 | 0.3 (0.03) | 0.15 |
| Multivariate | 0.3 (0.03) | 0.21 | 0.4 (0.04) | 0.24 | 0.3 (0.03) | 0.21 | 0.3 (0.03) | 0.20 | 0.3 (0.03) | 0.16 |
| Starchy vegetables, g/d |  |  |  |  |  |  |  |  |  |  |
| Unadjusted | 0.7 (0.04) | 0.12 | 0.8 (0.04) | 0.16 | 0.9 (0.05) | 0.16 | 0.9 (0.04) | 0.16 | 0.9 (0.05) | 0.17 |
| Sex and age | 0.4 (0.04) | 0.15 | 0.5 (0.04) | 0.19 | 0.6 (0.08) | 0.19 | 0.6 (0.06) | 0.18 | 0.6 (0.06) | 0.18 |
| Multivariate | 0.4 (0.04) | 0.17 | 0.6 (0.04) | 0.20 | 0.6 (0.08) | 0.19 | 0.6 (0.06) | 0.19 | 0.6 (0.06) | 0.20 |
| Legumes, g/d |  |  |  |  |  |  |  |  |  |  |
| Unadjusted | 0.6 (0.07) | 0.14 | 0.5 (0.08) | 0.10 | 0.7 (0.1) | 0.21 | 0.7 (0.11) | 0.13 | 0.5 (0.07) | 0.09 |
| Sex and age | 0.5 (0.07) | 0.14 | 0.5 (0.08) | 0.11 | 0.7 (0.11) | 0.21 | 0.7 (0.13) | 0.13 | 0.4 (0.07) | 0.09 |
| Multivariate | 0.5 (0.07) | 0.15 | 0.4 (0.08) | 0.12 | 0.7 (0.11) | 0.22 | 0.7 (0.13) | 0.14 | 0.4 (0.07) | 0.10 |
| Total grains, g/d |  |  |  |  |  |  |  |  |  |  |
| Unadjusted | 0.8 (0.02) | 0.45 | 0.9 (0.02) | 0.49 | 0.9 (0.02) | 0.51 | 0.9 (0.02) | 0.50 | 0.9 (0.03) | 0.45 |
| Sex and age | 0.5 (0.03) | 0.55 | 0.5 (0.03) | 0.57 | 0.5 (0.03) | 0.60 | 0.5 (0.03) | 0.59 | 0.5 (0.04) | 0.54 |
| Multivariate | 0.5 (0.03) | 0.59 | 0.5 (0.03) | 0.61 | 0.6 (0.03) | 0.62 | 0.6 (0.03) | 0.61 | 0.5 (0.03) | 0.58 |
| Meat/Eggs, g/d |  |  |  |  |  |  |  |  |  |  |
| Unadjusted | 0.4 (0.07) | 0.19 | 0.4 (0.06) | 0.15 | 0.5 (0.07) | 0.12 | 0.4 (0.06) | 0.13 | 0.4 (0.06) | 0.07 |
| Sex and age | 0.4 (0.07) | 0.19 | 0.4 (0.06) | 0.15 | 0.4 (0.08) | 0.12 | 0.4 (0.07) | 0.13 | 0.3 (0.06) | 0.07 |
| Multivariate | 0.4 (0.08) | 0.21 | 0.4 (0.07) | 0.15 | 0.4 (0.08) | 0.13 | 0.4 (0.07) | 0.16 | 0.3 (0.06) | 0.11 |
| Seafood, g/d |  |  |  |  |  |  |  |  |  |  |
| Unadjusted | 0.6 (0.04) | 0.18 | 0.8 (0.05) | 0.21 | 0.6 (0.05) | 0.23 | 0.7 (0.05) | 0.22 | 0.7 (0.04) | 0.23 |
| Sex and age | 0.5 (0.05) | 0.19 | 0.6 (0.05) | 0.22 | 0.5 (0.05) | 0.24 | 0.6 (0.05) | 0.23 | 0.5 (0.05) | 0.24 |
| Multivariate | 0.5 (0.05) | 0.22 | 0.6 (0.05) | 0.22 | 0.5 (0.05) | 0.24 | 0.6 (0.05) | 0.24 | 0.5 (0.05) | 0.24 |
| Milk, whole fat, g/d |  |  |  |  |  |  |  |  |  |  |
| Unadjusted | 0.4 (0.05) | 0.24 | 0.4 (0.08) | 0.25 | 0.4 (0.06) | 0.24 | 0.4 (0.05) | 0.17 | 0.4 (0.05) | 0.30 |
| Sex and age | 0.5 (0.04) | 0.26 | 0.4 (0.08) | 0.25 | 0.4 (0.06) | 0.26 | 0.4 (0.04) | 0.18 | 0.5 (0.05) | 0.33 |
| Multivariate | 0.5 (0.05) | 0.27 | 0.4 (0.08) | 0.26 | 0.4 (0.06) | 0.26 | 0.4 (0.05) | 0.18 | 0.5 (0.05) | 0.33 |
| Fats/Oils, g/d |  |  |  |  |  |  |  |  |  |  |
| Unadjusted | 0.7 (0.03) | 0.22 | 0.7 (0.03) | 0.24 | 0.8 (0.03) | 0.22 | 0.7 (0.04) | 0.22 | 0.7 (0.03) | 0.20 |
| Sex and age | 0.5 (0.04) | 0.23 | 0.6 (0.05) | 0.26 | 0.6 (0.05) | 0.24 | 0.5 (0.06) | 0.24 | 0.5 (0.04) | 0.22 |
| Multivariate | 0.5 (0.04) | 0.24 | 0.5 (0.05) | 0.26 | 0.6 (0.05) | 0.24 | 0.5 (0.06) | 0.25 | 0.5 (0.04) | 0.23 |
|  |  |  |  |  |  |  |  |  |  |  |
| **Energy & Macronutrients** |  |  |  |  |  |  |  |  |  |  |
| Energy, kcal/d |  |  |  |  |  |  |  |  |  |  |
| Unadjusted | 0.7 (0.02) | 0.41 | 0.8 (0.01) | 0.50 | 0.8 (0.02) | 0.51 | 0.8 (0.02) | 0.45 | 0.8 (0.02) | 0.43 |
| Sex and age | 0.4 (0.02) | 0.52 | 0.5 (0.02) | 0.58 | 0.5 (0.02) | 0.59 | 0.4 (0.02) | 0.54 | 0.4 (0.03) | 0.51 |
| Multivariate | 0.4 (0.02) | 0.55 | 0.5 (0.02) | 0.60 | 0.5 (0.02) | 0.60 | 0.5 (0.02) | 0.56 | 0.5 (0.02) | 0.53 |
| Protein, g/d |  |  |  |  |  |  |  |  |  |  |
| Unadjusted | 0.7 (0.02) | 0.37 | 0.8 (0.02) | 0.42 | 0.8 (0.02) | 0.43 | 0.8 (0.02) | 0.39 | 0.7 (0.02) | 0.36 |
| Sex and age | 0.4 (0.02) | 0.46 | 0.5 (0.03) | 0.48 | 0.5 (0.03) | 0.49 | 0.4 (0.03) | 0.47 | 0.4 (0.03) | 0.44 |
| Multivariate | 0.4 (0.02) | 0.48 | 0.5 (0.03) | 0.50 | 0.5 (0.03) | 0.50 | 0.5 (0.02) | 0.49 | 0.4 (0.03) | 0.45 |
| Carbohydrates, g/d |  |  |  |  |  |  |  |  |  |  |
| Unadjusted | 0.7 (0.02) | 0.41 | 0.8 (0.02) | 0.49 | 0.8 (0.02) | 0.51 | 0.8 (0.02) | 0.45 | 0.8 (0.02) | 0.44 |
| Sex and age | 0.4 (0.02) | 0.53 | 0.5 (0.02) | 0.57 | 0.5 (0.02) | 0.59 | 0.4 (0.02) | 0.55 | 0.5 (0.02) | 0.53 |
| Multivariate | 0.4 (0.02) | 0.56 | 0.5 (0.02) | 0.60 | 0.5 (0.02) | 0.61 | 0.5 (0.02) | 0.57 | 0.5 (0.02) | 0.55 |
| Total fat, g/d |  |  |  |  |  |  |  |  |  |  |
| Unadjusted | 0.6 (0.03) | 0.27 | 0.7 (0.03) | 0.30 | 0.7 (0.03) | 0.26 | 0.6 (0.03) | 0.28 | 0.6 (0.04) | 0.24 |
| Sex and age | 0.4 (0.03) | 0.28 | 0.5 (0.04) | 0.31 | 0.5 (0.04) | 0.28 | 0.5 (0.04) | 0.29 | 0.4 (0.05) | 0.25 |
| Multivariate | 0.4 (0.03) | 0.29 | 0.5 (0.04) | 0.32 | 0.5 (0.04) | 0.28 | 0.5 (0.04) | 0.30 | 0.4 (0.05) | 0.26 |
| SFA, g/d |  |  |  |  |  |  |  |  |  |  |
| Unadjusted | 0.4 (0.03) | 0.22 | 0.5 (0.04) | 0.18 | 0.5 (0.03) | 0.21 | 0.4 (0.06) | 0.19 | 0.4 (0.1) | 0.13 |
| Sex and age | 0.3 (0.03) | 0.23 | 0.4 (0.05) | 0.19 | 0.3 (0.04) | 0.22 | 0.3 (0.06) | 0.20 | 0.2 (0.1) | 0.14 |
| Multivariate | 0.3 (0.03) | 0.24 | 0.4 (0.05) | 0.20 | 0.3 (0.04) | 0.23 | 0.3 (0.06) | 0.20 | 0.2 (0.1) | 0.15 |
| MUFA, g/d |  |  |  |  |  |  |  |  |  |  |
| Unadjusted | 0.6 (0.03) | 0.25 | 0.7 (0.03) | 0.37 | 0.7 (0.03) | 0.28 | 0.7 (0.03) | 0.33 | 0.6 (0.03) | 0.26 |
| Sex and age | 0.5 (0.04) | 0.26 | 0.6 (0.06) | 0.37 | 0.5 (0.04) | 0.29 | 0.6 (0.05) | 0.33 | 0.5 (0.03) | 0.26 |
| Multivariate | 0.5 (0.04) | 0.27 | 0.6 (0.07) | 0.38 | 0.5 (0.05) | 0.29 | 0.6 (0.06) | 0.34 | 0.5 (0.04) | 0.27 |
| PUFA, g/d |  |  |  |  |  |  |  |  |  |  |
| Unadjusted | 0.7 (0.03) | 0.27 | 0.8 (0.03) | 0.30 | 0.8 (0.03) | 0.31 | 0.7 (0.03) | 0.29 | 0.7 (0.03) | 0.26 |
| Sex and age | 0.5 (0.03) | 0.28 | 0.6 (0.04) | 0.32 | 0.6 (0.05) | 0.32 | 0.6 (0.05) | 0.30 | 0.5 (0.03) | 0.28 |
| Multivariate | 0.5 (0.04) | 0.30 | 0.6 (0.04) | 0.32 | 0.6 (0.05) | 0.32 | 0.5 (0.05) | 0.31 | 0.5 (0.03) | 0.29 |
| Cholesterol, mg/d |  |  |  |  |  |  |  |  |  |  |
| Unadjusted | 0.2 (0.05) | 0.13 | 0.3 (0.06) | 0.16 | 0.5 (0.06) | 0.12 | 0.4 (0.05) | 0.17 | 0.4 (0.05) | 0.13 |
| Sex and age | 0.2 (0.06) | 0.13 | 0.3 (0.06) | 0.16 | 0.4 (0.07) | 0.12 | 0.4 (0.06) | 0.17 | 0.4 (0.06) | 0.13 |
| Multivariate | 0.2 (0.06) | 0.13 | 0.3 (0.06) | 0.15 | 0.4 (0.07) | 0.12 | 0.4 (0.07) | 0.18 | 0.4 (0.06) | 0.14 |
| Fiber, g/d |  |  |  |  |  |  |  |  |  |  |
| Unadjusted | 0.7 (0.02) | 0.34 | 0.8 (0.02) | 0.41 | 0.8 (0.02) | 0.42 | 0.7 (0.02) | 0.38 | 0.7 (0.02) | 0.35 |
| Sex and age | 0.3 (0.02) | 0.45 | 0.4 (0.02) | 0.50 | 0.4 (0.03) | 0.52 | 0.4 (0.02) | 0.48 | 0.4 (0.03) | 0.44 |
| Multivariate | 0.4 (0.02) | 0.48 | 0.5 (0.02) | 0.53 | 0.4 (0.03) | 0.53 | 0.4 (0.02) | 0.50 | 0.4 (0.03) | 0.46 |
|  |  |  |  |  |  |  |  |  |  |  |
| **Vitamins** |  |  |  |  |  |  |  |  |  |  |
| Vitamin A, μg RAE/d |  |  |  |  |  |  |  |  |  |  |
| Unadjusted | 0.4 (0.05) | 0.08 | 0.5 (0.06) | 0.17 | 0.5 (0.06) | 0.10 | 0.5 (0.05) | 0.10 | 0.5 (0.05) | 0.13 |
| Sex and age | 0.3 (0.05) | 0.08 | 0.5 (0.08) | 0.17 | 0.4 (0.07) | 0.10 | 0.4 (0.06) | 0.11 | 0.4 (0.06) | 0.13 |
| Multivariate | 0.3 (0.05) | 0.09 | 0.5 (0.08) | 0.18 | 0.4 (0.07) | 0.11 | 0.4 (0.06) | 0.11 | 0.4 (0.06) | 0.14 |
| Vitamin D, μg/d |  |  |  |  |  |  |  |  |  |  |
| Unadjusted | 0.6 (0.08) | 0.21 | 0.6 (0.08) | 0.22 | 0.7 (0.07) | 0.19 | 0.5 (0.06) | 0.13 | 0.6 (0.07) | 0.18 |
| Sex and age | 0.6 (0.09) | 0.21 | 0.5 (0.08) | 0.22 | 0.6 (0.08) | 0.19 | 0.4 (0.06) | 0.13 | 0.6 (0.08) | 0.18 |
| Multivariate | 0.6 (0.09) | 0.22 | 0.5 (0.08) | 0.22 | 0.7 (0.08) | 0.19 | 0.4 (0.06) | 0.14 | 0.6 (0.08) | 0.19 |
| Vitamin E, mg/d |  |  |  |  |  |  |  |  |  |  |
| Unadjusted | 0.6 (0.02) | 0.27 | 0.7 (0.03) | 0.36 | 0.7 (0.02) | 0.33 | 0.7 (0.03) | 0.32 | 0.7 (0.02) | 0.31 |
| Sex and age | 0.5 (0.03) | 0.29 | 0.6 (0.03) | 0.37 | 0.6 (0.04) | 0.34 | 0.5 (0.04) | 0.33 | 0.5 (0.03) | 0.33 |
| Multivariate | 0.5 (0.03) | 0.31 | 0.6 (0.03) | 0.38 | 0.6 (0.04) | 0.35 | 0.5 (0.04) | 0.34 | 0.5 (0.03) | 0.34 |
| Thiamine, mg/d |  |  |  |  |  |  |  |  |  |  |
| Unadjusted | 0.6 (0.02) | 0.30 | 0.7 (0.02) | 0.38 | 0.7 (0.02) | 0.38 | 0.7 (0.02) | 0.33 | 0.7 (0.02) | 0.33 |
| Sex and age | 0.3 (0.03) | 0.38 | 0.4 (0.03) | 0.45 | 0.4 (0.03) | 0.45 | 0.4 (0.03) | 0.41 | 0.4 (0.03) | 0.40 |
| Multivariate | 0.4 (0.03) | 0.41 | 0.5 (0.03) | 0.46 | 0.5 (0.03) | 0.46 | 0.4 (0.03) | 0.42 | 0.4 (0.03) | 0.41 |
| Riboflavin, mg/d |  |  |  |  |  |  |  |  |  |  |
| Unadjusted | 0.4 (0.02) | 0.25 | 0.6 (0.02) | 0.34 | 0.6 (0.02) | 0.31 | 0.5 (0.02) | 0.25 | 0.5 (0.03) | 0.31 |
| Sex and age | 0.3 (0.02) | 0.28 | 0.4 (0.03) | 0.38 | 0.3 (0.03) | 0.37 | 0.3 (0.03) | 0.29 | 0.4 (0.04) | 0.32 |
| Multivariate | 0.3 (0.02) | 0.30 | 0.4 (0.03) | 0.40 | 0.3 (0.03) | 0.37 | 0.3 (0.03) | 0.30 | 0.4 (0.04) | 0.33 |
| Niacin, mg/d |  |  |  |  |  |  |  |  |  |  |
| Unadjusted | 0.7 (0.02) | 0.47 | 0.8 (0.02) | 0.52 | 0.9 (0.01) | 0.53 | 0.8 (0.02) | 0.50 | 0.8 (0.02) | 0.48 |
| Sex and age | 0.5 (0.02) | 0.54 | 0.6 (0.02) | 0.58 | 0.6 (0.02) | 0.59 | 0.5 (0.02) | 0.56 | 0.5 (0.03) | 0.54 |
| Multivariate | 0.5 (0.02) | 0.56 | 0.6 (0.02) | 0.59 | 0.6 (0.02) | 0.60 | 0.6 (0.02) | 0.58 | 0.6 (0.03) | 0.56 |
| Vitamin B6, mg/d |  |  |  |  |  |  |  |  |  |  |
| Unadjusted | 0.7 (0.02) | 0.45 | 0.8 (0.02) | 0.53 | 0.8 (0.02) | 0.54 | 0.8 (0.02) | 0.48 | 0.8 (0.02) | 0.47 |
| Sex and age | 0.5 (0.03) | 0.48 | 0.7 (0.03) | 0.56 | 0.7 (0.03) | 0.56 | 0.6 (0.03) | 0.51 | 0.6 (0.03) | 0.49 |
| Multivariate | 0.6 (0.03) | 0.55 | 0.7 (0.03) | 0.58 | 0.7 (0.03) | 0.58 | 0.6 (0.03) | 0.54 | 0.6 (0.03) | 0.52 |
| Folate, μg/d |  |  |  |  |  |  |  |  |  |  |
| Unadjusted | 0.5 (0.02) | 0.18 | 0.6 (0.02) | 0.22 | 0.6 (0.02) | 0.23 | 0.6 (0.03) | 0.17 | 0.6 (0.02) | 0.20 |
| Sex and age | 0.3 (0.03) | 0.22 | 0.4 (0.03) | 0.26 | 0.4 (0.04) | 0.28 | 0.3 (0.03) | 0.21 | 0.3 (0.03) | 0.23 |
| Multivariate | 0.3 (0.03) | 0.24 | 0.4 (0.03) | 0.28 | 0.4 (0.04) | 0.29 | 0.3 (0.03) | 0.22 | 0.3 (0.03) | 0.24 |
| Vitamin C, mg/d |  |  |  |  |  |  |  |  |  |  |
| Unadjusted | 0.4 (0.03) | 0.10 | 0.5 (0.03) | 0.13 | 0.5 (0.03) | 0.14 | 0.4 (0.04) | 0.12 | 0.4 (0.03) | 0.09 |
| Sex and age | 0.2 (0.04) | 0.13 | 0.3 (0.03) | 0.16 | 0.3 (0.04) | 0.16 | 0.2 (0.05) | 0.15 | 0.2 (0.04) | 0.10 |
| Multivariate | 0.3 (0.04) | 0.14 | 0.3 (0.03) | 0.17 | 0.3 (0.04) | 0.17 | 0.2 (0.05) | 0.16 | 0.2 (0.04) | 0.11 |
|  |  |  |  |  |  |  |  |  |  |  |
| **Minerals** |  |  |  |  |  |  |  |  |  |  |
| Calcium, mg/d |  |  |  |  |  |  |  |  |  |  |
| Unadjusted | 0.4 (0.03) | 0.16 | 0.7 (0.04) | 0.23 | 0.6 (0.05) | 0.20 | 0.5 (0.03) | 0.24 | 0.5 (0.03) | 0.28 |
| Sex and age | 0.3 (0.04) | 0.17 | 0.5 (0.06) | 0.24 | 0.4 (0.06) | 0.20 | 0.4 (0.04) | 0.26 | 0.4 (0.05) | 0.28 |
| Multivariate | 0.3 (0.04) | 0.18 | 0.5 (0.06) | 0.24 | 0.4 (0.06) | 0.21 | 0.4 (0.04) | 0.26 | 0.4 (0.05) | 0.29 |
| Iron, mg/d |  |  |  |  |  |  |  |  |  |  |
| Unadjusted | 0.6 (0.02) | 0.30 | 0.7 (0.02) | 0.30 | 0.7 (0.02) | 0.38 | 0.7 (0.02) | 0.32 | 0.7 (0.02) | 0.34 |
| Sex and age | 0.4 (0.03) | 0.36 | 0.5 (0.03) | 0.34 | 0.5 (0.03) | 0.43 | 0.4 (0.03) | 0.38 | 0.4 (0.03) | 0.40 |
| Multivariate | 0.4 (0.03) | 0.39 | 0.5 (0.03) | 0.36 | 0.5 (0.03) | 0.44 | 0.4 (0.03) | 0.39 | 0.4 (0.03) | 0.41 |
| Sodium, mg/d |  |  |  |  |  |  |  |  |  |  |
| Unadjusted | 0.4 (0.02) | 0.07 | 0.5 (0.02) | 0.06 | 0.5 (0.02) | 0.08 | 0.5 (0.02) | 0.08 | 0.5 (0.02) | 0.11 |
| Sex and age | 0.2 (0.02) | 0.12 | 0.2 (0.03) | 0.11 | 0.2 (0.02) | 0.15 | 0.2 (0.03) | 0.14 | 0.2 (0.03) | 0.18 |
| Multivariate | 0.2 (0.02) | 0.13 | 0.2 (0.03) | 0.13 | 0.2 (0.02) | 0.16 | 0.2 (0.03) | 0.15 | 0.2 (0.03) | 0.20 |
| Potassium, mg/d |  |  |  |  |  |  |  |  |  |  |
| Unadjusted | 0.5 (0.02) | 0.30 | 0.7 (0.02) | 0.33 | 0.7 (0.02) | 0.37 | 0.6 (0.02) | 0.33 | 0.6 (0.02) | 0.32 |
| Sex and age | 0.3 (0.02) | 0.40 | 0.4 (0.02) | 0.42 | 0.4 (0.03) | 0.46 | 0.3 (0.02) | 0.42 | 0.4 (0.03) | 0.38 |
| Multivariate | 0.3 (0.02) | 0.43 | 0.4 (0.02) | 0.43 | 0.4 (0.03) | 0.46 | 0.4 (0.02) | 0.43 | 0.4 (0.03) | 0.40 |
| Magnesium, mg/d |  |  |  |  |  |  |  |  |  |  |
| Unadjusted | 0.7 (0.02) | 0.36 | 0.8 (0.02) | 0.44 | 0.8 (0.02) | 0.45 | 0.7 (0.02) | 0.41 | 0.7 (0.02) | 0.41 |
| Sex and age | 0.3 (0.02) | 0.49 | 0.5 (0.02) | 0.52 | 0.4 (0.03) | 0.55 | 0.4 (0.02) | 0.51 | 0.4 (0.03) | 0.49 |
| Multivariate | 0.4 (0.02) | 0.52 | 0.5 (0.02) | 0.55 | 0.4 (0.03) | 0.57 | 0.4 (0.02) | 0.53 | 0.4 (0.02) | 0.51 |
| Zinc, mg/d |  |  |  |  |  |  |  |  |  |  |
| Unadjusted | 0.7 (0.02) | 0.40 | 0.8 (0.02) | 0.47 | 0.8 (0.02) | 0.48 | 0.8 (0.02) | 0.42 | 0.7 (0.02) | 0.39 |
| Sex and age | 0.4 (0.02) | 0.51 | 0.5 (0.02) | 0.55 | 0.5 (0.03) | 0.56 | 0.4 (0.02) | 0.52 | 0.4 (0.03) | 0.48 |
| Multivariate | 0.4 (0.02) | 0.54 | 0.5 (0.02) | 0.57 | 0.5 (0.03) | 0.58 | 0.4 (0.02) | 0.53 | 0.4 (0.03) | 0.50 |

All p-values are <0.001.

On the basis of linear regression models with 24-hour diet recall (24hR) intakes as the dependent variable and individualized Adult Male Equivalent (AME) or per capita (PC) consumption estimates from household questionnaire as the independent variable. The sex and age model was categorized as follows: age, ≤5, 6-10, 11-19, 20-44, and ≥45 years; sex, men and women. The multivariate model was adjusted for the following covariates: age (≤5, 6-10, 11-19, 20-44, and ≥45 years), sex (men, women), education (<6 years of education, ≥6 years of education), religion (Muslims, other), respondent’s age (continuous), sex (men, women) and education (<6 years of education, ≥6 years of education), household size, number of children within household, and food wastage percentage (using 24hR data, we calculated for each household, the percent of food wastage -sum of food waste, and food given to guests, others and animals- to total consumed food (mean: 11.6%, SD: 13.6)). *Β*s represent the change in the individual intake (24hR) for every unit increase in the AME mean. SEs for *β*s are presented. R^2^ represents the coefficient of determination for the overall model.

BIHS, Bangladesh Integrated Household Survey; MUFA, Monounsaturated fats; PUFA, Polyunsaturated fats; SFA, Saturated fat

# **
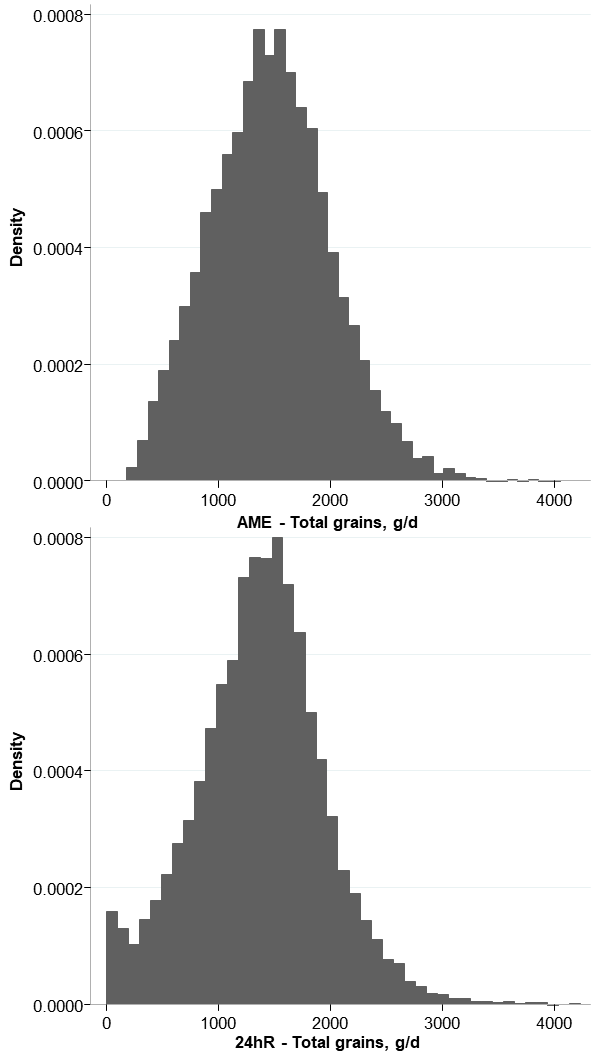
**Figure A. Distribution of individualized household estimates and 24-hour recall intakes for selected dietary factors in the overall population in the 2011-2012 BIHS.


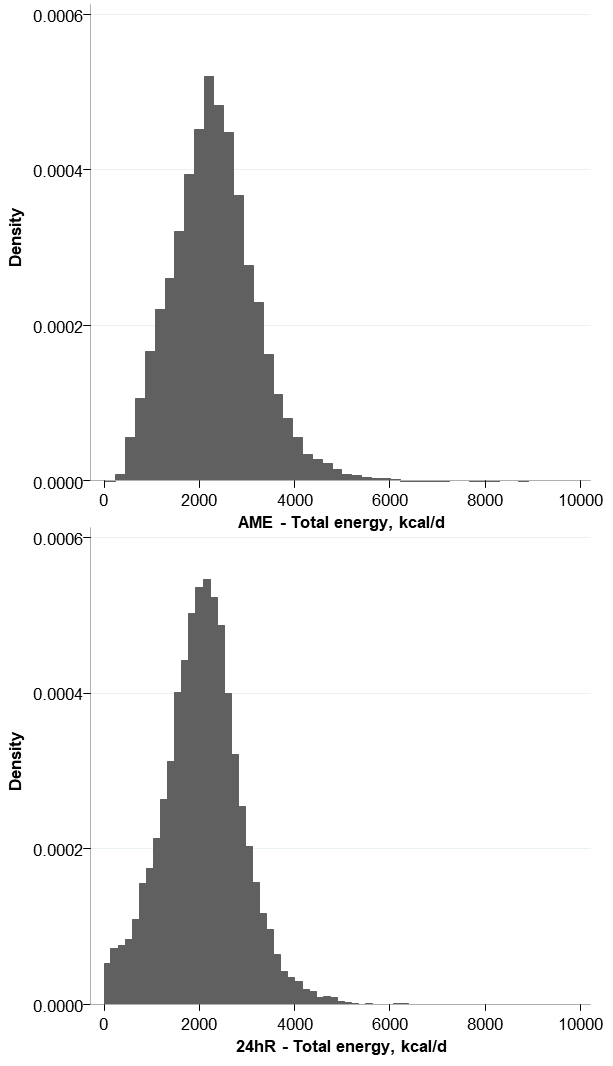


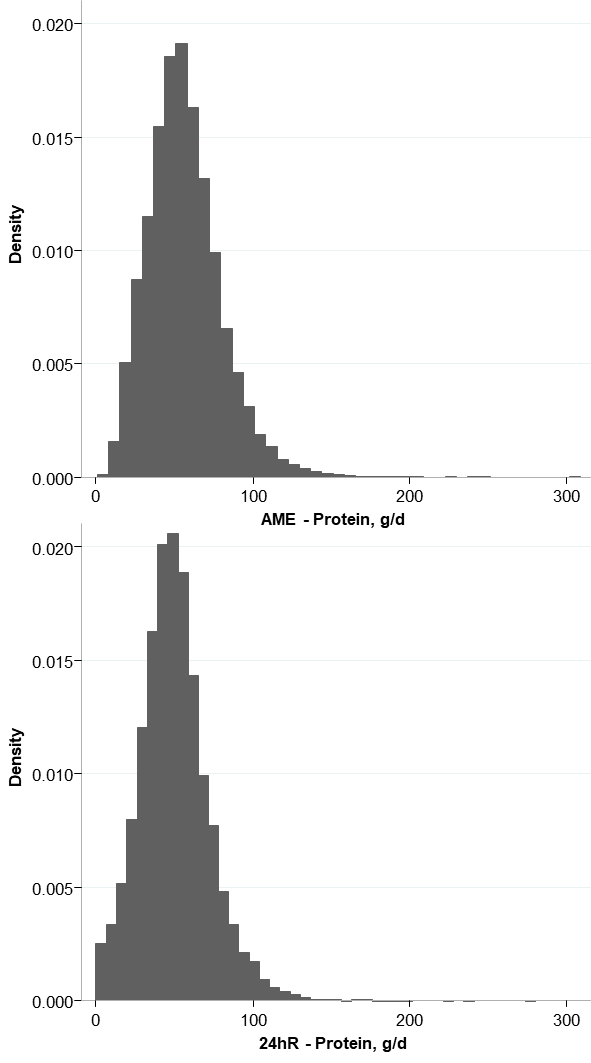

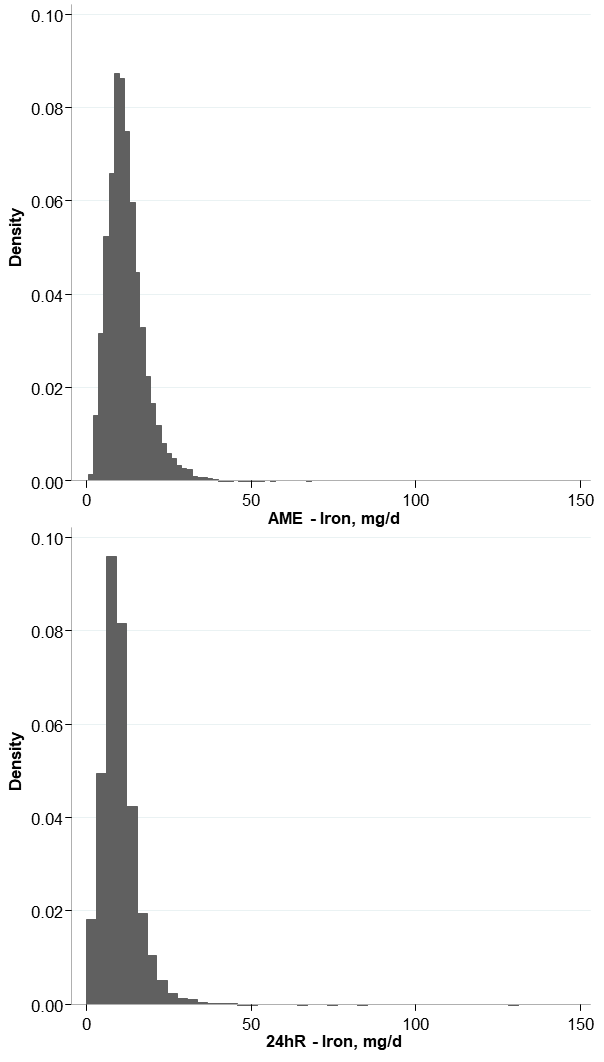


Distribution of individualized dietary consumption estimated from household survey data by the Adult Male Equivalent (AME) approach (Appendix B) and individual-level 24-hr dietary recall (24hR) intakes are presented for the overall population. Intakes are presented in g/d for foods, and in mg/d for nutrients.

# References

1. Charrondiere UR, Rittenschober D, Nowak V, Stadlmayr B, Wijesinha-Bettoni R, Haytowitz D. Improving food composition data quality: Three new FAO/INFOODS guidelines on conversions, data evaluation and food matching. *Food chemistry.* Feb 15 2016;193:75-81.

2. Shaheen N, Rahim ATM, Mohiduzzaman M, et al. *Food composition table for Bangladesh.* 1 ed: Institute of Nutrition and Food Science, Centre for Advanced Research in Sciences, University of Dhaka; 2013.

3. USDA National Nutrient Database for Standard Reference, Release 28. Version Current. 2015. https://ndb.nal.usda.gov/ndb/.

4. Bognar A. Tables on weight yield of food and retention factors of food constituents for the calculation of nutrient composition of cooked foods (dishes). 2002.

5. U.S. Department of Agriculture. *USDA Table of Nutrient Retention Factors. Release 6.* 2007.

6. FAO/INFOODS. *Guidelines for Food Matching. Version 1.2.* 2012. FAO, Rome.

7. Islam R. *Consumption of Unsafe Foods: Evidence from Heavy Metal, Mineral and Trace Element Contamination.* Department of Soil Science, Bangladesh Agricultural University;2013.

8. U.S. Department of Agriculture. Food yields. Summarized by different stages of preparation. *Agriculture Handbook.* 1975;102.

9. Del Gobbo LC, Khatibzadeh S, Imamura F, et al. Assessing global dietary habits: a comparison of national estimates from the FAO and the Global Dietary Database. *The American journal of clinical nutrition.* May 2015;101(5):1038-1046.

10. European Food Safety Authority. *The food classification and description system FoodEx 2 (draft-revision 1).* Parma, Italy2011.

11. European Food Safety Authority. Guidance on the EU Menu methodology. *EFSA Journal.* 2014;12(12).

12. Weisell R, Dop MC. The adult male equivalent concept and its application to Household Consumption and Expenditures Surveys (HCES). *Food Nutr Bull.* Sep 2012;33(3 Suppl):S157-162.

13. FAO. Food Balance Sheets. A handbook. 2001. FAO. Rome.

14. FAO. *Human energy requirements. Report of a Joint FAO/WHO/UNU Expert Consultation.* Rome2001.
